# Enantioselective Hydroalkoxylation of 1,3-Dienes via Ni-Catalysis

**DOI:** 10.1021/jacs.2c12779

**Published:** 2023-02-10

**Authors:** Qi Li, Zhen Wang, Vy M. Dong, Xiao-Hui Yang

**Affiliations:** † Advanced Research Institute of Multidisciplinary Science, School of Chemistry and Chemical Engineering, Key Laboratory of Medical Molecule Science and Pharmaceutical Engineering, Ministry of Industry and Information Technology, Beijing Institute of Technology, Beijing 100081, P. R. China; ‡ Department of Chemistry, University of California−Irvine, Irvine, California 92697, United States

## Abstract

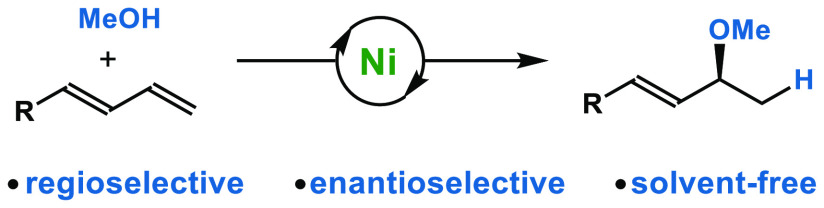

As an advance in hydrofunctionalization, we herein report that alcohols add to 1,3-dienes with high regio- and enantioselectivity. Using Ni-DuPhos, we access enantioenriched allylic ethers. Through the choice of solvent-free conditions, we control the reversibility of C–O bond formation. This work showcases a rare example of methanol as a reagent in asymmetric synthesis.

Drawing inspiration from ether-containing pharmaceuticals,^1^ agrochemicals,^2^ and natural products,^3^ chemists strive to identify useful C–O bond-forming methods. Hydrofunctionalization represents an attractive approach to construct C–X bonds from feedstock olefins.^4^ In contrast to carbon- and nitrogen-based nucleophiles, chalcogen nucleophiles are underdeveloped as coupling partners.^5^ In most cases, alkynes or allenes have been used as substrates for hydroalkoxylation, with high regioselectivity and enantioselectivity,^5a,6^ albeit using precious metal catalysts, such as Rh,^7^ Ru,^8^ Pd,^9^ or Au^10^ (Figure 1). The asymmetric hydroalkoxylation of readily available dienes has attracted attention and warrants further studies, especially using earth-abundant catalysts.^11^ With Ni-catalysis, Mazet and co-workers demonstrated the promising addition of alcohols to 2-substituted 1,3-dienes to yield racemic allylic ethers (Figure 1).^11c^ By applying a chiral phosphinooxazoline ligand, they achieved an isolated enantioselective example. However, they observed a decreasing enantiomeric ratio during the course of the experiment. Sauthier and co-workers disclosed a Ni-catalyzed enantioselective hydroalkoxylation of butadiene using ethanol; racemization and isomerization were also observed (Figure 1).^11b,11d^ Through our independent investigations, we discovered a complementary and enantioselective Ni-catalyzed hydroalkoxylation of dienes. Petroleum feedstocks and readily available dienes can be transformed into chiral allylic ether building blocks with high regio- and enantiocontrol via Ni-catalysis under solvent-free conditions (Figure 1).^12^

**Figure 1:**
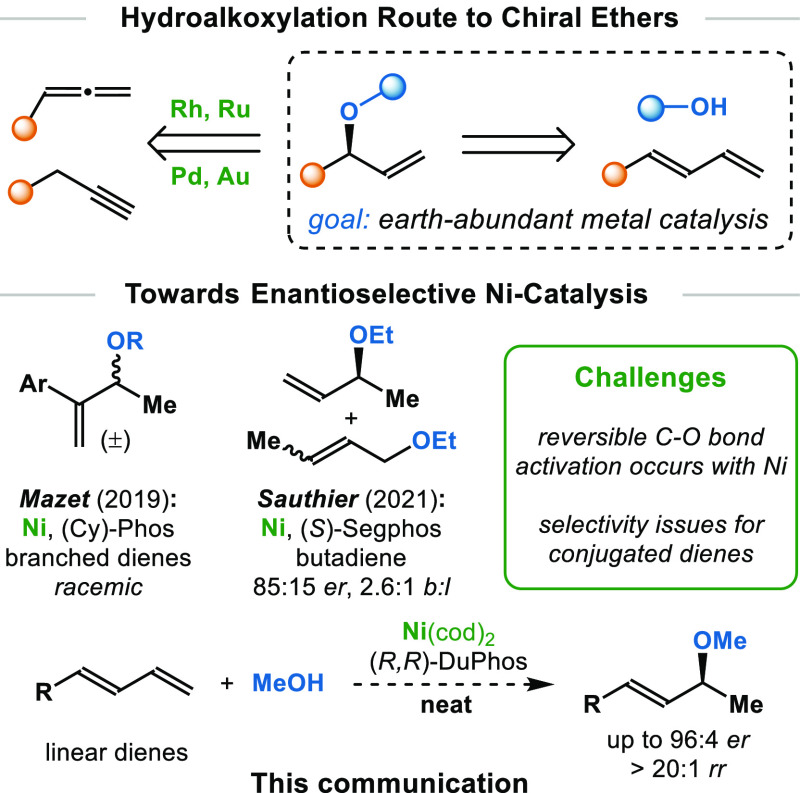
Proposal for hydroalkoxylation of 1,3-dienes.

Our laboratory has pursued the hydrofunctionalization of 1,3-dienes, including hydroamination,^13^ hydrothiolation,^14^ and hydrophosphinylation.^15^ In these reports, conjugated dienes could be transformed via metal−π-allyl intermediates to produce the corresponding 1,2- and/or 1,4-addition products. Compared to amines (with nucleophilicity *N* = 13.2 on the Mayr scale^16^) and thiols (*N* = 23.4), alcohols (*N* = 9.6) present a unique challenge and opportunity due to their lower nucleophilicity.

With this challenge in mind, we chose methanol (**1a**) and 1-phenyl-1,3-butadiene (**2a**) as the model substrates and surveyed a wide range of metal catalysts. We found that the desired branched allylic ether (**3aa**) was obtained by using Ni-catalysis with ethereal solvents. We studied the hydroalkoxylation of diene **2a** with methanol (**1a**) using different bidentate phosphine ligands in the presence of Ni(cod)_2_ (Table 1). With JosiPhos (**L1**), BINAP (**L2**), and SKP (**L3**) ligands, no product formation was detected. Yet, the BPE (**L4**) and DuPhos families (**L5** and **L6**) afforded promising results. With **L5** as the ligand, we obtained excellent regioselectivity for the allylic ether **3aa** (>20:1 *rr*) with 14% yield and 92:8 *er* by using ^*i*^Pr_2_O; other ethereal solvents (such as THF or cyclopentyl methyl ether) showed lower reactivity and enantioselectivity. The linear diene **2a** showed no reactivity under the conditions previously reported by Mazet.^11c^ However, in accordance with studies by Mazet^11c^ and Sauthier,^11b,11d^ we found that the enantioselectivity decreased dramatically with prolonged reaction times. To our delight, we discovered that this decrease in enantioselectivity over time could be overcome by performing the experiment neat (i.e., without solvent). Under solvent-free conditions, we isolated the enantioenriched ether **3aa** in 75% yield and 91:9 *er*. When the temperature was lowered to 0 °C, the enantioselectivity was increased to 96:4 *er* with excellent yield (95%, 4 h). Furthermore, the catalyst loading could be decreased to 2.5 mol% (94% yield, 96:4 *er*, 10 h). This represents a rare example of methanol as a reagent in asymmetric synthesis.^17^

**Table 1:**
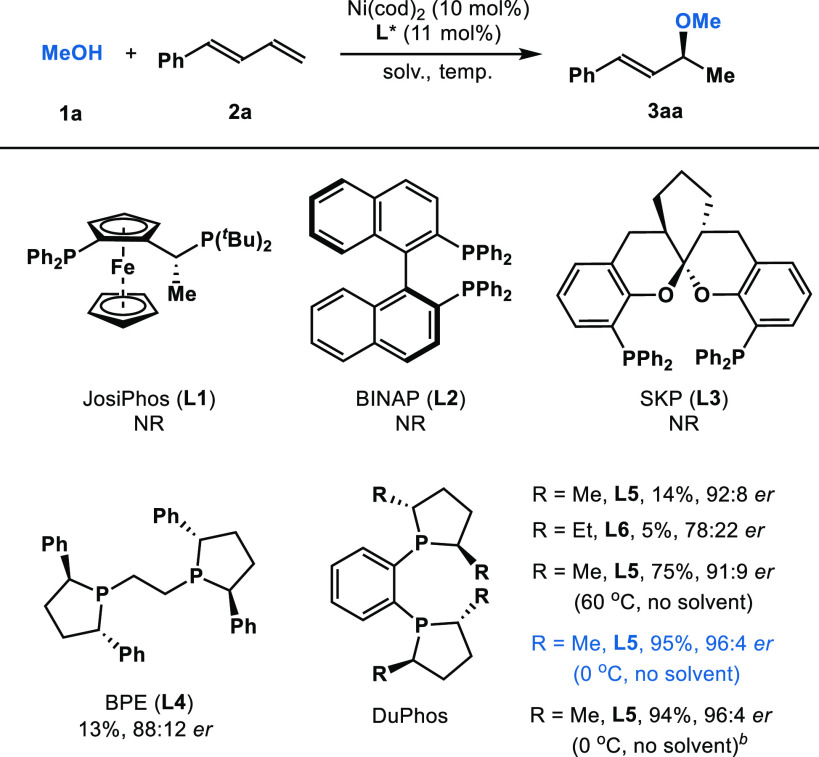
Survey of Ligands and Conditions^a^

a Reaction conditions: **1a** (0.1 mmol), **2a** (0.3 mmol), Ni(cod)_2_ (10 mol%), ligand (11 mol%), ^*i*^Pr_2_O (0.1 mL), 60 °C, 4 h. Isolated yields. Enantiomeric ratio (*er*) was determined by HPLC.

b Using 2.5 mol% Ni(cod)_2_ and 2.8 mol% **L5**, 10 h.

With these conditions in hand, we investigated the hydroalkoxylation of various 1,3-dienes with methanol **1a** (Table 2). Products bearing both electron-donating and electron-withdrawing groups on the phenyl ring were obtained with high reactivities and enantioselectivities (**3ba**–**3ha**, 66–94% yield, 81:19–96:4 *er*). This protocol tolerates heterocycle-substituted 1,3-dienes such as **2i** (R^1^ = 2-furyl) and **2j** (R^1^ = 2-thienyl) to afford the corresponding allylic ethers **3ia** (92% yield, 95:5 *er*) and **3ja** (65% yield, 93:7 *er*). In addition, hydroalkoxylation of alkyl-substituted 1,3-diene **2k** and feedstock butadiene **2l** gave the corresponding products **3ka** and **3la** in 31% and 48% yields with 88:12 *er* and 80:20 *er*, respectively.^18^ Moreover, addition of methanol (**1a**) to branched diene **2m** provided the allylic ether **3ma** in 77% yield with 62:38 *er* and >20:1 *rr*. Overall, these results demonstrate the first asymmetric hydroalkoxylation of dienes without erosion of the enantiomeric ratio.

**Table 2:**
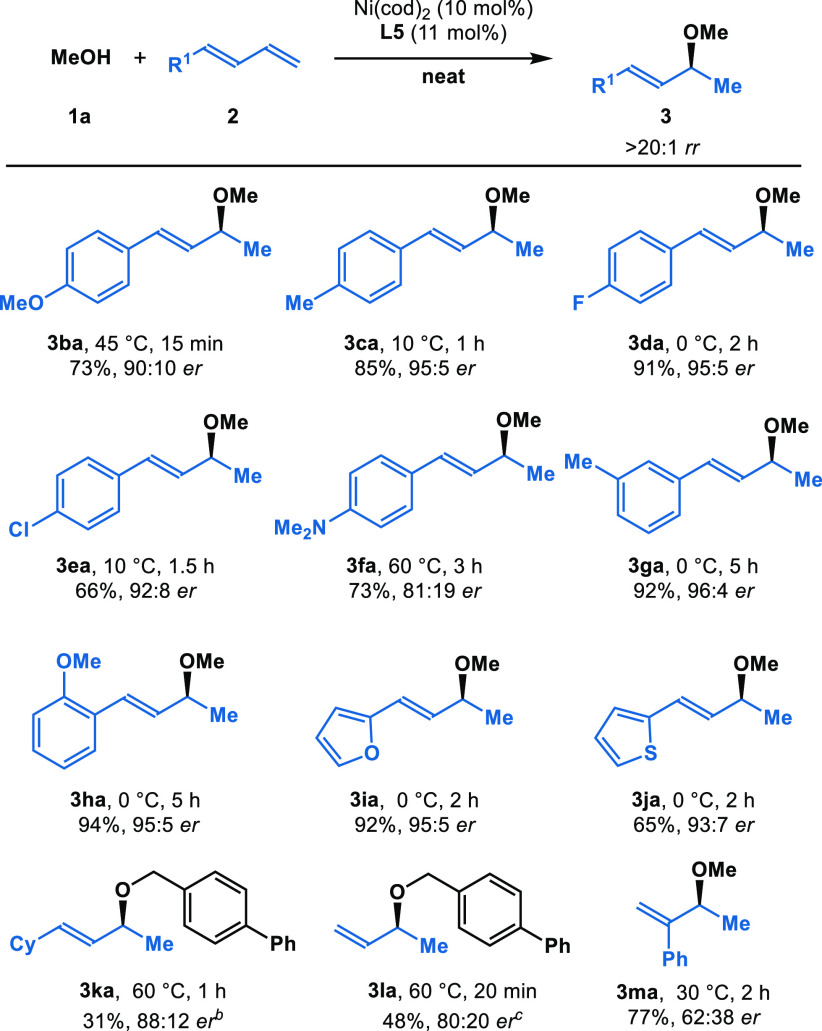
Hydroalkoxylation of Various Dienes^a^

a Reaction conditions: **1a** (0.2 mmol), **2** (0.6 mmol), Ni(cod)_2_ (10 mol%), **L5** (11 mol%). Isolated yields. Enantiomeric ratio (*er*) is determined by HPLC.

b ^*i*^Pr_2_O (2 M) as solvent.

c Butadiene (2.0 mmol) in hexane (20%) is used.

Next, we examined the addition of various alcohols **1** to diene **2a** (Table 3). We found that a variety of alcohols could be transformed into chiral ethers with good reactivity and selectivity. High reactivities (60–95% yield) and enantioselectivities (91:9–96:4 *er*) are obtained by using alcohols that bear phenyl, chloro, and trimethylsilyl groups (**3ab**–**3aj**). Hydroalkoxylation of diene (**2a**) with natural product (−)-citronellol (**1k**) furnishes the desired ether (*S*,*S*)-**3ak** in 73% yield with >20:1 *dr*. Alcohols such as isopropanol and *tert*-butanol showed no reactivity. Hydroalkoxylation with secondary alcohols, such as cyclopropanol (**1l**) and cyclopentanol (**1m**), provide the corresponding allylic ethers **3al** and **3am** with high efficiency (88% and 65% yield, respectively) and enantioselectivities (97:3 *er* and 91:9 *er*, respectively). In all cases, only one constitutional isomer is obtained.^19^

**Table 3:**
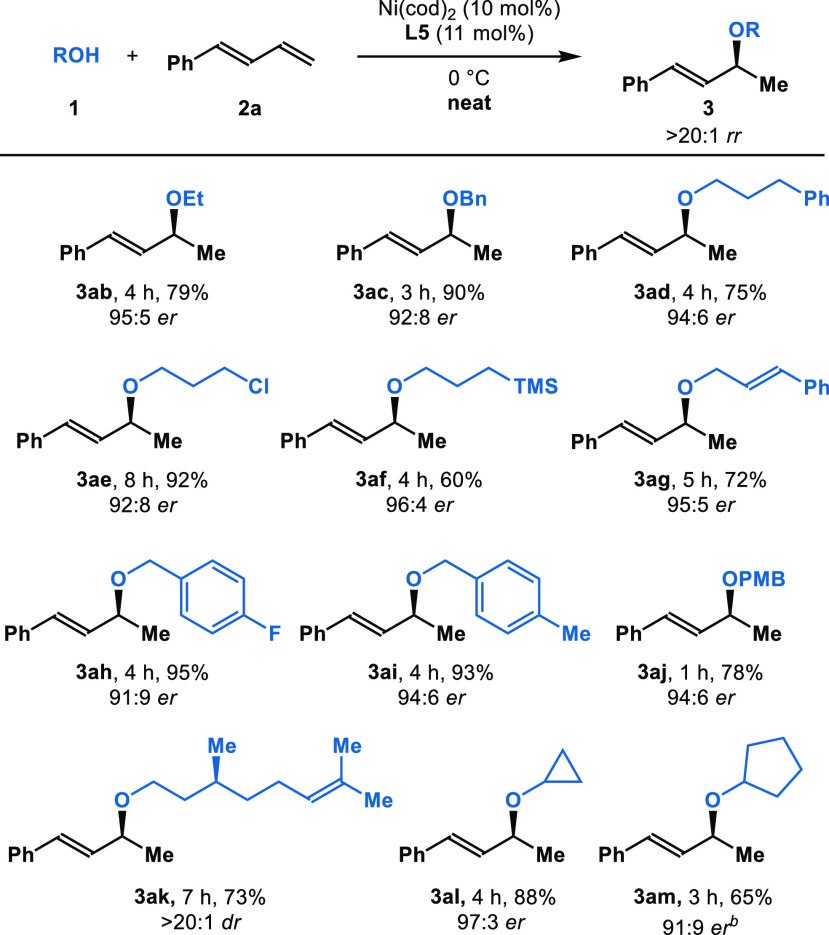
Hydroalkoxylation with Various Alcohols^a^

a Reaction conditions: **1** (0.2 mmol), **2a** (0.6 mmol), Ni(cod)_2_ (10 mol%), **L5** (11 mol%), 0 °C. Isolated yields. Enantiomeric ratio (*er*) is determined by HPLC.

b 60 °C. PMB = *p*-methoxybenzyl.

On the basis of literature reports and our own observations, we envision the following mechanistic pathway (Figure 2). Ligand exchange between 1,5-cyclooctadiene (cod) with a bidentate phosphine ligand generates intermediate **I**. Both alcohol **1** and 1,3-diene **2** bind to Ni via ligand exchange to generate nickel intermediate **II**. From here, we imagine that the hydrogen atom is transferred directly from alcohol **1** to 1,3-diene **2** through ligand-to-ligand hydrogen transfer (LLHT).^12b,12c^ In accordance with Sauthier and Macgregor’s hydroalkoxylation of butadiene, we propose a cationic allylic intermediate **III** where the alkoxide is stabilized by hydrogen bonding to the alcohol.^11d^ Intermediate **III** undergoes outer-sphere nucleophilic attack by the alkoxide at the C3 carbon to provide product **3**. Our proposed mechanism fits with the convention of classifying nucleophilic attack on η^3^-M-π-allyl intermediates for Pd^20a^ and Ni^20b^ allylations. Alkoxides, considered “soft” nucleophiles, would proceed through outer-sphere attack.

**Figure 2:**
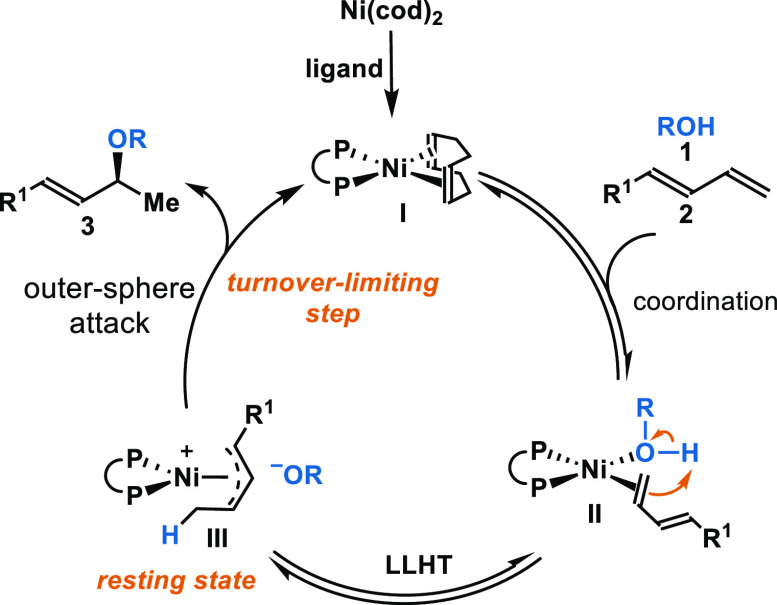
Proposed mechanism via ligand-to-ligand hydrogen transfer (LLHT).

Alternatively, mechanisms involving a Ni–H insertion have been previously proposed for Ni-catalyzed hydrofunctionalization of dienes.^12d,12i^ Inspired by Sauthier’s studies, we investigated both acid and base additives to further probe the Ni–H pathway.^11d^ While no transformation was observed in the presence of acids (e.g., TFA, xylylic acid, TsOH), hydroalkoxylation occurred in the presence of bases (e.g., Et_3_N, NaOH, ^*t*^BuONa). The observation of hydroalkoxylation under basic conditions led us to disfavor a Ni–H mechanism. We conducted ^1^H NMR experiments (at −60 °C) and did not observe Ni–H intermediates. While density functional theory (DFT) studies for this transformation are warranted, LLHT mechanisms have emerged as more energetically favorable for related Ni-catalyzed hydroarylations.^12c,21^ DFT studies by Zhou,^12c^ Dang,^21a^ and Sakai^21b^ demonstrated that the LLHT pathway was favored across different ligands, including DTBM-Segphos, SpiroAP, and *N*-heterocyclic carbene, respectively.

In the rate comparison between methanol (**1a**) and deuterated methanol (*d***-1a**) under the standard reaction conditions, a secondary (rather than primary) kinetic isotope effect (*KIE* = 1.1) is observed (Figure 3A). We postulate that outer-sphere nucleophilic attack is the turnover-limiting step. When deuterated methanol is subjected to the standard reaction conditions, deuterium is only incorporated into the terminal position of product ***d****-***3aa**, and diene with deuterium incorporation is recovered (Figure 3B). The isotopic labeling observed in the recovered diene suggests that LLHT is a reversible step. By using Burés’s variable time normalization analysis (VTNA),^22^ we studied the kinetic profile and observed a first-order dependence on catalyst and zero-order on diene. Interestingly, the order on alcohol depends on the concentration: inverse order was observed when using a higher concentration of alcohol (2.38 to 5.95 M). However, fractional order was observed when using a lower concentration (1.19 to 2.38 M).^23^ This result suggests that increasing the concentration of alcohol inhibits the outer-sphere nucleophilic attack, probably due to hydrogen bonding. On basis of these results, we postulate intermediate **III** as a catalyst resting state. Monitoring of the reaction by ^31^P NMR shows peaks that are consistent with intermediate **III**.^24^ To examine whether such a resting state is detectable, we subjected an authentic catalytic solution to electrospray ionization mass spectrometry (ESI-MS) analysis. At low voltage (Frag = 80 V), we observed a prominent signal with *m*/*z* 538.2289, which supports intermediate **III** but does not rule out the possibility of a Ni-species with OMe associated.^24^

**Figure 3:**
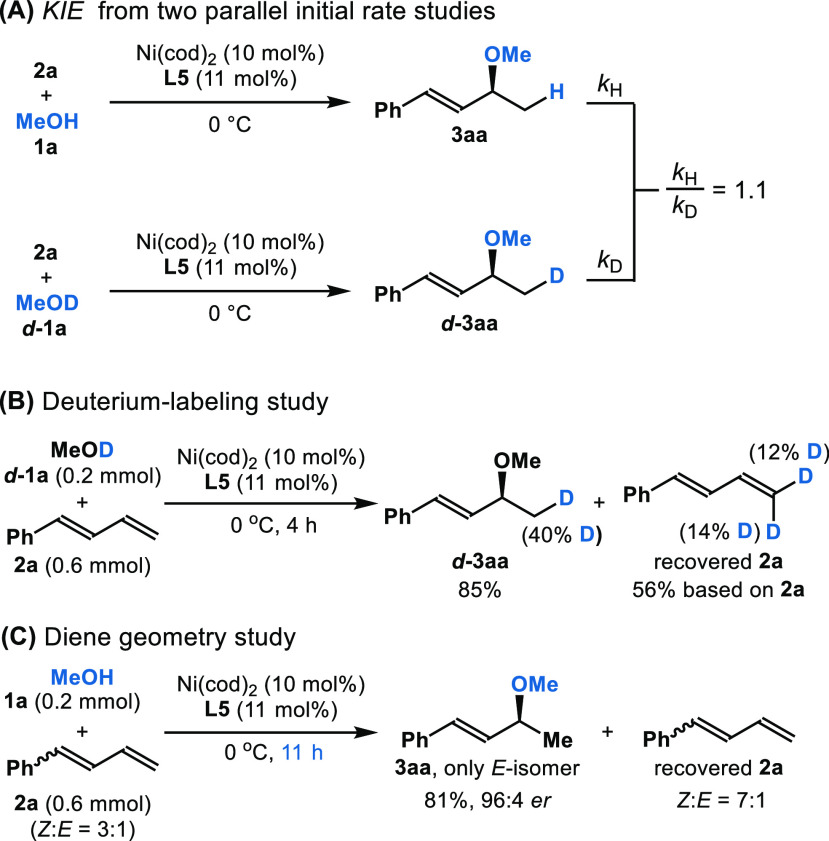
Mechanistic studies.

When using the mixture of (*Z*)- and (*E*)-**2a** (3:1, Figure 3C), the (*E*)-product **3aa** was obtained in similar yield (81%), enantioselectivity (96:4 *er*), and regioselectivity (>20:1 *rr*), albeit after 11 h. In comparison, the model substrate (*E*)-**2a** undergoes complete conversion in 4 h (Table 1, 95% yield, 96:4 *er*, >20:1 *rr*). The recovered diene remains enriched in the *Z*-isomer (7:1), which suggests isomerization is slow compared to alcohol addition.^25^

We performed crossover studies to understand the reversibility of C–O bond formation (Figure 4A). When product **3aa** was subjected to otherwise standard reaction conditions, in the presence of 1 equiv of benzyl alcohol **1c**, no trace of **3ac** was detected after 3 h; the *er* value of recovered starting material **3aa** (96:4 *er*) was constant. However, when a related crossover experiment was performed in the presence of the solvent ^*i*^Pr_2_O, we observed formation of **3ac** (91:9 *er*) and diene **2a** (13% yield). In Sauthier and Macgregor’s study on hydroalkoxylation of butadiene, the overall hydroxylation reactions are computed to be only marginally exergonic with modest barriers. This energetic profile is consistent with a reversible process at an elevated temperature (80 °C).^11b,11d^ We reason that solvent-free conditions enable transformation at lower temperature (0 °C) and thus result in a kinetically controlled process that avoids racemization.

**Figure 4:**
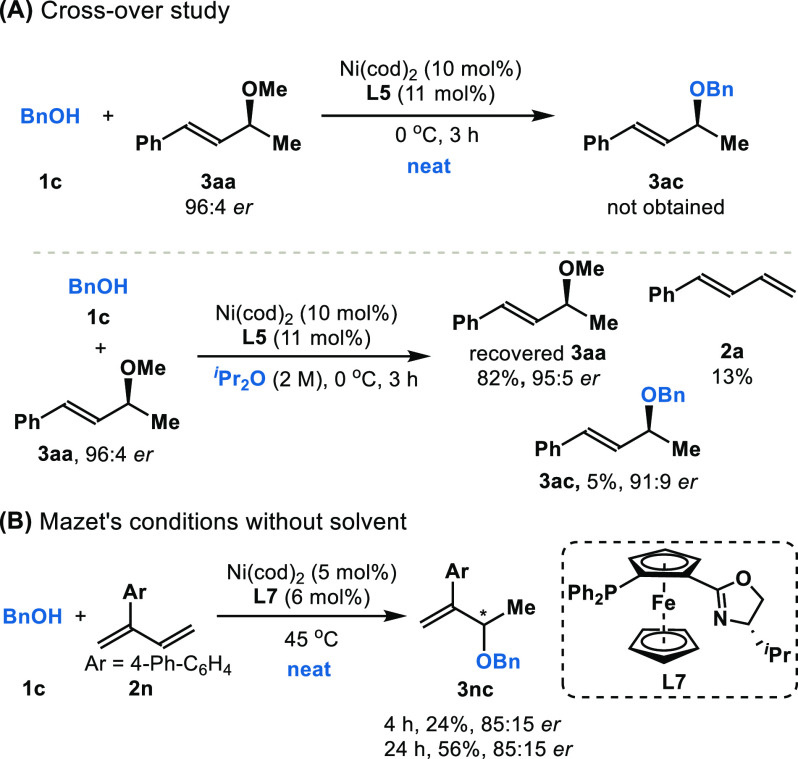
Reversibility studies.

The activation of C–O bonds under Ni-catalysis in solvent has been investigated both theoretically and experimentally.^26^ While a number of pathways are possible, we observe that the major isomer of **3ac** generated from **3aa** has the same configuration as the starting **3aa**. The net retention of stereochemistry initially observed could result from an S_N_2 pathway involving double inversion.^26g^ Alternatively, stereoretentive oxidative additions have also been observed by Watson, Jarvo, and Hong.^26a,26c,26d^ In regards to racemization, Doyle has shown the feasibility of S_N_1-like pathways.^26e^ Solvent-free conditions prevent reversible C–O bond formation, and this phenomenon may have broader applications. As an example, we investigated Mazet’s conditions for transforming **2n** to **3nc**; without solvent, we found that racemization did not occur as previously observed when mesitylene was the solvent of choice (Figure 4B).^11c^

Hydroalkoxylation represents an attractive way to transform dienes into allylic ethers. By using Ni-catalysis, we have achieved the first enantioselective hydroalkoxylation of linear dienes with various alcohols without racemization. The allylation works well with a broad range of alcohols and tolerates different functional groups such as halogens, esters, and silanes. Insights from this study will guide future olefin couplings with chalcogen nucleophiles.

## Supporting Information Available

Experimental procedures and spectral data for all new compounds (PDF)
