# Supplementary material for: Enantioselective Hydroalkoxylation of 1,3-Dienes via Ni-Catalysis
Source: J Am Chem Soc. 2023 Feb 10;145(7):3909–14. doi: 10.1021/jacs.2c12779 (PMC9951252; doi:10.1021/jacs.2c12779)
Supplement: Supplementary file 1 — ja2c12779_si_001.pdf [file ja2c12779_si_001.pdf]

Supporting Information For:

Enantioselective Hydroalkoxylation of 1, 3-Dienes via Ni-catalysis

Qi Li,[a] Zhen Wang,[a] Vy M. Dong,*[b] and Xiao-Hui Yang*[a]

[a]*Advanced Research Institute of Multidisciplinary Science, School of Chemistry and Chemical Engineering, and Key Laboratory of Medical Molecule Science and Pharmaceutical Engineering, Ministry of Industry and Information Technology, Beijing Institute of Technology, Beijing 100081, P.R. China*

[b]*Department of Chemistry, University of California−Irvine, Irvine, California 92697, United States*

xhyang@bit.edu.cn, dongv@uci.edu

Table of Contents

1. General……………………………………………………………………S2

2. Synthesis of product *rac*-3………………………………………………S3

3. General procedure for hydroalkoxylation of 1,3-dienes…………………S3

4. Mechanistic studies……………………………………………………S13

4.1. Acid/base additives for hydroalkoxylation…………………………S13

4.2. Kinetic studies…………………………………………………………S14

4.3. NMR Monitoring………………………………………………………S18

4.4. Electrospray MS (ESI-MS) analysis…………………………………S19

4.5. Initial rate *KIE* study…………………………………………………S20

4.6. Deuterium-labeling study……………………………………………S21

4.7. Diene geometry study…………………………………………………S24

4.8. Cross-over studies………………………………………………………S27

4.9. Reversibility study for branched 1,3-diene……………………………S32

5. References………………………………………………………………S35

6. NMR spectra……………………………………………………………S37

7. HPLC spectra……………………………………………………………S62

1. General

Commercial reagents were purchased from LAAJOO, Bide Pharm, Tansoole or Acros Organics and without further purification. Benzyl alcohol was purified by distillation. All experiments were performed in oven-dried or flame-dried glassware. Reactions were monitored using either thin-layer chromatography (TLC) or gas chromatography (GC) using an Agilent Technologies 7890A GC system equipped with an Agilent Technologies 5975C inert XL EI/CI MSD. Visualization of the developed plates was performed under UV light (254 nm) or $KMnO_4$ stain. Organic solutions were concentrated under reduced pressure on a Heidolph rotary evaporator. Purification and isolation of products were performed via silica gel chromatography (both column and preparative thin-layer chromatography). Solvents were purchased from Tansoole. ^1H NMR, ^2H NMR and ^{13}C NMR spectra were recorded on Bruker Ascend 400M spectrometer. ^1H NMR spectra were internally referenced to the residual solvent signal or TMS. ^{13}C NMR spectra were internally referenced to the residual solvent signal. Data for ^1H NMR are reported as follows: chemical shift (δ ppm), multiplicity (s = singlet, d = doublet, t = triplet, q = quartet, m = multiplet), coupling constant (Hz), integration. Data for ^2H NMR, ^{13}C NMR are reported in terms of chemical shift (δ ppm). Infrared (IR) spectra were obtained on a Thermo IS5 spectrometer and are reported in terms of absorption frequency (cm^{-1}). Enantiomeric ratio for enantioselective reactions were determined by a Shimadzu system High Performance Liquid Chromatography (HPLC) equipped with SPD-20A detector and LC-20AT pump. High resolution mass spectra (HRMS) were obtained on a Thermo LCQ Deca XP Maz mass spectrometer (ESI). 1,3-Dienes **2a-2k**, **2m,** and **2n** used here were known compounds and synthesized according to the reported literatures.[1-3]

2. Synthesis of product *rac*-3

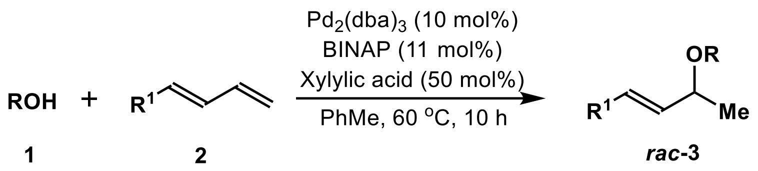

In a N_2-filled glovebox, BINAP (6.8 mg, 0.011 mmol) and PhMe (0.2 mL) were added to a 1-dram vial containing $Pd_2(dba)_3$ (4.6 mg, 0.005 mmol). The resulting mixture was stirred for 10 min and then Xylylic acid (7.5 mg, 0.05 mmol), diene **2** (0.10 mmol) and alcohol **1** (0.20 mmol) were added. The mixture was stirred at 60 °C for 10 h. The resulting solution was then cooled to rt. Products ***rac*-3** were obtained by column chromatography on silica gel or preparative thin-layer chromatography.

3. General procedure for hydroalkoxylation of 1,3-dienes

Method A:

In a N_2-filled glovebox, (*R*, *R*)-Me-DuPhos (6.7 mg, 0.022 mmol) and 1,3-diene **2** (0.6 mmol) were added to a 1-dram vial containing $Ni(cod)_2$ (5.6 mg, 0.02 mmol). The resulting mixture was stirred for 10 min and then alcohol **1** (0.2 mmol) was added. The mixture was removed from the glovebox and was held at 0 °C until no starting material was observed by TLC or GC-MS. The regioselectivities were determined by ^1H NMR analysis of the unpurified reaction mixture. Isolated yields (obtained by column chromatography on silica gel or preparative thin-layer chromatography) are reported.

Method B:

In a N_2-filled glovebox, (*R*, *R*)-Me-DuPhos (6.7 mg, 0.022 mmol) and $^iPr_2O$ (0.1 mL) were added to a 1-dram vial containing $Ni(cod)_2$ (5.6 mg, 0.02 mmol). The resulting mixture was stirred for 10 min and then 1,3-diene **2** (0.6 mmol) and alcohol **1** (0.2 mmol) were added. The mixture was held at 60 °C until no starting material was observed by TLC or GC-MS. The regioselectivities were determined by ^1H NMR analysis of the

unpurified reaction mixture. Isolated yields (obtained by column chromatography on silica gel or preparative thin-layer chromatography) are reported.

(*S,E*)-(3-methoxybut-1-en-1-yl)benzene (3aa)[4]

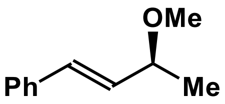

Method A (4 h), colorless oil, 95% yield, 96:4 *er*, >20:1 *rr*, $[\alpha]^{28}_{D}$ = +88.93 (c 0.52, CH_2Cl_2). **^{1}H NMR** (400 MHz, $CDCl_3$) δ 7.36 – 7.29 (m, 2H), 7.28 – 7.21 (m, 2H), 7.20 – 7.12 (m, 1H), 6.46 (d, *J* = 16.0 Hz, 1H), 6.02 (dd, *J* = 16.0, 7.6 Hz, 1H), 3.86 – 3.77 (m, 1H), 3.25 (s, 3H), 1.26 (d, *J* = 6.4 Hz, 3H). **^{13}C NMR** (101 MHz, $CDCl_3$) δ 136.8, 131.6, 131.5, 128.7, 127.8, 126.6, 78.3, 56.2, 21.7. **HPLC**: chiral stationary column OJ-H, mobile phase hexane/^{i}PrOH = 99/1, 1.0 mL/min, 254 nm, 30 °C, t_R (minor) = 6.9 min, t_R (major) = 7.4 min.

(*S,E*)-1-methoxy-4-(3-methoxybut-1-en-1-yl)benzene (3ba)[5]

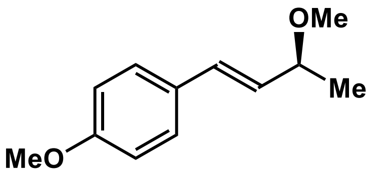

Method A (45 °C, 15 min), light yellow oil, 73% yield, 90:10 *er*, >20:1 *rr*, $[\alpha]^{28}_{D}$ = +9.30 (c 0.17., CH_2Cl_2). **^{1}H NMR** (400 MHz, $CDCl_3$) δ 7.33 (d, *J* = 8.7 Hz, 2H), 6.86 (d, *J* = 8.7 Hz, 2H), 6.48 (d, *J* = 15.9 Hz, 1H), 5.95 (dd, *J* = 15.9, 7.7 Hz, 1H), 3.91 – 3.83 (m, 1H), 3.81 (s, 3H), 3.31 (s, 3H), 1.33 (d, *J* = 6.3 Hz, 3H). **^{13}C NMR** (101 MHz, $CDCl_3$) δ 159.4, 131.1, 129.6, 129.4, 127.8, 114.2, 78.4, 56.1, 55.4, 21.7. **HPLC**: chiral stationary column AD-H, mobile phase hexane/^{i}PrOH = 99/1, 1.0 mL/min, 254 nm, 30 °C, t_R (minor) = 7.2 min, t_R (major) = 7.9 min.

(*S,E*)-1-(3-methoxybut-1-en-1-yl)-4-methylbenzene (3ca)[5]

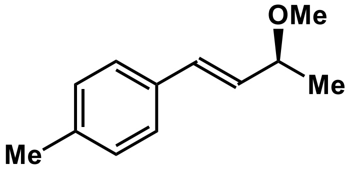

Method A (10 °C, 1 h), colorless oil, 85% yield, 95:5 *er*, >20:1 *rr*, $[\alpha]^{28}_{D}$ = +84.54 (c 0.49, CH_2Cl_2). **^{1}H NMR** (400 MHz, $CDCl_3$) δ 7.30 (d, *J* = 8.0 Hz, 2H), 7.14 (d, *J* = 7.9 Hz, 2H), 6.51 (d, *J* = 15.9 Hz, 1H), 6.05 (dd, *J* = 15.9, 7.7 Hz, 1H), 3.93 – 3.84 (m, 1H), 3.32 (s, 3H), 2.35 (s, 3H), 1.34 (d, *J* = 6.3 Hz, 3H). **^{13}C NMR** (101 MHz, $CDCl_3$) δ 137.7, 134.0, 131.5, 130.6, 129.4, 126.5, 78.4, 56.2, 21.7, 21.4. **HPLC**: chiral

stationary column OJ-H, mobile phase hexane/^{i}PrOH = 99/1, 1.0 mL/min, 254 nm, 30 °C, t_R (minor) = 7.7 min, t_R (major) = 8.4 min.

(*S,E*)-1-fluoro-4-(3-methoxybut-1-en-1-yl)benzene (3da)[6]

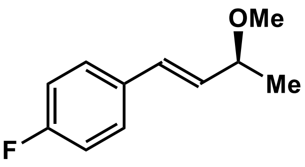

Method A (2 h), colorless oil, 91% yield, 95:5 *er*, >20:1 *rr*, $[\alpha]^{28}_D$ = +66.92 (c 0.52, CH_2Cl_2). **^{1}H NMR** (400 MHz, $CDCl_3$) δ 7.41 – 7.30 (m, 2H), 7.06 – 6.95 (m, 2H), 6.49 (d, *J* = 16.0 Hz, 1H), 6.01 (dd, *J* = 15.9, 7.6 Hz, 1H), 3.92 – 3.83 (m, 1H), 3.32 (s, 3H), 1.33 (d, *J* = 6.4 Hz, 3H). **^{13}C NMR** (101 MHz, $CDCl_3$) δ 162.5 (d, *J* = 247.5 Hz), 133.0 (d, *J* = 3.0 Hz), 131.4 (d, *J* = 2.0 Hz), 130.3, 128.1 (d, *J* = 8.1 Hz), 115.6 (d, *J* = 21.2 Hz), 78.2, 56.2, 21.6. **^{19}F NMR** (376 MHz, $CDCl_3$) δ -114.5. **HPLC**: chiral stationary column OJ-H, mobile phase hexane/^{i}PrOH = 99/1, 1.0 mL/min, 254 nm, 30 °C, t_R (minor) = 7.0 min, t_R (major) = 7.5 min.

(*S,E*)-1-chloro-4-(3-methoxybut-1-en-1-yl)benzene (3ea)[5]

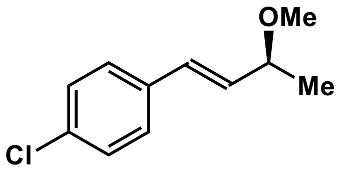

Method A (10 °C 1.5 h), colorless oil, 66% yield, 92:8 *er*, >20:1 *rr*, $[\alpha]^{28}_D$ = +59.22 (c 0.39, CH_2Cl_2). **^{1}H NMR** (400 MHz, $CDCl_3$) δ 7.27 – 7.17 (m, 4H), 6.41 (d, *J* = 16.0 Hz, 1H), 6.00 (dd, *J* = 16.0, 7.5 Hz, 1H), 3.86 – 3.77 (m, 1H), 3.25 (s, 3H), 1.25 (d, *J* = 6.4 Hz, 3H). **^{13}C NMR** (101 MHz, $CDCl_3$) δ 135.4, 133.4, 132.4, 130.2, 128.9, 127.8, 78.1, 56.3, 21.5. **HPLC**: chiral stationary column OJ-H, mobile phase hexane/^{i}PrOH = 99.8/0.2, 1.5 mL/min, 254 nm, 30 °C, t_R (minor) = 6.8 min, t_R (major) = 7.1 min.

(*S,E*)-4-(3-methoxybut-1-en-1-yl)-*N,N*-dimethylaniline (3fa)

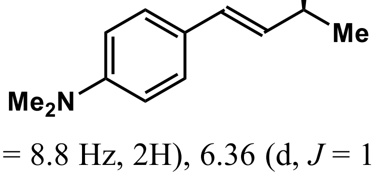

Method A (60 °C, 3 h), colorless oil, 73% yield, 81:19 *er*, >20:1 *rr*, $[\alpha]^{28}_D$ = +75.05 (c 0.49, CH_2Cl_2). **^{1}H NMR** (400 MHz, $CDCl_3$) δ 7.21 (d, *J* = 8.8 Hz, 2H), 6.61 (d, *J* = 8.8 Hz, 2H), 6.36 (d, *J* = 15.9 Hz, 1H), 5.79 (dd, *J* = 15.9, 7.9 Hz, 1H), 3.82 – 3.73 (m, 1H), 3.22 (s, 3H), 2.88 (s, 6H), 1.24 (d, *J* = 6.3 Hz, 3H). **^{13}C NMR** (101 MHz, $CDCl_3$) δ 150.3, 131.8, 127.7, 127.6, 127.3, 112.7, 78.7, 56.0, 40.7, 21.9. **IR** (neat):

2972, 2926, 2816, 1609, 1353, 1109, 967, 807 cm^{-1}. **HRMS** calculated for $C_{13}H_{20}NO$ $[M+H]^+$ 206.1545, found 206.1540. **HPLC**: chiral stationary column OJ-H, mobile phase hexane/^{i}PrOH = 90/10, 1.0 mL/min, 254 nm, 30 °C, t_R (minor) = 12.1 min, t_R (major) = 13.9 min.

(*S,E*)-1-(3-methoxybut-1-en-1-yl)-3-methylbenzene (3ga)

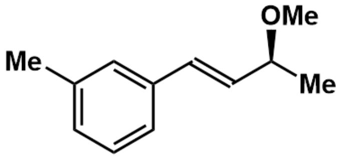

Method A (5 h), colorless oil, 92% yield, 96:4 *er*, >20:1 *rr*, $[\alpha]^{28}_D$ = +87.64 (c 0.45, CH_2Cl_2). **^{1}H NMR** (400 MHz, $CDCl_3$) δ 7.21 (d, *J* = 6.6 Hz, 3H), 7.08 – 7.04 (m, 1H), 6.51 (d, *J* = 15.9 Hz, 1H), 6.08 (dd, *J* = 15.9, 7.6 Hz, 1H), 3.93 – 3.84 (m, 1H), 3.32 (s, 3H), 2.35 (s, 3H), 1.33 (d, *J* = 6.3 Hz, 3H). **^{13}C NMR** (101 MHz, $CDCl_3$) δ 138.3, 136.8, 131.7, 131.5, 128.7, 128.6, 127.4, 123.8, 78.3, 56.2, 21.7, 21.6. **IR** (neat): 3024, 2975, 2926, 1448, 1082, 967, 777, 693 cm^{-1}. **HRMS** calculated for $C_{12}H_{17}O$ $[M+H]^+$ 177.1279, found 177.1273. **HPLC**: chiral stationary column OJ-H, mobile phase hexane/^{i}PrOH = 99/1, 1.0 mL/min, 254 nm, 30 °C, t_R (minor) = 6.3 min, t_R (major) = 6.7 min.

(*S,E*)-1-methoxy-2-(3-methoxybut-1-en-1-yl)benzene (3ha)[5]

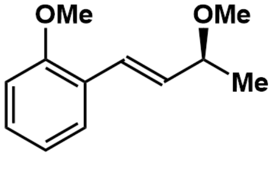

Method A (5 h), colorless oil, 94% yield, 95:5 *er*, >20:1 *rr*, $[\alpha]^{28}_D$ = +66.67 (c 0.51, CH_2Cl_2). **^{1}H NMR** (400 MHz, $CDCl_3$) δ 7.42 – 7.35 (m, 1H), 7.19 – 7.10 (m, 1H), 6.88 – 6.73 (m, 3H), 6.02 (dd, *J* = 16.1, 7.8 Hz, 1H), 3.86 – 3.78 (m, 1H), 3.77 (s, 3H), 3.24 (s, 3H), 1.25 (d, *J* = 6.3 Hz, 3H). **^{13}C NMR** (101 MHz, $CDCl_3$) δ 156.9, 132.1, 128.8, 127.0, 126.4, 125.8, 120.8, 111.1, 78.8, 56.1, 55.6, 21.8. **HPLC**: chiral stationary column AD-H, mobile phase hexane/^{i}PrOH = 99.5/0.5, 1.0 mL/min, 254 nm, 30 °C, t_R (minor) = 7.4 min, t_R (major) = 9.6 min.

(*S,E*)-2-(3-methoxybut-1-en-1-yl)furan (3ia)

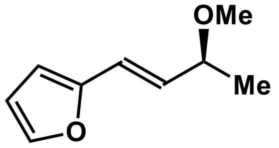

Method A (2 h), light yellow oil, 92% yield, 95:5 *er*, >20:1 *rr*, $[\alpha]^{28}_{D}$ = +93.47 (c 0.51, CH_2Cl_2). **^{1}H NMR** (400 MHz, $CDCl_3$) δ 7.38 – 7.30 (m, 1H), 6.41 – 6.31 (m, 2H), 6.24 (d, *J* = 3.2 Hz, 1H), 6.04 (dd, *J* = 15.9, 7.4 Hz, 1H), 3.89 – 3.81 (m, 1H), 3.31 (s, 3H), 1.31 (d, *J* = 6.4 Hz, 3H). **^{13}C NMR** (101 MHz, $CDCl_3$) δ 152.5, 142.1, 130.3, 119.7, 111.4, 108.1, 77.9, 56.3, 21.6. **IR** (neat): 2969, 2926, 2853, 1247, 1013, 962, 732 cm^{-1}. **HRMS** calculated for $C_9H_{13}O_2$ $[M+H]^+$ 153.0916, found 153.0910. **HPLC**: chiral stationary column OJ-H, mobile phase hexane/^{i}PrOH = 99/1, 1.0 mL/min, 254 nm, 30 °C, t_R (minor) = 6.6 min, t_R (major) = 7.4 min.

(*S,E*)-2-(3-methoxybut-1-en-1-yl)thiophene (3ja)

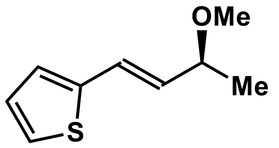

Method A (2 h), light yellow oil, 65% yield, 93:7 *er*, >20:1 *rr*, $[\alpha]^{28}_{D}$ = +38.37 (c 0.32, CH_2Cl_2). **^{1}H NMR** (400 MHz, $CDCl_3$) δ 7.20 – 7.12 (m, 1H), 7.00 – 6.92 (m, 2H), 6.66 (d, *J* = 15.8 Hz, 1H), 5.94 (dd, *J* = 15.8, 7.5 Hz, 1H), 3.90 – 3.80 (m, 1H), 3.32 (s, 3H), 1.32 (d, *J* = 6.4 Hz, 3H). **^{13}C NMR** (101 MHz, $CDCl_3$) δ 142.0, 131.3, 127.5, 125.9, 124.6, 124.5, 78.0, 56.3, 21.5. **IR** (neat): 2974, 2926, 2853, 1458, 1109, 957, 696 cm^{-1}. **HRMS** calculated for $C_9H_{13}OS$ $[M+H]^+$ 169.0687, found 169.0681. **HPLC**: chiral stationary column OJ-H, mobile phase hexane/^{i}PrOH = 99/1, 1.0 mL/min, 254 nm, 30 °C, t_R (minor) = 6.7 min, t_R (major) = 7.4 min.

(*S,E*)-4-(((4-cyclohexylbut-3-en-2-yl)oxy)methyl)-1,1'-biphenyl (3ka)

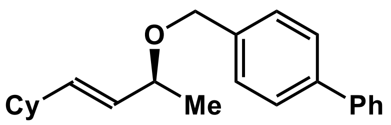

Method B (1 h), colorless oil, 31% yield, 88:12 *er*, >20:1 *rr*, $[\alpha]^{28}_{D}$ = +32.73 (c 0.22, CH_2Cl_2). **^{1}H NMR** (400 MHz, $CDCl_3$) δ 7.62 – 7.55 (m, 4H), 7.47 – 7.38 (m, 4H), 7.37 – 7.31 (m, 1H), 5.59 (dd, *J* = 15.6, 6.6 Hz, 1H), 5.35 (dd, *J* = 15.6, 6.6 Hz, 1H), 4.59 (d, *J* = 12.0 Hz, 1H), 4.41 (d, *J* = 12.0 Hz, 1H), 3.94 – 3.85 (m, 1H), 2.05 – 1.95 (m, 1H), 1.82 – 1.70 (m, 5H), 1.35 – 1.07 (m, 8H). **^{13}C NMR** (101 MHz, $CDCl_3$) δ 141.3, 140.5, 139.5, 138.3, 129.3, 128.9, 128.4, 127.4, 127.3, 76.3, 69.5, 40.5, 33.2, 33.1, 26.4, 26.2, 22.1. **IR** (neat): 3028, 2971, 2924, 2851, 1448, 1074, 971, 823,

759, 697 cm^{-1}. **HRMS** calculated for $C_{23}H_{29}O$ $[M+H]^+$ 321.2218, found 321.2214. **HPLC**: chiral stationary column OJ-H, mobile phase hexane/^{i}PrOH = 90/10, 1.0 mL/min, 254 nm, 30 °C, t_R (major) = 5.3 min, t_R (minor) = 5.8 min.

(*S*)-4-((but-3-en-2-yloxy)methyl)-1,1'-biphenyl (3la)[7]

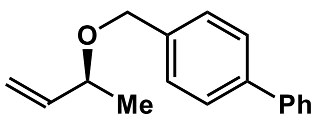

Method B (butadiene (10 eq.) is in hexane (20%), 20 min), colorless oil, 48% yield, 80:20 *er*, >20:1 *rr*, $[\alpha]^{28}_D$ = +11.74 (c 0.46, CH_2Cl_2). **^{1}H NMR** (400 MHz, $CDCl_3$) δ 7.59 (t, *J* = 7.3 Hz, 4H), 7.49 – 7.40 (m, 4H), 7.35 (t, *J* = 7.3 Hz, 1H), 5.89 – 5.77 (m, 1H), 5.29 – 5.18 (m, 2H), 4.63 (d, *J* = 11.9 Hz, 1H), 4.45 (d, *J* = 11.9 Hz, 1H), 4.03 – 3.92 (m, 1H), 1.33 (d, *J* = 6.4 Hz, 3H). **^{13}C NMR** (101 MHz, $CDCl_3$) δ 141.2, 140.6, 140.4, 138.0, 128.9, 128.3, 127.4, 127.31, 127.27, 116.4, 76.5, 69.9, 21.6. **HPLC**: chiral stationary column OJ-H, mobile phase hexane/^{i}PrOH = 98/2, 1.0 mL/min, 254 nm, 30 °C, t_R (major) = 10.8 min, t_R (minor) = 11.5 min.

(S)-(3-methoxybut-1-en-2-yl)benzene (3ma)[8]

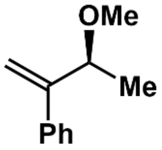

Method A (30 °C, 3 h), colorless oil, 77% yield, 62:38 *er*, >20:1 *rr*, $[\alpha]^{15}_D$ = +10.00 (c 0.16, CH_2Cl_2). **^{1}H NMR** (400 MHz, $CDCl_3$) δ 7.37 – 7.31 (m, 2H), 7.29 – 7.16 (m, 3H), 5.29 (s, 1H), 5.21 (s, 1H), 4.12 (q, *J* = 6.2 Hz, 1H), 3.31 (s, 3H), 1.20 (d, *J* = 6.5 Hz, 3H). **^{13}C NMR** (101 MHz, $CDCl_3$) δ 149.5, 140.1, 128.4, 127.7, 127.1, 114.2, 80.2, 56.4, 21.3. **HPLC**: chiral stationary column OJ-H, mobile phase hexane/^{i}PrOH = 90/10, 1.0 mL/min, 254 nm, 30 °C, t_R (minor) = 5.1 min, t_R (major) = 5.5 min.

(*S*,*E*)-(3-ethoxybut-1-en-1-yl)benzene (3ab)[5]

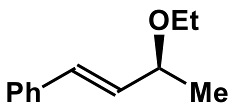

Method A (4 h), colorless oil, 79% yield, 95:5 *er*, >20:1 *rr*, $[\alpha]^{28}_D$ = +77.08 (c 0.48, CH_2Cl_2). **^{1}H NMR** (400 MHz, $CDCl_3$) δ 7.36 – 7.28 (m, 2H), 7.28 – 7.20 (m, 2H), 7.20 – 7.12 (m, 1H), 6.44 (d, *J* = 16.0 Hz, 1H), 6.05 (dd, *J* = 16.0, 7.6 Hz, 1H), 3.93 (m, 1H), 3.55 – 3.44 (m, 1H), 3.39 – 3.29 (m, 1H), 1.26 (d, *J* = 6.4 Hz, 3H), 1.14 (t, *J* = 7.0 Hz, 3H). **^{13}C NMR** (101 MHz, $CDCl_3$) δ 136.9,

132.3, 131.0, 128.7, 127.7, 126.6, 76.5, 63.8, 21.9, 15.6. **HPLC**: chiral stationary column OJ-H, mobile phase hexane/^{i}PrOH = 99/1, 1.0 mL/min, 254 nm, 30 °C, t_R (minor) = 5.6 min, t_R (major) = 6.6 min.

(*S*,*E*)-(3-(benzyloxy)but-1-en-1-yl)benzene (3ac)[9]

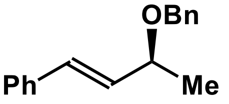

Method A (3 h), colorless oil, 90% yield, 92:8 *er*, >20:1 *rr*, $[\alpha]^{32}_D$ = +53.71 (c 0.35, toluene). **^{1}H NMR** (400 MHz, $CDCl_3$) δ 7.46 – 7.39 (m, 2H), 7.39 – 7.31 (m, 6H), 7.30 – 7.22 (m, 2H), 6.55 (d, *J* = 16.0 Hz, 1H), 6.17 (dd, *J* = 16.0, 7.7 Hz, 1H), 4.62 (d, *J* = 12.0 Hz, 1H), 4.45 (d, *J* = 12.0 Hz, 1H), 4.16 – 4.07 (m, 1H), 1.39 (d, *J* = 6.4 Hz, 3H). **^{13}C NMR** (101 MHz, $CDCl_3$) δ 139.0, 136.8, 131.9, 131.6, 128.8, 128.6, 127.9, 127.6, 126.7, 76.0, 70.2, 22.0. **HPLC**: chiral stationary column OJ-H, mobile phase hexane/^{i}PrOH = 90/10, 1.0 mL/min, 254 nm, 30 °C, t_R (minor) = 6.8 min, t_R (major) = 7.9 min.

(*S*,*E*)-(3-((4-phenylbut-3-en-2-yl)oxy)propyl)benzene (3ad)[10]

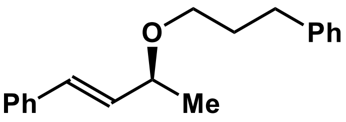

Method A (4 h), colorless oil, 75% yield, 94:6 *er*, >20:1 *rr*, $[\alpha]^{28}_D$ = +87.01 (c 0.49, CH_2Cl_2). **^{1}H NMR** (400 MHz, $CDCl_3$) δ 7.33 – 7.27 (m, 2H), 7.23 (t, *J* = 7.5 Hz, 2H), 7.20 – 7.05 (m, 6H), 6.42 (d, *J* = 16.0 Hz, 1H), 6.04 (dd, *J* = 16.0, 7.5 Hz, 1H), 3.94 – 3.85 (m, 1H), 3.49 – 3.39 (m, 1H), 3.34 – 3.23 (m, 1H), 2.69 – 2.54 (m, 2H), 1.88 – 1.77 (m, 2H), 1.26 (d, *J* = 6.4 Hz, 3H). **^{13}C NMR** (101 MHz, $CDCl_3$) δ 142.2, 136.9, 132.3, 131.0, 128.72, 128.65, 128.4, 127.7, 126.6, 125.9, 76.7, 67.6, 32.6, 31. 7, 21.9. **HPLC**: chiral stationary column OJ-H, mobile phase hexane/^{i}PrOH = 90/10, 1.0 mL/min, 254 nm, 30 °C, t_R (minor) = 6.5 min, t_R (major) = 7.7 min.

(*S*,*E*)-(3-(3-chloropropoxy)but-1-en-1-yl)benzene (3ae)

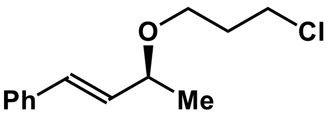

Method A (8 h), colorless oil, 92% yield, 92:8 *er*, >20:1 *rr*, $[\alpha]^{28}_D$ = +92.44 (c 0.23, CH_2Cl_2). **^{1}H NMR** (400 MHz, $CDCl_3$) δ 7.32 (d, *J* = 7.3 Hz, 2H), 7.25 (t, *J* = 7.5 Hz, 2H), 7.21 – 7.14 (m, 1H), 6.45 (d, *J* = 16.0 Hz, 1H), 6.03 (dd, *J* = 16.0, 7.5 Hz, 1H), 3.98 – 3.89 (m, 1H), 3.64 – 3.53

(m, 3H), 3.46 – 3.38 (m, 1H), 2.00 – 1.90 (m, 2H), 1.26 (d, *J* = 6.4 Hz, 3H). **^{13}C NMR** (101 MHz, $CDCl_3$) δ 136.8, 131.9, 131.2, 128.8, 127.8, 126.6, 77.0, 64.8, 42.3, 33.1, 21.8. **IR** (neat): 2026, 2972, 2927, 2866, 1448, 1099, 966, 749, 694 cm^{-1}. **HRMS** calculated for $C_{13}H_{18}ClO$ $[M+H]^+$ 225.1046, found 225.1043. **HPLC**: chiral stationary column AS-H, mobile phase hexane/^{i}PrOH = 99/1, 1.0 mL/min, 254 nm, 30 °C, t_R (minor) = 4.0 min, t_R (major) = 5.7 min.

(*S,E*)-trimethyl(3-((4-phenylbut-3-en-2-yl)oxy)propyl)silane (3af)

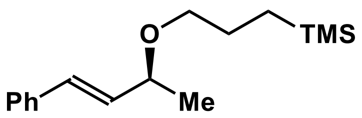

Method A (4 h), colorless oil, 60% yield, 96:4 *er*, >20:1 *rr*, $[\alpha]^{28}_D$ = +65.53 (c 0.47, CH_2Cl_2). **^{1}H NMR** (400 MHz, $CDCl_3$) δ 7.46 – 7.38 (m, 2H), 7.33 (t, *J* = 7.5 Hz, 2H), 7.29 – 7.21 (m, 1H), 6.53 (d, *J* = 16.0 Hz, 1H), 6.14 (dd, *J* = 15.9, 7.5 Hz, 1H), 4.06 – 3.95 (m, 1H), 3.52 – 3.43 (m, 1H), 3.37 – 3.27 (m, 1H), 1.65 – 1.55 (m, 2H), 1.35 (d, *J* = 6.4 Hz, 3H), 0.53 – 0.44 (m, 2H), 0.00 (s, 9H). **^{13}C NMR** (101 MHz, $CDCl_3$) δ 136.9, 132.4, 130.9, 128.7, 127.7, 126.6, 76.6, 71.7, 24.6, 21.9, 12.8, -1.55. **IR** (neat): 3027, 2952, 2928, 2870, 1093, 966, 858, 748, 692 cm^{-1}. **HRMS** calculated for $C_{16}H_{27}OSi$ $[M+H]^+$ 263.1831, found 263.1826. **HPLC**: chiral stationary column OJ-H, mobile phase hexane/^{i}PrOH = 99.8/0.2, 1.5 mL/min, 254 nm, 30 °C, t_R (minor) = 3.2 min, t_R (major) = 3.4 min.

((*S,E*)-3-(cinnamyloxy)but-1-en-1-yl)benzene (3ag)

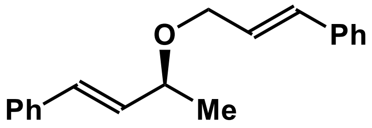

Method A (5 h), colorless oil, 72% yield, 95:5 *er*, >20:1 *rr*, $[\alpha]^{28}_D$ = +100.00 (c 0.45, CH_2Cl_2). **^{1}H NMR** (400 MHz, $CDCl_3$) δ 7.35 – 7.28 (m, 4H), 7.28 – 7.20 (m, 4H), 7.19 – 7.12 (m, 2H), 5.56 – 6.43 (m, 2H), 6.28 – 6.18 (m, 1H), 6.07 (dd, *J* = 16.0, 7.7 Hz, 1H), 4.19 – 4.12 (m, 1H), 4.09 – 3.96 (m, 2H), 1.31 (d, *J* = 6.4 Hz, 3H). **^{13}C NMR** (101 MHz, $CDCl_3$) δ 137.0, 136.8, 132.3, 131.8, 131.6, 128.8, 128.7, 127.9, 127.7, 126.6, 76.1, 69.0, 21.9. **IR** (neat): 3026, 2974, 2927, 1072, 966, 748, 692 cm^{-1}. **HRMS** calculated for $C_{19}H_{21}O$ $[M+H]^+$ 265.1592, found 265.1588. **HPLC**: chiral stationary column OJ-H, mobile phase hexane/^{i}PrOH = 90/10, 1 mL/min, 254 nm, 30 °C, t_R (minor) = 12.0 min, t_R (major) = 13.6 min.

(*S,E*)-1-fluoro-4-(((4-phenylbut-3-en-2-yl)oxy)methyl)benzene (3ah)

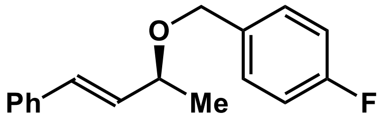

Method A (4 h), colorless oil, 95% yield, 91:9 *er*, >20:1 *rr*, $[\alpha]^{28}_{D}$ = +96.30 (c 0.54, CH_2Cl_2). **^{1}H NMR** (400 MHz, $CDCl_3$) δ 7.37 – 7.30 (m, 2H), 7.29 – 7.21 (m, 4H), 7.21 – 7.14 (m, 1H), 6.95 (t, *J* = 8.7 Hz, 2H), 6.47 (d, *J* = 16.0 Hz, 1H), 6.08 (dd, *J* = 16.0, 7.7 Hz, 1H), 4.49 (d, *J* = 11.8 Hz, 1H), 4.32 (d, *J* = 11.8 Hz, 1H), 4.06 – 3.96 (m, 1H), 1.30 (d, *J* = 6.4 Hz, 3H). **^{13}C NMR** (101 MHz, $CDCl_3$) δ 162.43 (d, *J* = 245.4 Hz), 136.8, 134.7 (d, *J* = 3.0 Hz), 131.7, 129.6 (d, *J* = 8.1 Hz), 128.8, 127.9, 126.7, 115.4 (d, *J* = 21.2 Hz), 76.2, 69.6, 21.9. **^{19}F NMR** (376 MHz, $CDCl_3$) δ -115.2. **IR** (neat): 3027, 2975, 2928, 2860, 1509, 1222, 1078, 969, 823, 749, 693 cm^{-1}. **HRMS** calculated for $C_{17}H_{18}FO$ $[M+H]^+$ 257.1342, found 257.1333. **HPLC**: chiral stationary column AD-H, mobile phase hexane/^{i}PrOH = 90/10, 1.0 mL/min, 254 nm, 30 °C, t_R (minor) = 4.3 min, t_R (major) = 4.6 min.

(*S,E*)-1-methyl-4-(((4-phenylbut-3-en-2-yl)oxy)methyl)benzene (3ai)

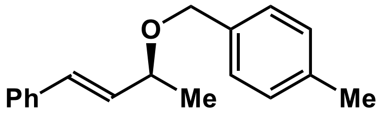

Method A (4 h), colorless oil, 93% yield, 94:6 *er*, >20:1 *rr*, $[\alpha]^{28}_{D}$ = +121.01 (c 0.40, CH_2Cl_2). **^{1}H NMR** (400 MHz, $CDCl_3$) δ 7.36 – 7.30 (m, 2H), 7.29 – 7.21 (m, 2H), 7.21 – 7.13 (m, 3H), 7.07 (d, *J* = 7.9 Hz, 2H), 6.46 (d, *J* = 16.0 Hz, 1H), 6.09 (dd, *J* = 16.0, 7.7 Hz, 1H), 4.50 (d, *J* = 11.8 Hz, 1H), 4.32 (d, *J* = 11.8 Hz, 1H), 4.06 – 3.97 (m, 1H), 2.26 (s, 3H), 1.29 (d, *J* = 6.4 Hz, 3H). **^{13}C NMR** (101 MHz, $CDCl_3$) δ 137.3, 136.9, 135.9, 132.0, 131.5, 129.2, 128.8, 128.0, 127.8, 126.7, 75.8, 70.1, 22.0, 21.4. **IR** (neat): 3025, 2974, 2926, 2860, 1493, 1078, 968, 801, 748, 693 cm^{-1}. **HRMS** calculated for $C_{18}H_{21}O$ $[M+H]^+$ 253.1592, found 253.1588. **HPLC**: chiral stationary column OJ-H, mobile phase hexane/^{i}PrOH = 90/10, 1.0 mL/min, 254 nm, 30 °C, t_R (minor) = 7.4 min, t_R (major) = 8.5 min.

(*S,E*)-1-methoxy-4-(((4-phenylbut-3-en-2-yl)oxy)methyl)benzene (3aj)

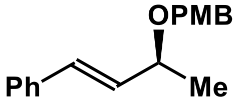

Method A (1 h), colorless oil, 78% yield, 94:6 *er*, >20:1 *rr*, $[\alpha]^{28}_{D}$ = +108.08 (c 0.52, CH_2Cl_2). **^{1}H NMR** (400 MHz, $CDCl_3$) δ 7.33

(d, J = 7.3 Hz, 2H), 7.25 (t, J = 7.5 Hz, 2H), 7.22 – 7.14 (m, 3H), 6.80 (d, J = 8.6 Hz, 2H), 6.46 (d, J = 16.0 Hz, 1H), 6.09 (dd, J = 16.0, 7.7 Hz, 1H), 4.47 (d, J = 11.5 Hz, 1H), 4.30 (d, J = 11.5 Hz, 1H), 4.01 (m, 1H), 3.72 (s, 3H), 1.29 (d, J = 6.4 Hz, 3H). **^{13}C NMR** (101 MHz, $CDCl_3$) δ 159.3, 136.9, 132.0, 131.5, 131.0, 129.5, 128.8, 127.8, 126.7, 114.0, 75.7, 69.9, 55.5, 22.0. **IR** (neat): 3026, 2972, 2929, 2861, 1512, 1246, 1070, 968, 749, 693 cm^{-1}. **HRMS** calculated for $C_{18}H_{21}O_2$ $[M+H]^+$ 269.1542, found 269.1538. **HPLC**: chiral stationary column OJ-H, mobile phase hexane/^{i}PrOH = 90/10, 1.0 mL/min, 254 nm, 30 °C, t_R (minor) = 14.0 min, t_R (major) = 14.9 min.

((*S*,*E*)-3-(((*S*)-3,7-dimethyloct-6-en-1-yl)oxy)but-1-en-1-yl)benzene (3ak)

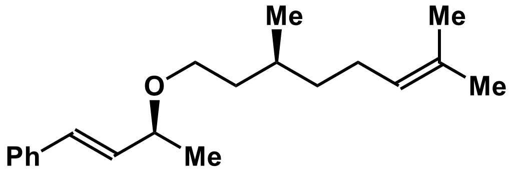

Method A (7 h), colorless oil, 73% yield, >20:1 *dr*, >20:1 *rr*; $[\alpha]^{28}_D$ = +52.27 (c 0.44, CH_2Cl_2). **^{1}H NMR** (400 MHz, $CDCl_3$) δ 7.35 – 7.29 (m, 2H), 7.24 (t, J = 7.5 Hz, 2H), 7.20 – 7.13 (m, 1H), 6.44 (d, J = 16.0 Hz, 1H), 6.04 (dd, J = 15.9, 7.5 Hz, 1H), 5.07 – 4.98 (m, 1H), 3.96 – 3.86 (m, 1H), 3.50 – 3.40 (m, 1H), 3.37 – 3.26 (m, 1H), 1.99 – 1.82 (m, 2H), 1.63 – 1.44 (m, 8H), 1.37 – 1.23 (m, 5H), 1.14 – 1.03 (m, 1H), 0.81 (d, J = 6.6 Hz, 3H). **^{13}C NMR** (101 MHz, $CDCl_3$) δ 137.0, 132.4, 131.3, 130.9, 128.7, 127.7, 126.6, 125.0, 76.6, 66.9, 37.5, 37.1, 29.8, 25.9, 25.7, 21.9, 19.8, 17.8. **IR** (neat): 3026, 2966, 2926, 2856, 1091, 967, 748, 692 cm^{-1}. **HRMS** calculated for $C_{20}H_{31}O$ $[M+H]^+$ 287.2375, found 287.2372.

(*S*,*E*)-(3-cyclopropoxybut-1-en-1-yl)benzene (3al)

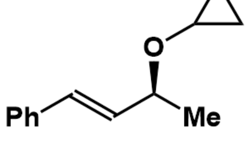

Method A (4 h), colorless oil, 88% yield, 97:3 *er*, >20:1 *rr*, $[\alpha]^{28}_D$ = +154.75 (c 0.5, CH_2Cl_2). **^{1}H NMR** (400 MHz, $CDCl_3$) δ 7.35 – 7.28 (m, 2H), 7.24 (t, J = 7.5 Hz, 2H), 7.19 – 7.12 (m, 1H), 6.49 (d, J = 15.9 Hz, 1H), 6.10 (dd, J = 15.9, 7.5 Hz, 1H), 4.11 – 4.02 (m, 1H), 3.30 – 3.22 (m, 1H), 1.24 (d, J = 6.4 Hz, 3H), 0.62 – 0.53 (m, 1H), 0.51 – 0.30 (m, 3H). **^{13}C NMR** (101 MHz, $CDCl_3$) δ 137.0, 132.1, 131.0, 128.7, 127.8, 126.6, 77.0, 51.3, 21.8, 6.3, 5.6. **IR** (neat): 3026, 2976, 2928, 2868, 1449, 1064, 966, 749, 693 cm^{-1}. **HRMS** calculated for $C_{13}H_{17}O$ $[M+H]^+$ 189.1279, found 189.1275. **HPLC**: chiral stationary column OJ-

H, mobile phase hexane/^{i}PrOH = 99/1, 1.0 mL/min, 254 nm, 30 °C, t_R (minor) = 6.4 min, t_R (major) = 7.0 min.

(*S,E*)-(3-(cyclopentyloxy)but-1-en-1-yl)benzene (3am)

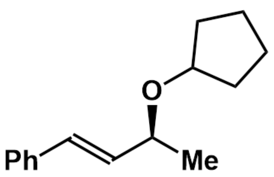

Method A (60 °C, 3 h), colorless oil, 65% yield, 91:9 *er*, >20:1 *rr*, $[\alpha]^{28}_D$ = +84.35 (c 0.46, CH_2Cl_2). **^{1}H NMR** (400 MHz, $CDCl_3$) δ 7.31 (d, *J* = 7.4 Hz, 2H), 7.24 (t, *J* = 7.5 Hz, 2H), 7.15 (t, *J* = 7.3 Hz, 1H), 6.43 (d, *J* = 16.0 Hz, 1H), 6.06 (dd, *J* = 15.9, 7.3 Hz, 1H), 4.04 – 3.90 (m, 2H), 1.72 – 1.60 (m, 4H), 1.59 – 1.49 (m, 2H), 1.46 – 1.35 (m, 2H), 1.23 (d, *J* = 6.4 Hz, 3H). **^{13}C NMR** (101 MHz, $CDCl_3$) δ 137.1, 133.0, 130.4, 128.7, 127.7, 126.6, 78.7, 74.5, 33.4, 32.3, 23.71, 23.70, 22.3. **IR** (neat): 3026, 2963, 2869, 1449, 1076, 967, 748, 692 cm^{-1}. **HRMS** calculated for $C_{15}H_{21}O$ $[M+H]^+$ 217.1592, found 217.1588. **HPLC**: chiral stationary column AD-H, mobile phase hexane/^{i}PrOH = 99/1, 1.0 mL/min, 254 nm, 30 °C, t_R (minor) = 4.0 min, t_R (major) = 4.2 min.

4. Mechanistic studies

4.1. Acid/base additives for hydroalkoxylation

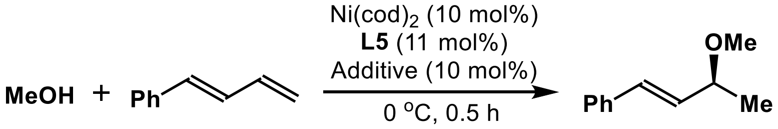

1a 2a 3aa

In a N_2-filled glovebox, (*R*, *R*)-Me-DuPhos (6.7 mg, 0.022 mmol) and 1,3-diene **2a** (78.1 mg, 0.6 mmol) were added to a 1-dram vial containing $Ni(cod)_2$ (5.6 mg, 0.02 mmol). The resulting mixture was stirred for 10 min and then additive (0.02 mmol) and alcohol **1a** (6.4 mg, 0.2 mmol) were added. The mixture was removed from the glovebox and was held at 0 °C for 0.5 h. The NMR yield were determined by ^{1}H NMR analysis of the unpurified reaction mixture.

Entry	Additive	Yield (%)
1	none	42%
2	TFA	NR
3	xylylic acid	NR
4	TsOH	NR
5	Et_3N	42%
6	NaOH	42%
7	^{t}BuONa	14%

4.2. Kinetic studies

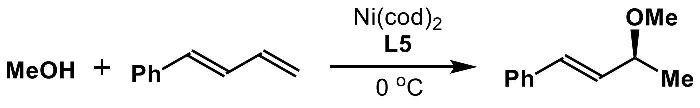

The kinetic profile of the reaction was determined using the variable time normalization analysis (VTNA) method described by Burés.[11] Rates were monitored using GC-FID analysis with 1,3,5-trimethoxybenzene as a standard. Catalyst concentration was assumed to remain constant over the duration of the reaction. Integrals were calculated using the trapezoid rule approximation:

$$f(t_n) = \int_0^{t_n} [X]^x dt \approx \sum_{i=1}^{n} \left(\frac{[X]_i + [X]_{i-1}}{2}\right)^2 (t_i - t_{i-1})$$

Same excess experiment

Representative procedure: In a N_2-filled glovebox, (*R*, *R*)-Me-DuPhos (6.7 mg, 0.022 mmol) and 1,3-diene **2a** (78.1 mg, 0.6 mmol; or 88.5 mg, 0.68 mmol) were added to a 1-dram vial containing Ni(cod)$_2$ (5.6 mg, 0.02 mmol). The resulting mixture was stirred for 10 min and then 1,3,5-trimethoxybenzene and alcohol **1a** (6.4 mg, 0.2 mmol; 9.0 mg, 0.28 mmol) were added. The mixture was removed from the glovebox and was

held at 0 °C. Aliquots (1 μL) were taken and quenched in 1 mL of EtOAc. The amount of **3aa** was monitored by GC-FID analysis. A good graphical overlay indicates that there is no catalyst deactivation or product inhibition.

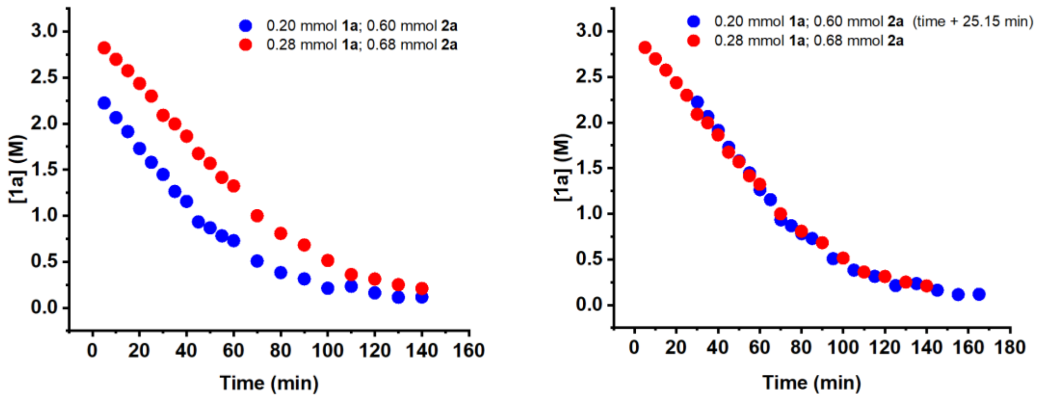

Figure S1. "Same excess" experiment graphs. Overlay of the reaction profiles was observed after time adjustment (blue points; time + 25.15 min).

Determination of the reaction order in alcohol (1):

Representative procedure: In a N_2-filled glovebox, (*R*, *R*)-Me-DuPhos (6.7 mg, 0.022 mmol) and 1,3-diene **2a** (78.1 mg, 0.6 mmol) were added to a 1-dram vial containing $Ni(cod)_2$ (5.6 mg, 0.02 mmol). The resulting mixture was stirred for 10 min and then 1,3,5-trimethoxybenzene and alcohol **1a** (3.2-16 mg, 0.1-0.5 mmol) were added. The mixture was removed from the glovebox and was held at 0 °C. Aliquots (1 μL) were taken and quenched in 1 mL of EtOAc. The amount of **3aa** was monitored by GC-FID analysis. By visual analysis, it is concluded that the reaction order depends on the concentration of alcohol. Fractional order was observed when using lower concentration of **1a**. Inverse order was observed when using higher concentration.

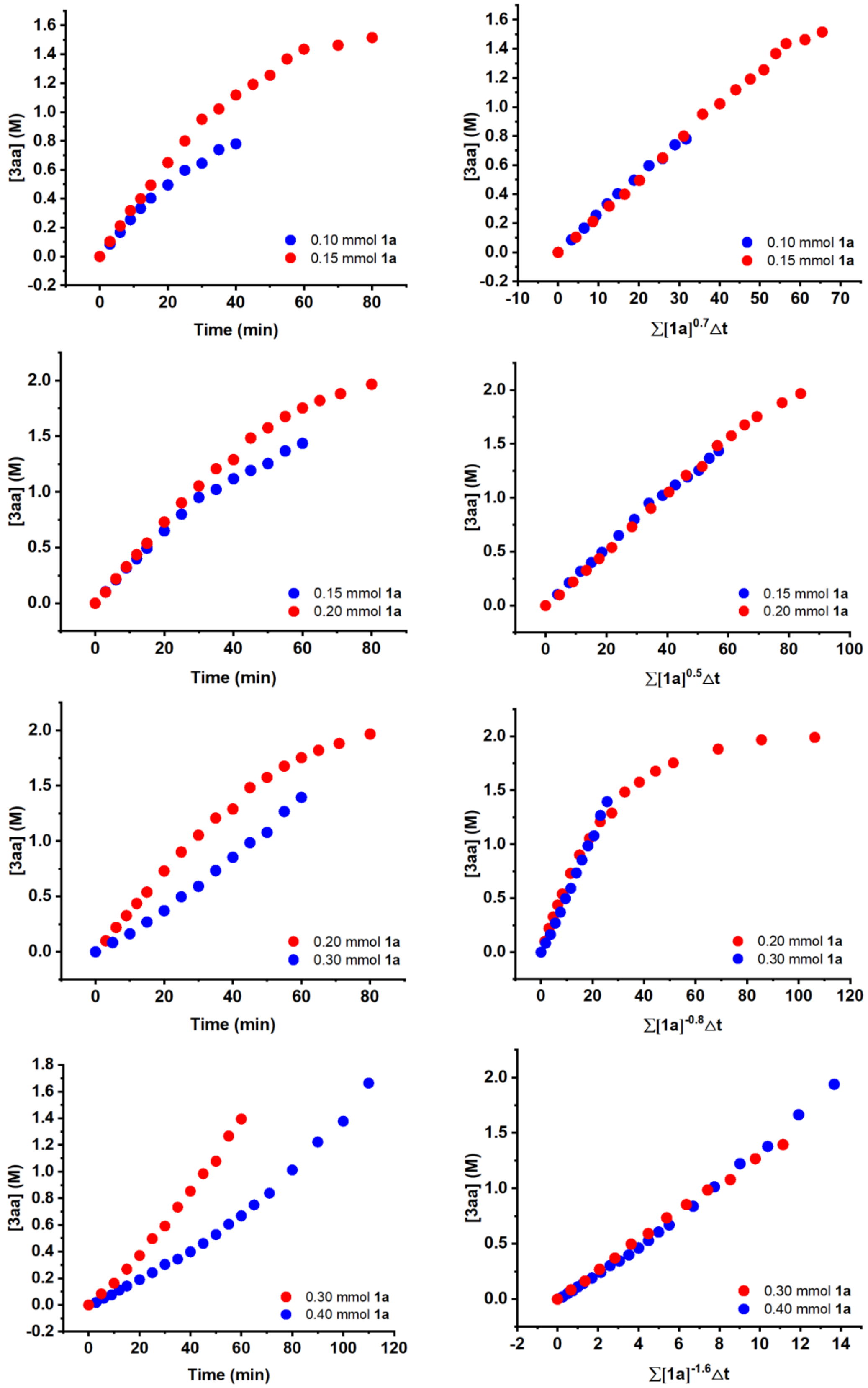
[3aa] (M)
Time (min)
0.10 mmol 1a
0.15 mmol 1a
∑[1a]^0.7∆t
0.15 mmol 1a
0.20 mmol 1a
∑[1a]^0.5∆t
0.20 mmol 1a
0.30 mmol 1a
∑[1a]^-0.8∆t
0.30 mmol 1a
0.40 mmol 1a
∑[1a]^-1.6∆t

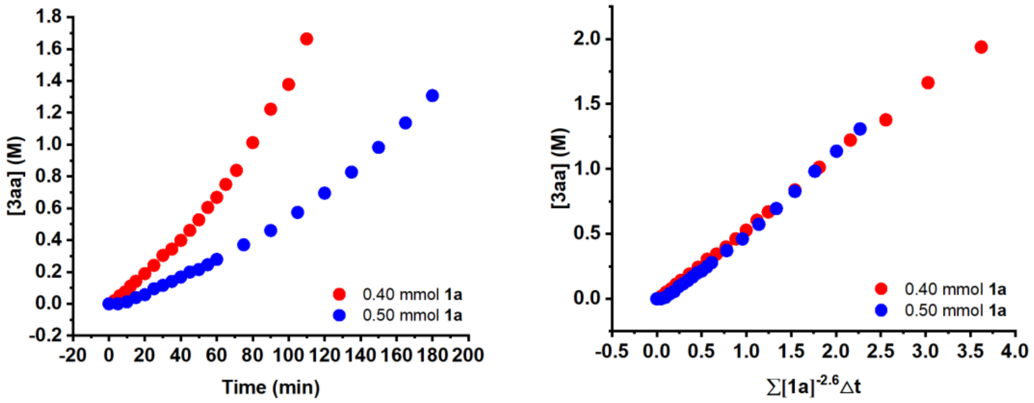

Figure S2. VTNA graphs for **1a**. Fractional order was observed when using lower concentration of **1a**. Inverse order was observed when using higher concentration.

Determination of the reaction order in diene (2)

Representative procedure: In a N_2-filled glovebox, (*R*, *R*)-Me-DuPhos (6.7 mg, 0.022 mmol) and 1,3-diene **2a** (52.1-104.2 mg, 0.4-0.8 mmol) was added to a 1-dram vial containing Ni(cod)$_2$ (5.6 mg, 0.02 mmol). The resulting mixture was stirred for 10 min and then 1,3,5-trimethoxybenzene and alcohol **1a** (6.4 mg, 0.2 mmol) were added. The mixture was removed from the glovebox and was held at 0 ºC. Aliquots (1 μL) were taken and quenched in 1 mL of EtOAc. The amount of **3aa** was monitored by GC-FID analysis. By visual analysis, it is concluded that the reaction rate is zero order with respect to diene (**2**).

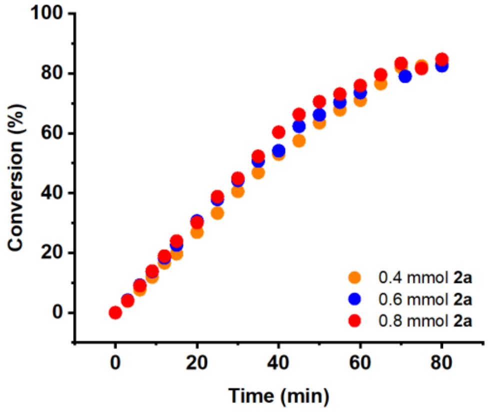

Figure S3. VTNA graphs for **2a**. Conversion instead of concentration was used because diene is used as the solvent. Overlay of the reaction profiles when using zero order analysis indicates that the reaction is zero order with respect to diene (**2**).

Determination of the reaction order in catalyst

Representative procedure: In a N_2-filled glovebox, (*R*, *R*)-Me-DuPhos (6.7 mg, 0.022 mmol; or 10.1 mg, 0.033 mmol) and 1,3-diene **2a** (78.1 mg, 0.6 mmol) were added to a 1-dram vial containing Ni(cod)$_2$ (5.6 mg, 0.02 mmol; or 8.4 mg, 0.03 mmol). The resulting mixture was stirred for 10 min and then 1,3,5-trimethoxybenzene and alcohol **1a** (6.4 mg, 0.2 mmol) were added. The mixture was removed from the glovebox and was held at 0 °C. Aliquots (1 μL) were taken and quenched in 1 mL of EtOAc. The amount of **3aa** was monitored by GC-FID analysis. By visual analysis, it is concluded that the reaction rate is first order with respect to catalyst.

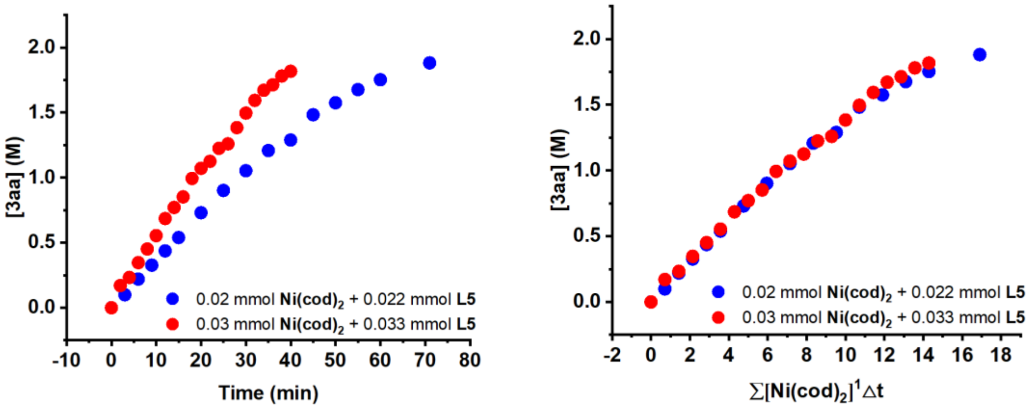

Figure S4. VTNA graphs for **Ni-catalyst**. Overlay of the reaction profiles when using first order analysis indicates that the reaction is first order with respect to catalyst.

4.3. NMR Monitoring

In a N_2-filled glovebox, (*R*, *R*)-Me-DuPhos (13.5 mg, 0.044 mmol) and 1,3-diene **2a** (418.4 mg, 3.214 mmol) were added to a 1-dram vial containing Ni(cod)$_2$ (11.2 mg, 0.04 mmol). The resulting mixture was stirred for 10 min and transferred to an NMR tube. ^{31}P NMR of this mixture were immediately recorded. Two singlets along with a broad signal were observed (**A**). The signal at 62.2 ppm corresponds to the heteroleptic complex (cod)Ni(**L5**), while the signal at 67.5 ppm corresponds to the homoleptic complex Ni(**L5**)$_2$. The broad signal is attributed to (**2a**)Ni(**L5**). The tube mentioned-above was brought back into the glovebox, and neat MeOH **1a** (51.4 mg, 1.607 mmol) or **3aa** (9.7 mg, 0.06 mmol) was added to the tube. ^{31}P NMR of these mixture were

immediately recorded. When **1a** was subjected to a mixture of Ni(cod)$_2$ and **L5** in diene **2a,** a new Ni complex with non-equivalent phosphine resonances [a pair of doublet signals (δ = 68.8 ppm, J_{P-P} = 59.3 Hz; δ = 64.6 ppm, J_{P-P} = 59.3 Hz)] was observed, and this new species is attributed to intermediate **III** (**B**). When product **3aa** was subjected to a mixture of Ni(cod)$_2$ and **L5** in diene **2a**, the ^{31}P NMR spectrum is same with just Ni(cod)$_2$ and **L5** in diene **2a** (**C**), and this excludes the intermediate species where product binding to Ni-catalyst as the resting state. Moreover, a mixture of Ni(cod)$_2$ and **L5** in *d*-methanol (0.5 mL) with addition of methanol was also monitored, and there is also no new Ni-complex species observed. On basis of these observations and our kinetic studies, we postulate intermediate **III** as the resting state. We assigned ^{31}P NMR peaks of complexes (cod)Ni(**L5**) and Ni(**L5**)$_2$ on the basis of those reported in the literature.[12] There is no ^{1}H NMR analysis because the experiment was conducted using the diene as the solvent.

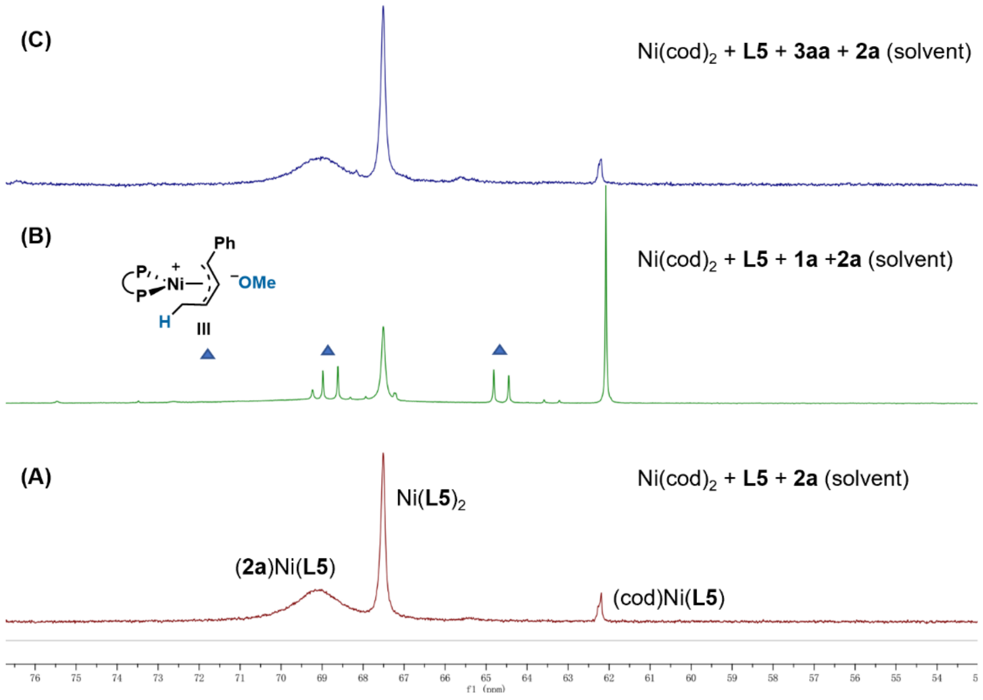

4.4. Electrospray MS (ESI-MS) analysis

In a N$_2$-filled glovebox, (*R*, *R*)-Me-DuPhos (6.7 mg, 0.022 mmol) and 1,3-diene **2f** (104.0 mg, 0.6 mmol) were added to a 1-dram vial containing Ni(cod)$_2$ (5.6 mg, 0.02 mmol). The resulting mixture was stirred for 10 min at 60 °C and then **1a (**6.4 mg, 0.2

mmol) was added with stirring for another 10 min. The catalytic solution was immediately subjected to electrospray MS (ESI-MS) analysis. **HRMS** calculated for $C_{30}H_{44}NNiP_2^+$ 538.2297, found 538.2289.

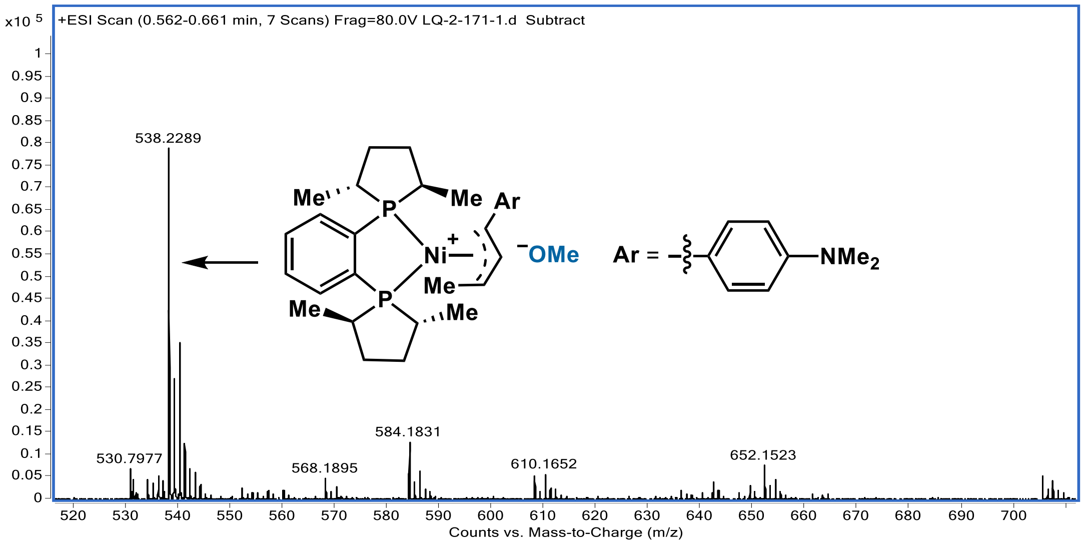

4.5. Initial rate *KIE* study

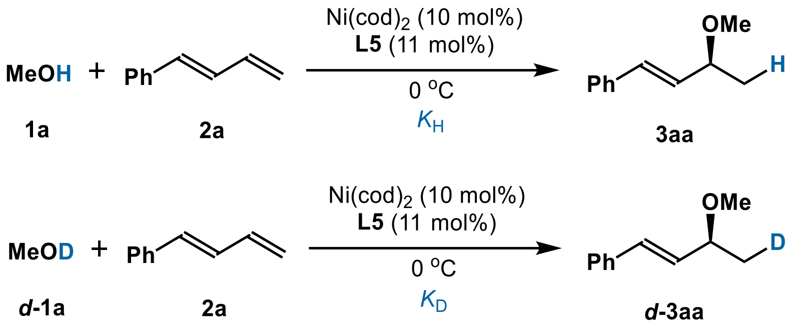

In a N_2-filled glovebox, (*R*, *R*)-Me-DuPhos (6.7 mg, 0.022 mmol) and 1,3-diene **2a** (78.1 mg, 0.6 mmol) was added to a 1-dram vial containing Ni(cod)$_2$ (5.6 mg, 0.02 mmol). The resulting mixture was stirred for 10 min and then **1a (**6.4 mg, 0.2 mmol**)** or ***d*-1a** (6.6 mg, 0.2 mmol) were added. The mixture was removed from the glovebox and was held at 0 °C. Aliquots (1 μL) were taken every 3 minutes and quenched in 1 mL of EtOAc. The amount of **3aa** and ***d*-3aa** were monitored by GC-FID analysis. The *KIE* was determined to be 1.1 using the equation: $KIE = k_H/k_D$.

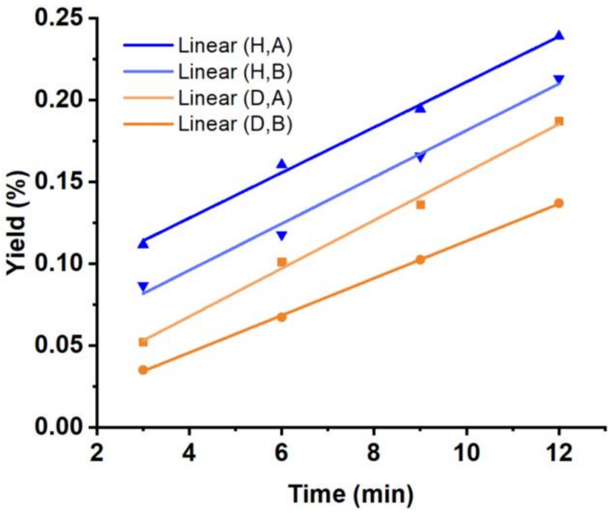

Trial	A	B	A	B	H average k_{obs}	D average k_{obs}
Alcohol	H	H	D	D	--	--
k_{obs} (%/min)	0.0139	0.0142	0.0147	0.0114	0.0141	0.0131
R^2	0.9959	0.9911	0.9952	0.9996	--	--

Figure S5. Initial rate *KIE* for hydroalkoxylation.

4.6. Deuterium-labeling study

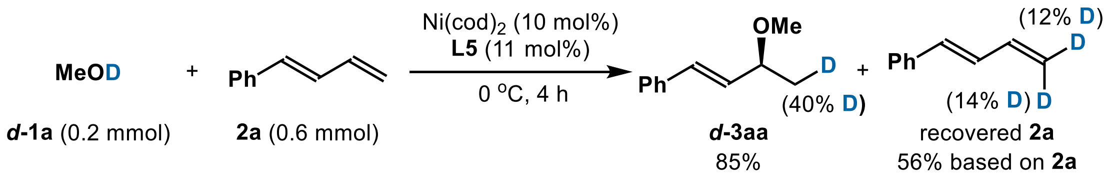

In a N_2-filled glovebox, (*R*, *R*)-Me-DuPhos (6.7 mg, 0.022 mmol) and 1,3-diene **2a** (78.1 mg, 0.6 mmol) were added to a 1-dram vial containing Ni(cod)$_2$ (5.6 mg, 0.02 mmol). The resulting mixture was stirred for 10 min and then ***d*-1a** (6.6 mg, 0.2 mmol) was added. The mixture was removed from the glovebox and was held at 0 °C for 4 h. The regioselectivity was determined by ^{1}H NMR analysis of the unpurified reaction mixture. The product ***d*-3aa** was purified by preparative thin-layer chromatography: 85%

yield, colorless oil, >20:1 *rr*. **^{1}H NMR** for ***d*-3aa** (400 MHz, $CDCl_3$) δ 7.42 – 7.36 (m, 2H), 7.32 (t, *J* = 7.5 Hz, 2H), 7.27 – 7.21 (m, 1H), 6.54 (d, *J* = 16.0 Hz, 1H), 6.10 (dd, *J* = 16.0, 7.6 Hz, 1H), 3.94 – 3.85 (m, 1H), 3.32 (s, 3H), 1.36 – 1.30 (m, 2.6H). **^{1}H NMR** for recovered diene (400 MHz, $CDCl_3$) δ 7.47 – 7.39 (m, 2H), 7.33 (t, *J* = 7.5 Hz, 2H), 7.28 – 7.19 (m, 1H), 6.81 (dd, *J* = 15.6, 10.5 Hz, 1H), 6.63 – 6.47 (m, 2H), 5.40 – 5.29 (m, 0.88H), 5.23 – 5.14 (m, 0.86H).

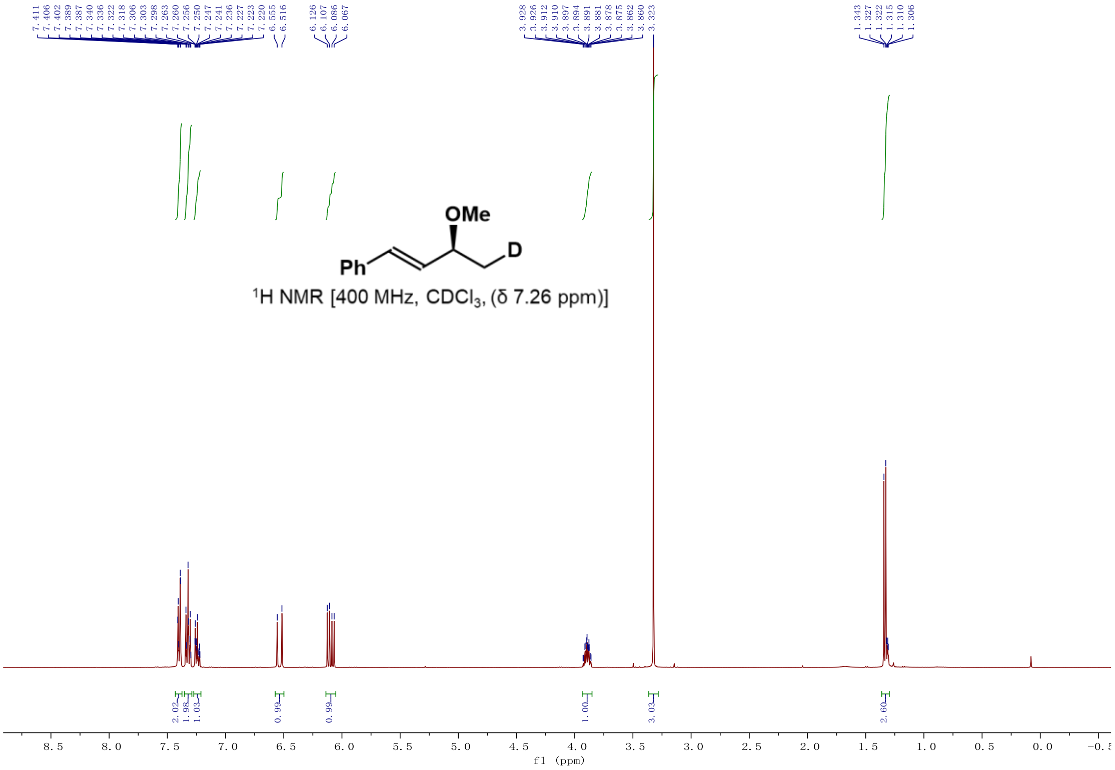

Figure S6. ^{1}H NMR [400 MHz, $CDCl_3$ (δ 7.26 ppm)] for ***d*-3aa**.

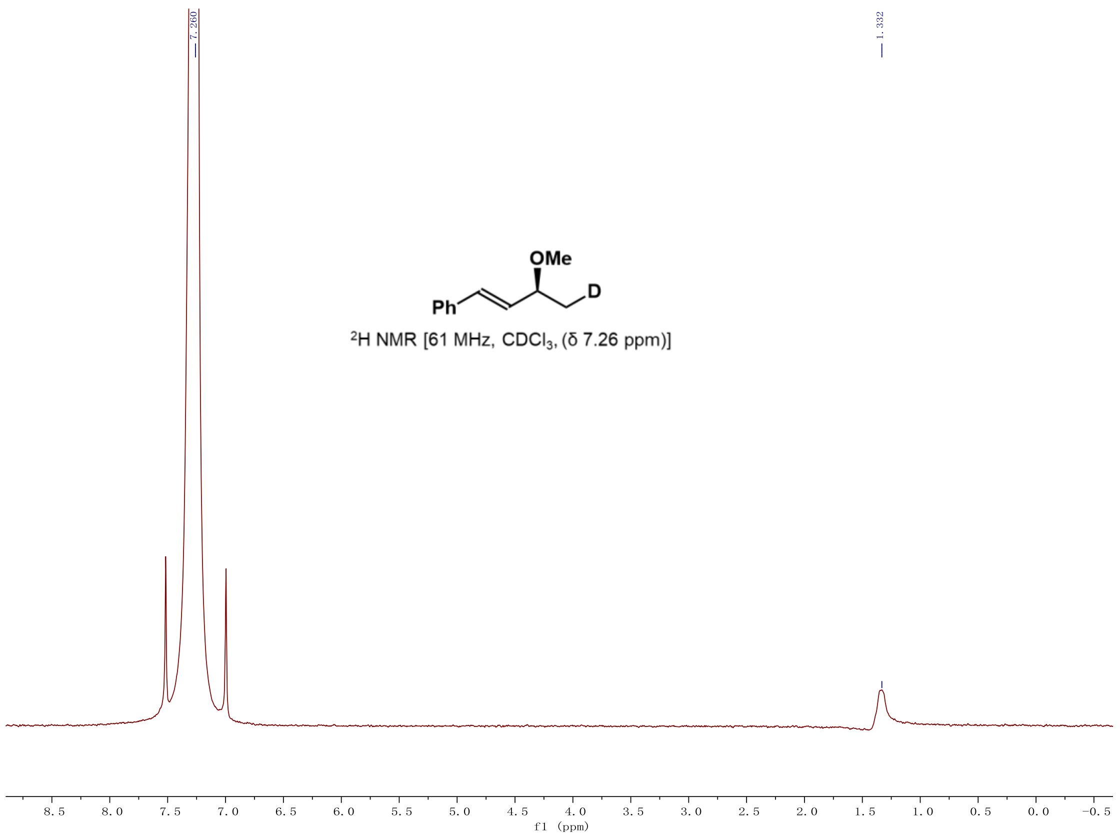

Figure S7. ^{2}H NMR [61 MHz, CDCl$_3$ (δ 7.26 ppm)] for ***d*-3aa**.

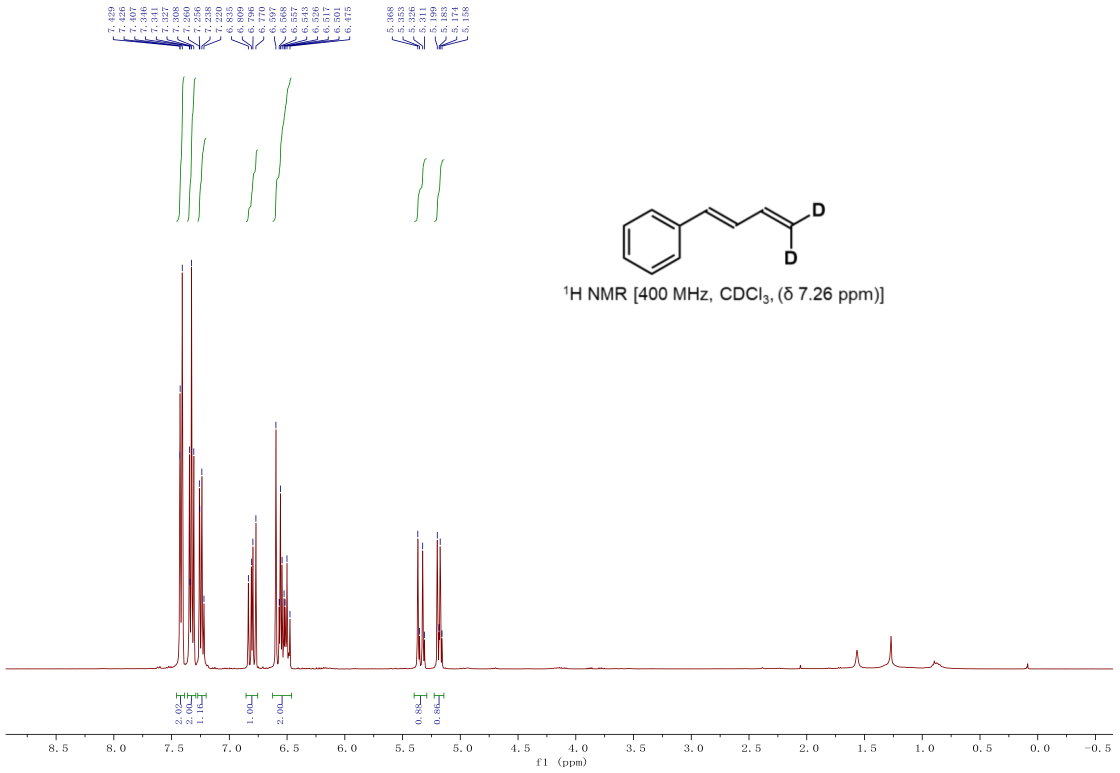

Figure S8. ^{1}H NMR [400 MHz, CDCl$_3$ (δ 7.26 ppm)] for recovered diene.

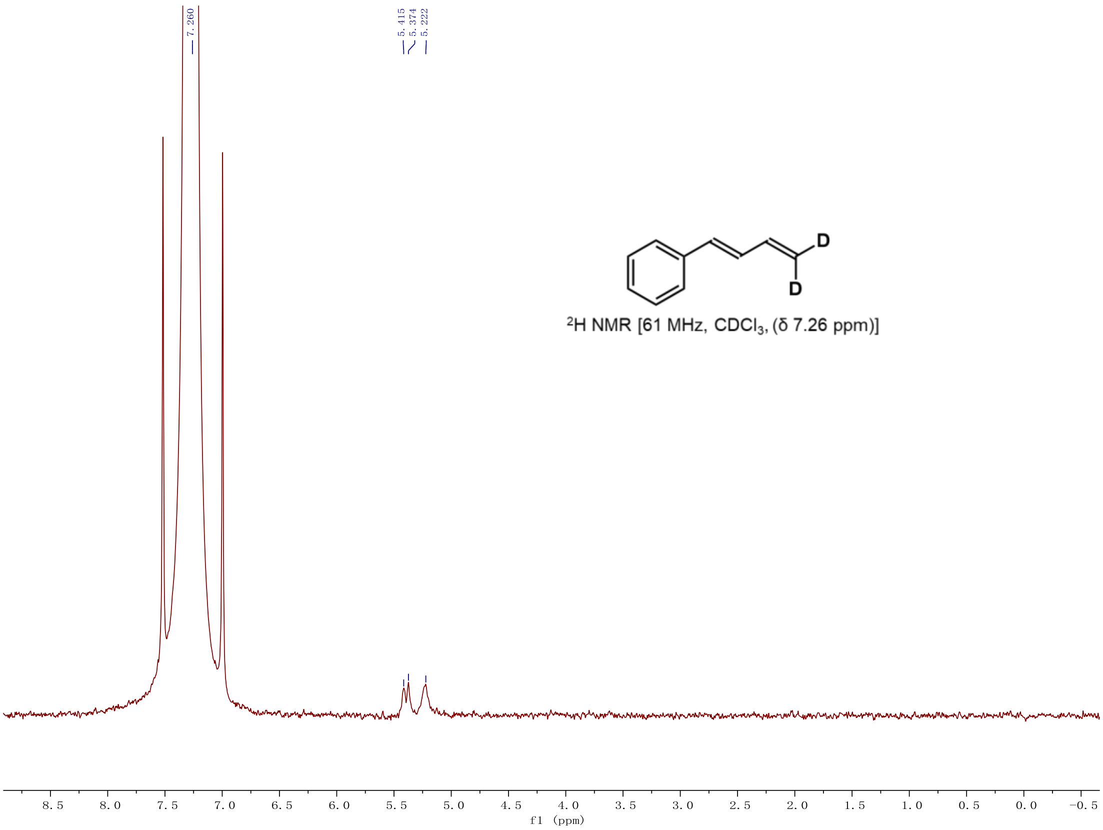

Figure S9. ^{2}H NMR [61 MHz, $CDCl_3$ (δ 7.26 ppm)] for recovered diene.

4.7. Diene geometry study

Synthesis of the mixture of (*Z*) and (*E*)-2a (3:1)

A mixture of (*Z*) and (*E*)-**2a** (3:1) was synthesized according to the literature.[13] **^{1}H NMR** for (*Z*) and (*E*)-**2a** (3:1) (400 MHz, $CDCl_3$) δ 7.35 – 7.21 (m, 4H), 7.19 – 7.12 (m, 1H), 6.90 – 6.67 (m, 1H), 6.53 – 6.13 (m, 2H), 5.34 – 5.22 (m, 1H), 5.17 – 5.07 (m, 1H).

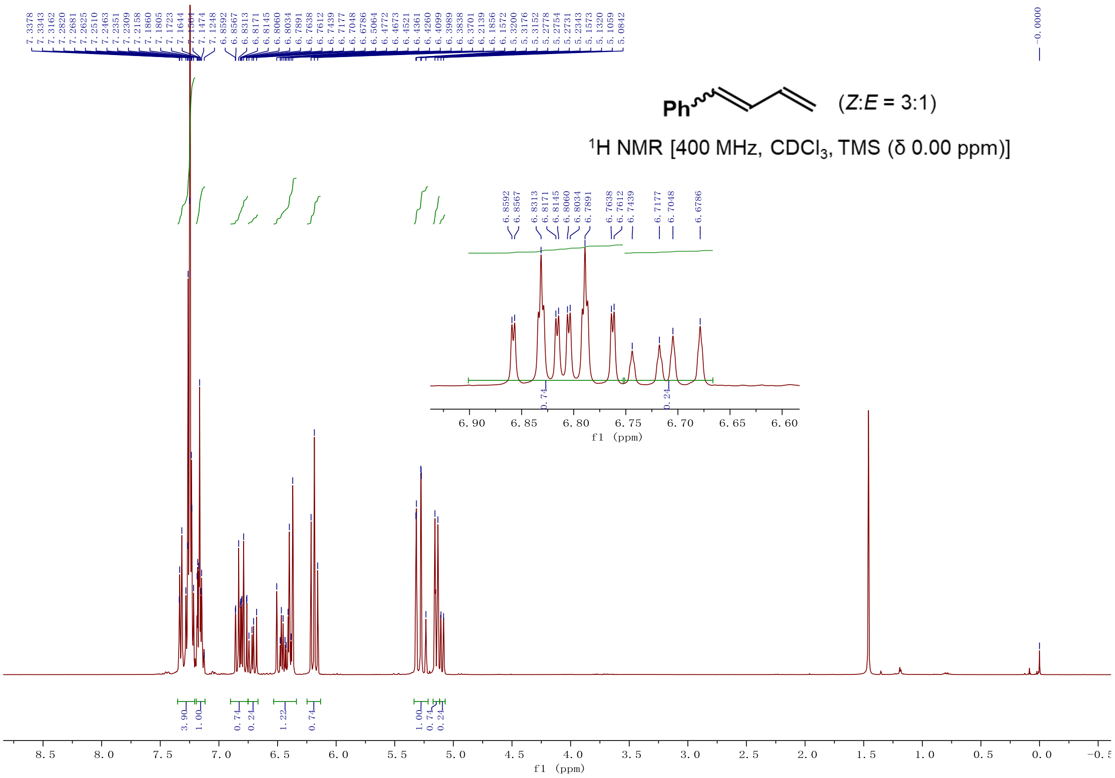

Figure S10. ^{1}H NMR [400 MHz, $CDCl_3$, TMS (δ 0.00 ppm)] for (*Z*) and (*E*)-**2a** (3:1).

Hydroalkoxylation of the mixture of (*Z*) and (*E*)-2a (3:1)

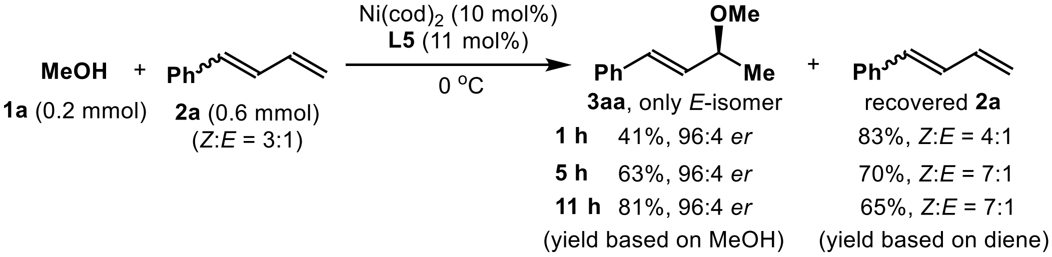

Following the "Method A" in "General procedure for hydroalkoxylation of 1,3-dienes", **2a** (*Z*:*E* = 3:1) was used to react with **1a**. Only the (*E*)**-3aa** was obtained: Colorless oil, 41% (1 h) – 81% (11 h) yield, >20:1 *rr*, 96:4 *er*. And a mixture of recovered (*Z*) and (*E*)-**2a** (4:1 (1 h) – 7:1 (11 h)) was recovered.

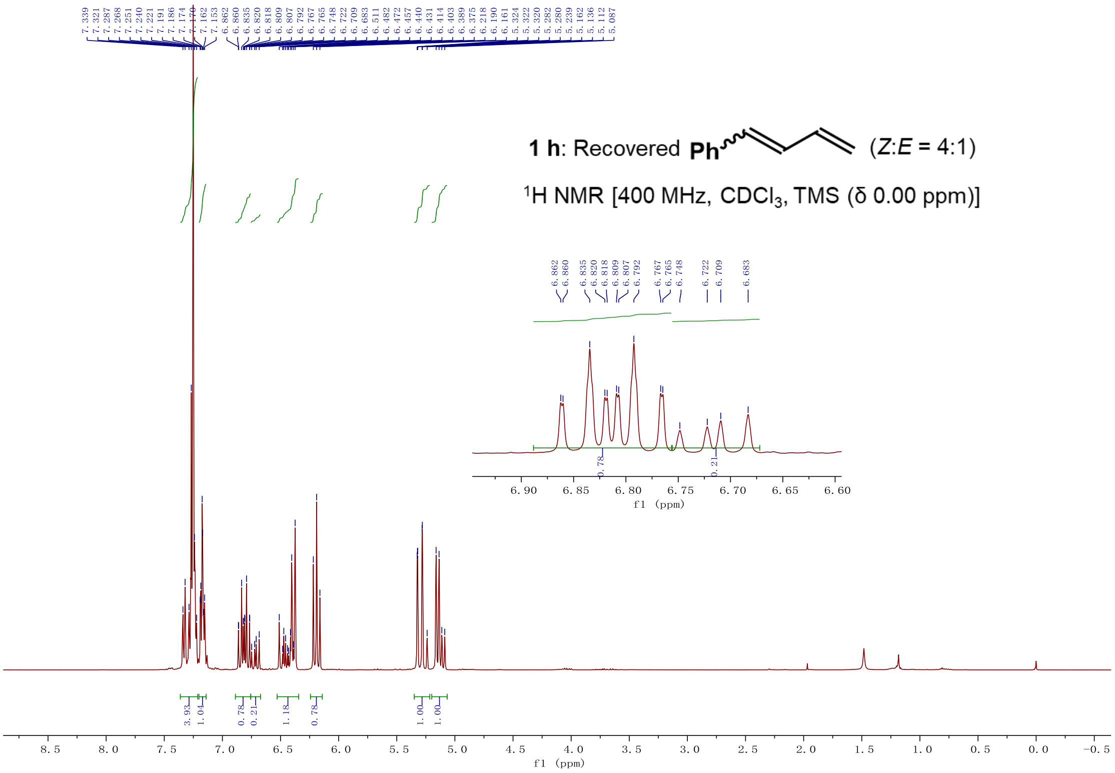

Figure S11. ^{1}H NMR [400 MHz, $CDCl_3$, TMS (δ 0.00 ppm)] for recovered (*Z*) and (*E*)-**2a** (4:1).

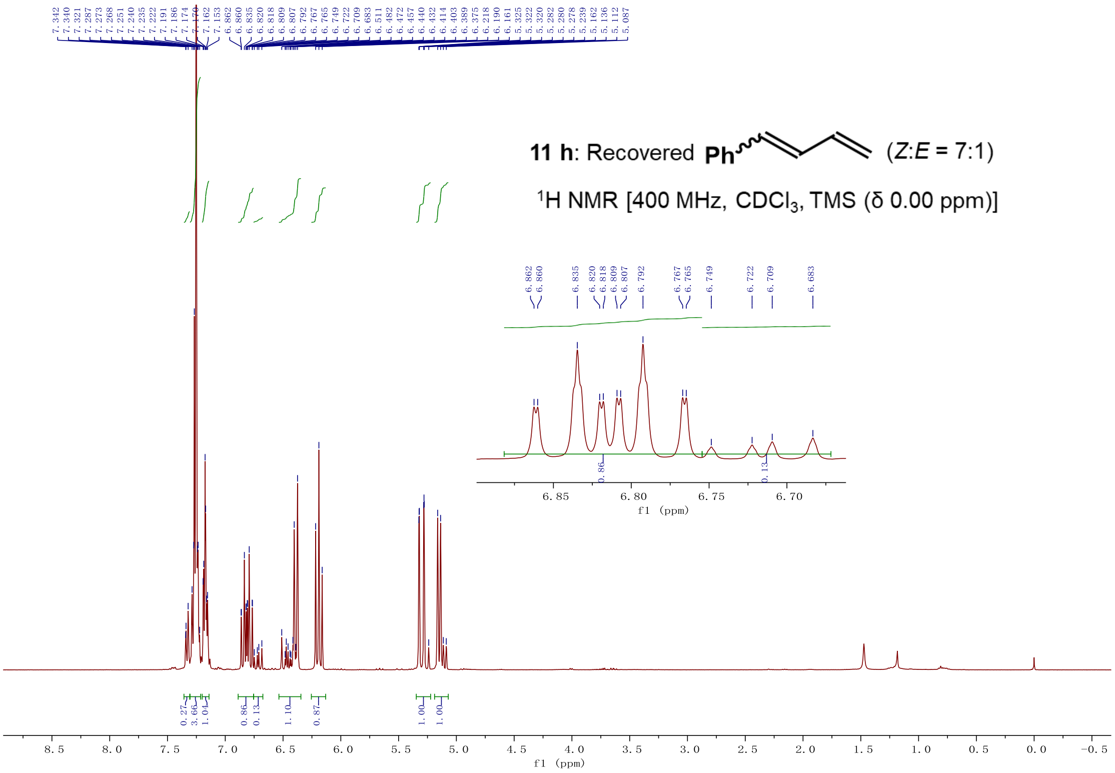

Figure S12. ^{1}H NMR [400 MHz, $CDCl_3$, TMS (δ 0.00 ppm)] for recovered (*Z*) and (*E*)-**2a** (7:1).

We propose isomerization occurs by protonation (via LLHT) of (*Z*)-**2a** to generate a (*Z*)-allylic nickel intermediate **A**. A familiar σ-π-σ process occurs to afford intermediate **B**, which converts to conformation **B'** via bond rotation. **B'** also undergoes σ-π-σ isomerization followed by β-H elimination to give (*E*)-**2a** (Figure 4).[13] Alternatively, intermediate **C** may form the metal-π-allyl intermediate and then the product directly.

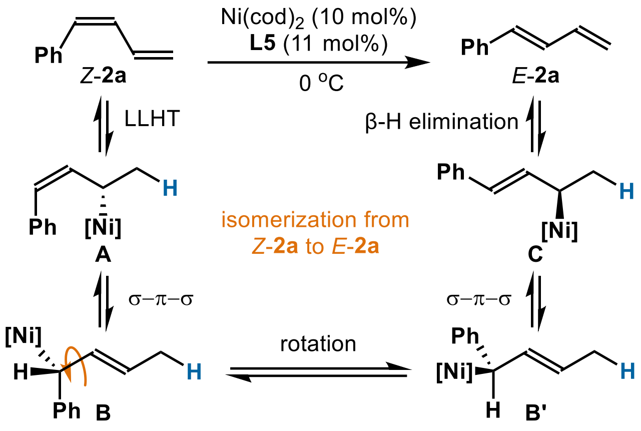

4.8. Cross-over Studies

Without solvent

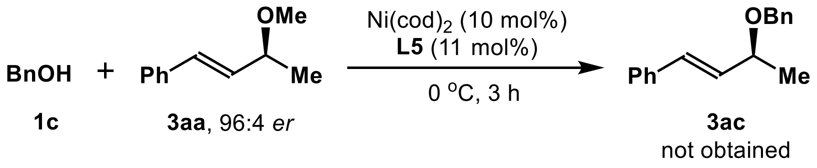

In a N_2-filled glovebox, (*R*, *R*)-Me-DuPhos (2.6 mg, 0.0084 mmol), **3aa** (37 mg, 0.228 mmol, 96:4 *er*), and **1c** (8.2 mg, 0.076 mmol) were added to a 1-dram vial containing $Ni(cod)_2$ (2.1 mg, 0.0076 mmol). The resulting mixture was removed from the glovebox and stirred at 0 °C for 3 h. No trace of **3ac** was obtained according to the ^1H NMR analysis of the unpurified reaction mixture. Enantioselectivity were determined by HPLC. **3aa** and recovered **3aa**: 96:4 *er*, chiral stationary column OJ-H, mobile phase hexane/^{i}PrOH = 99/1, 1.0 mL/min, 254 nm, 30 °C, t_R (minor) = 6.9 min, t_R (major) = 7.4 min.

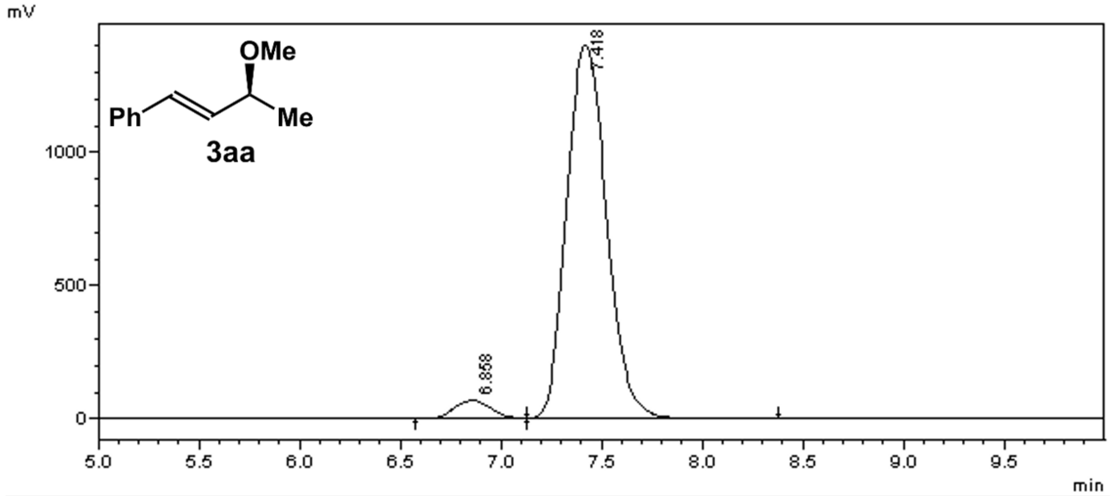

峰表

检测器 A Ch1 254nm

峰#	保留时间	面积	高度	面积 %
1	6.858	864070	67818	4.265
2	7.418	19394157	1399002	95.735

Translation of Chinese characters to English:

Peak	Retention time	Area	Height	Area %
1	6.858	864070	67818	4.265
2	7.418	19394157	1399002	95.735

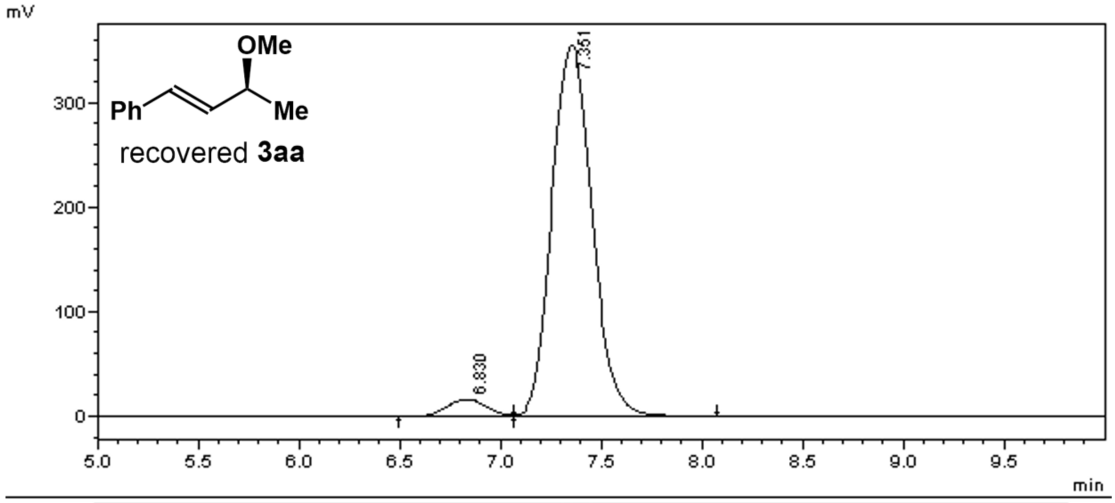

峰表

检测器 A Ch1 254nm

峰#	保留时间	面积	高度	面积 %
1	6.830	219608	16201	4.269
2	7.351	4924816	354213	95.731

Translation of Chinese characters to English:

Peak	Retention time	Area	Height	Area %
1	6.830	219608	16201	4.269
2	7.351	4924816	354213	95.731

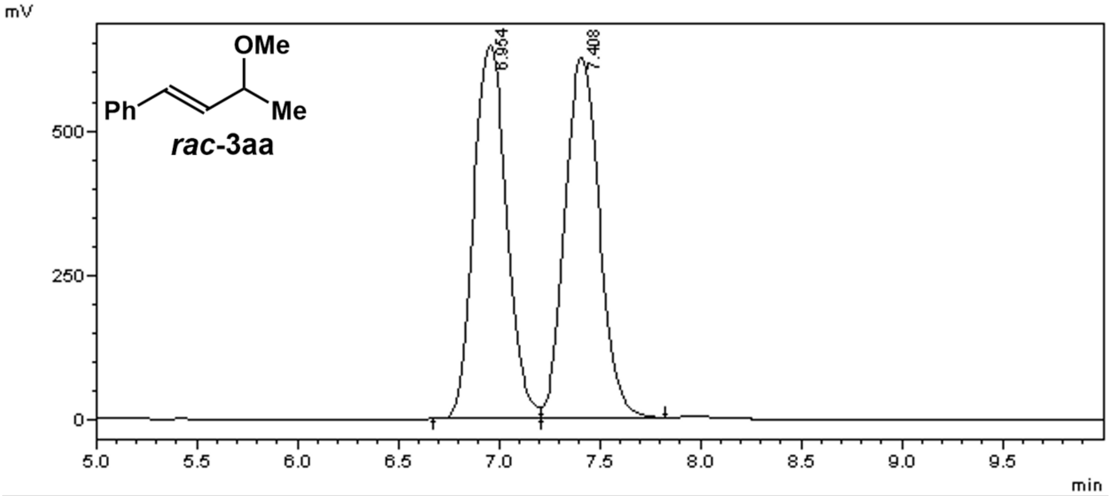

峰表

检测器 A Ch1 254nm

峰#	保留时间	面积	高度	面积 %
1	6.954	7361837	646214	49.697
2	7.408	7451494	624049	50.303

Translation of Chinese characters to English:

Peak	Retention time	Area	Height	Area %
1	6.954	7361837	646214	49.697
2	7.408	7451494	624049	50.303

With solvent

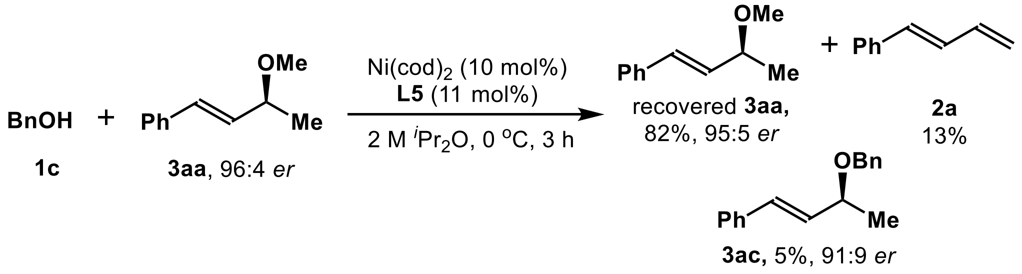

In a N_2-filled glovebox, (*R*, *R*)-Me-DuPhos (1.7 mg, 0.0055 mmol) and $^{i}Pr_2O$ (25 μL) were added to a 1-dram vial containing Ni(cod)$_2$ (1.4 mg, 0.005 mmol). The resulting mixture was stirred for 10 min, and then **3aa** (24.3 mg, 0.15 mmol, 96:4 *er*) and **1c** (5.4 mg, 0.05 mmol) were added. The mixture was stirred at 0 °C for 3 h. The amount of **3ac**, **2a**, and **3aa** (5%, 13%, and 82%) was determined by ^{1}H NMR analysis of the unpurified reaction mixture. Enantioselectivity were determined by HPLC. Recovered

3aa: 95:5 *er*, chiral stationary column OJ-H, mobile phase hexane/^{i}PrOH = 99/1, 1.0 mL/min, 254 nm, 30 °C, t_R (minor) = 7.0 min, t_R (major) = 7.6 min; **3ac**: 91:9 *er*, chiral stationary column OJ-H, mobile phase hexane/^{i}PrOH = 90/10, 1.0 mL/min, 254 nm, 30 °C, t_R (minor) = 7.3 min, t_R (major) = 8.7 min.

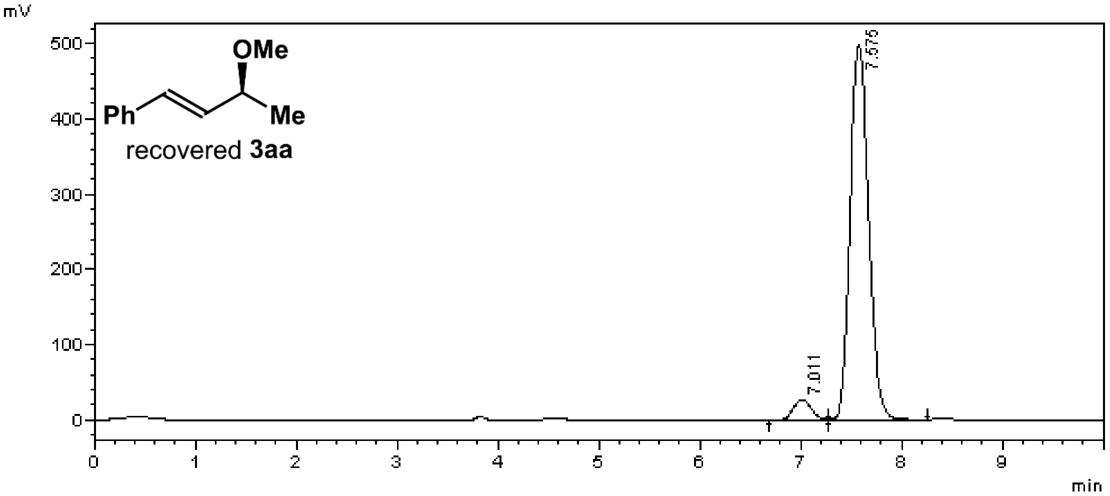

峰表

检测器 A Ch1 254nm

峰#	保留时间	面积	高度	面积 %
1	7.011	332321	26915	4.954
2	7.575	6375935	497740	95.046

Translation of Chinese characters to English:

Peak	Retention time	Area	Height	Area %
1	7.011	332321	26915	4.954
2	7.575	6375935	497740	95.046

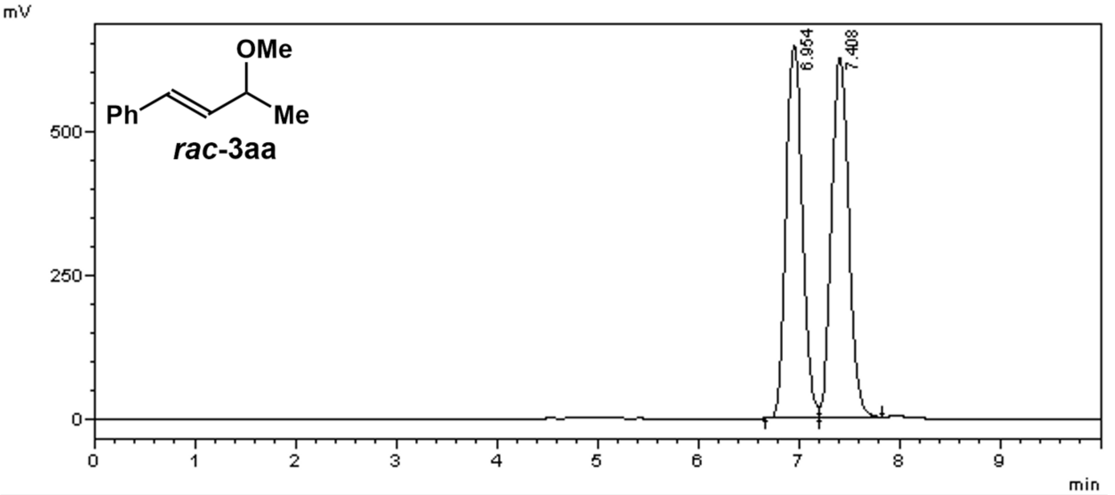

峰表

检测器 A Ch1 254nm

峰#	保留时间	面积	高度	面积 %
1	6.954	7361837	646214	49.697
2	7.408	7451494	624049	50.303

Translation of Chinese characters to English:

Peak	Retention time	Area	Height	Area %
1	6.954	7361837	646214	49.697
2	7.408	7451494	624049	50.303

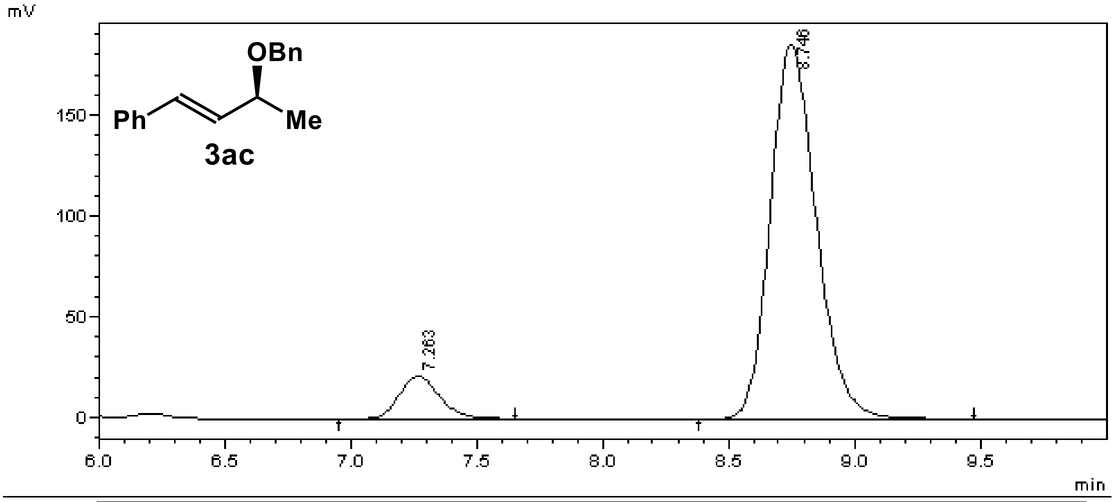

峰表

检测器 A Ch1 254nm

峰#	保留时间	面积	高度	面积 %
1	7.263	233519	21198	9.007
2	8.746	2359049	185349	90.993

Translation of Chinese characters to English:

Peak	Retention time	Area	Height	Area %
1	7.263	233519	21198	9.007
2	8.746	2359049	185349	90.993

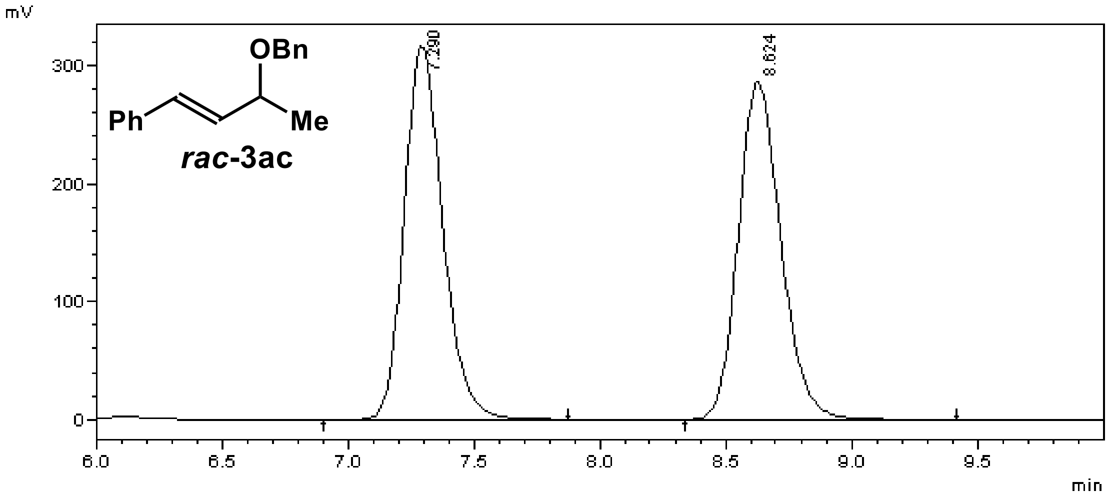

峰表

检测器 A Ch1 254nm

峰#	保留时间	面积	高度	面积 %
1	7.290	3369964	316453	50.005
2	8.624	3369253	285818	49.995

Translation of Chinese characters to English:

Peak	Retention time	Area	Height	Area %
1	7.290	3369964	316453	50.005
2	8.624	3369253	255818	49.995

4.9. Reversibility study for branched 1,3-diene

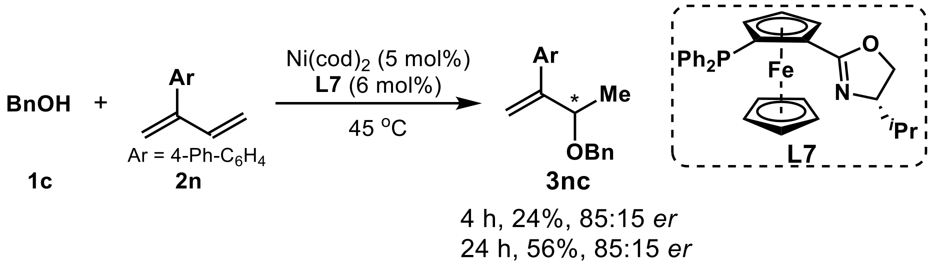

In a N_2-filled glovebox, **L7** (5.7 mg, 0.012 mmol), 1,3-diene **2n** (41.3 mg, 0.2 mmol), and alcohol **1c** (86.5 mg, 0.8 mmol) were added to a 1-dram vial containing Ni(cod)$_2$ (2.8 mg, 0.01 mmol). The resulting mixture was stirred at 45 °C. Yields were obtained by ^{1}H NMR analysis of the unpurified reaction mixture. The product was purified by preparative thin-layer chromatography. The ^{1}H NMR spectral data matches the literature.[8] Enantioselectivity were determined by HPLC. **3nc** (4 h and 24 h): 85:15 *er*,

chiral stationary column OD-H, mobile phase hexane/^{i}PrOH = 99/1, 1.0 mL/min, 254 nm, 30 °C, t_R (major) = 6.2 min, t_R (minor) = 6.9 min.

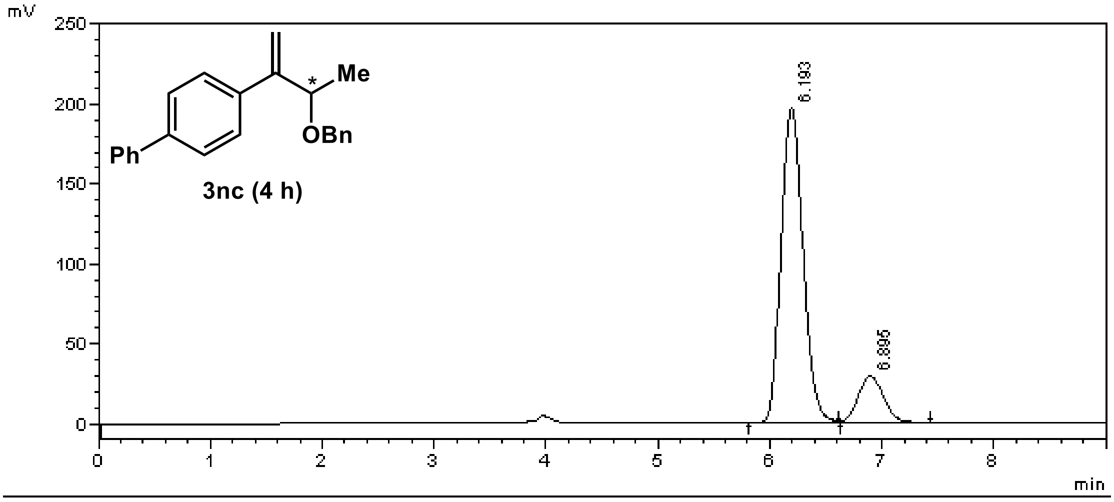

峰表

检测器 A Ch1 254nm

峰#	保留时间	面积	高度	面积 %
1	6.193	2715739	197192	84.924
2	6.895	482118	29476	15.076

Translation of Chinese characters to English:

Peak	Retention time	Area	Height	Area %
1	6.193	1715739	197192	84.924
2	6.895	482118	29476	15.076

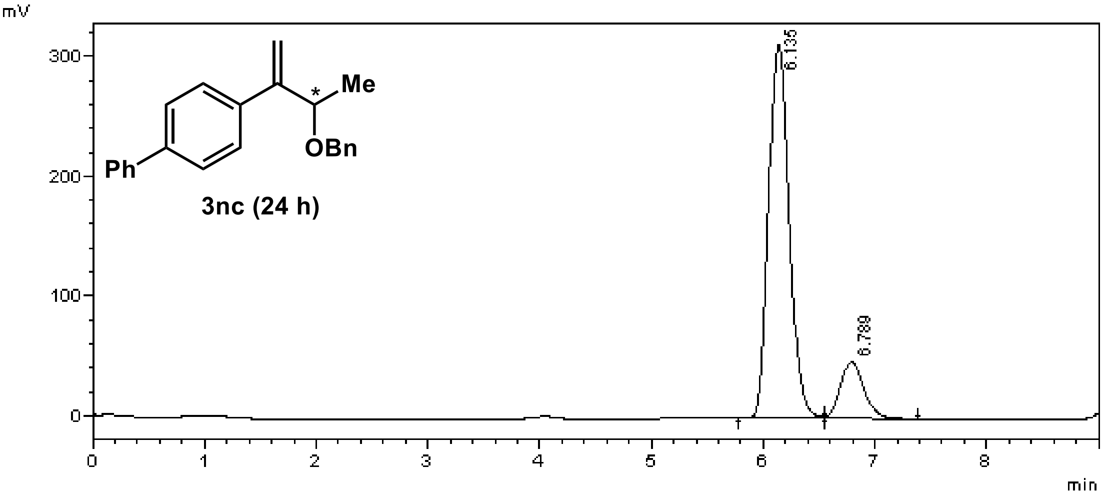

峰表

检测器 A Ch1 254nm

峰#	保留时间	面积	高度	面积 %
1	6.135	4009586	311455	85.318
2	6.789	689990	46830	14.682

Translation of Chinese characters to English:

Peak	Retention time	Area	Height	Area %
1	6.135	4009586	311455	85.318
2	6.789	689990	46830	14.682

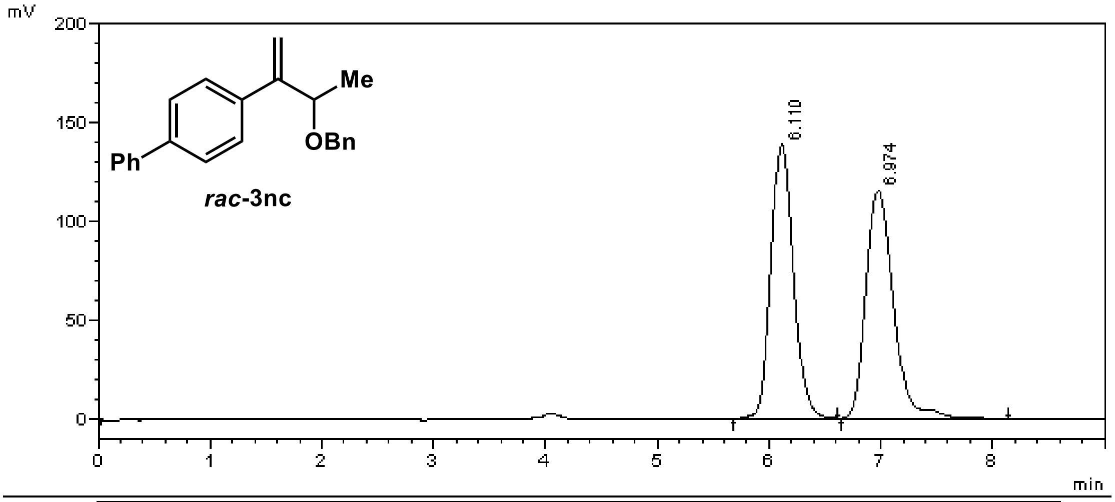

峰表

检测器 A Ch1 254nm

峰#	保留时间	面积	高度	面积 %
1	6.110	1954245	138467	49.619
2	6.974	1984256	114745	50.381

Translation of Chinese characters to English:

Peak	Retention time	Area	Height	Area %
1	6.110	1954245	138467	49.619
2	6.974	1984256	114745	50.381

5. References

1. Pünner, F.; Schmidt, A.; Hilt, G. Corrigendum: Up the Hill: Selective Double-Bond Isomerization of Terminal 1,3-Dienes towards *Z*-1,3-Dienes or 2*Z*,4*E*-Dienes. *Angew. Chem. Int. Ed.* **2019,** *58*, 17103.
2. Li, M.-M.; Cheng, L.; Xiao, L.-J.; Xie, J.-H.; Zhou, Q.-L. Palladium-Catalyzed Asymmetric Hydrosulfonylation of 1,3-Dienes with Sulfonyl Hydrazides. *Angew. Chem. Int. Ed.* **2021,** *60*, 2948.
3. Dongbang, S.; Ellman, J. A. Synthesis of Nitrile Bearing Acyclic Quaternary Centers through Co(III)-Catalyzed Sequential C−H Bond Addition to Dienes and *N*-Cyanosuccinimide. *Angew. Chem. Int. Ed.* **2021**, *60*, 2135.
4. Malkov, A. V.; Baxendale, I. R.; Dvořák, D.; Mansfield, D. J.; Kočovský, P. Molybdenum(II)- and Tungsten(II)-Catalyzed Allylic Substitution. *J. Org. Chem.* **1999**, *64*, 2737.
5. Tan, J.; Zhang, Z.; Wang, Z. A Novel Palladium-Catalyzed Hydroalkoxylation of Alkenes with A Migration of Double Bond. *Org. Biomol. Chem.* **2008**, *6*, 1344.
6. Ouyang, L.; Xia, Y.; Miao, R.; Liao, J.; Luo, R. Iridium-Catalyzed Reductive Etherification of α,β-Unsaturated Ketones and Aldehydes with Alcohols. *Org. Biomol. Chem.* **2022**, *20*, 2621.
7. Aggarwal, S. K.; Bradshaw, J. S.; Eguchi, M.; Parry, S.; Rossiter, B. E.; Markides, K. E.; Lee, M. L. New Chiral Polysiloxanes Prepared from Derivatives of (+)- or (-)-2-phenyl-3-butehoic Acid, (r)-l-hepten-3-ol and (r)-l-cyclohexyl-2-propen-1-ol. *Tetrahedron* **1987**, *43*, 451.
8. Tran, G.; Mazet, C. Ni-Catalyzed Regioselective Hydroalkoxylation of Branched 1,3-Dienes. *Org. Lett.* **2019**, *21*, 9124.
9. Feng, B.; Cheng, H.-G.; Chen, J.-R.; Deng, Q.-H.; Lu, L.-Q.; Xiao, W.-J. Palladium/Sulfoxide–Phosphine-Catalyzed Highly Enantioselective Allylic Etherification and Amination. *Chem. Commun.* **2014**, *50*, 9550.
10. Biswas, S.; Samec, J. S. M. The Efficiency of the Metal Catalysts in the Nucleophilic Substitution of Alcohols is Dependent on the Nucleophile and Not on the Electrophile. *Chem. Asian J.* **2013**, *8*, 974.

11. a) Nielsen, C. D.-T.; Burés, J. Visual Kinetic Analysis. *Chem. Sci.* **2019**, *10*, 348; b) Burés, J. Variable Time Normalization Analysis: General Graphical Elucidation of Reaction Orders from Concentration Profiles. *Angew. Chem. Int. Ed.* **2016**, *55*, 16084.
12. Brunker, T. J.; Blank, N. F.; Moncarz, J. R.; Scriban, C.; Anderson, B. J.; Glueck, D. S.; Zakharov, L. N.; Golen, J. A.; Sommer, R. D.; Incarvito, C. D.; Rheingold, A. L. Chiral Palladium(0) *trans*-Stilbene Complexes: Synthesis, Structure, and Oxidative Addition of Phenyl Iodide. *Organometallics* **2005**, *24*, 2731.
13. Wang, W.; He, S.; Zhong, Y.; Chen, J.; Cai, C.; Luo, Y.; Xia, Y. Cobalt-Catalyzed *Z* to *E* Geometrical Isomerization of 1,3-Dienes. *J. Org. Chem.* **2022**, *87*, 4712.

6. NMR spectra

(*S*,*E*)-(3-methoxybut-1-en-1-yl)benzene (3aa)

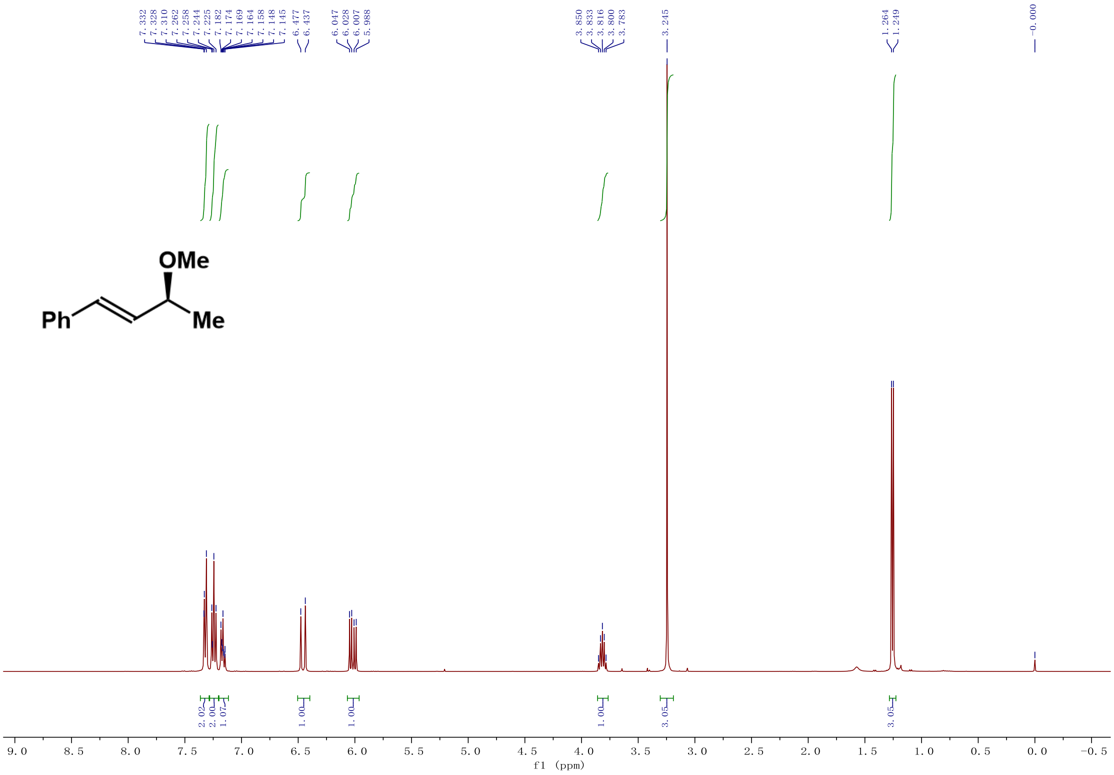

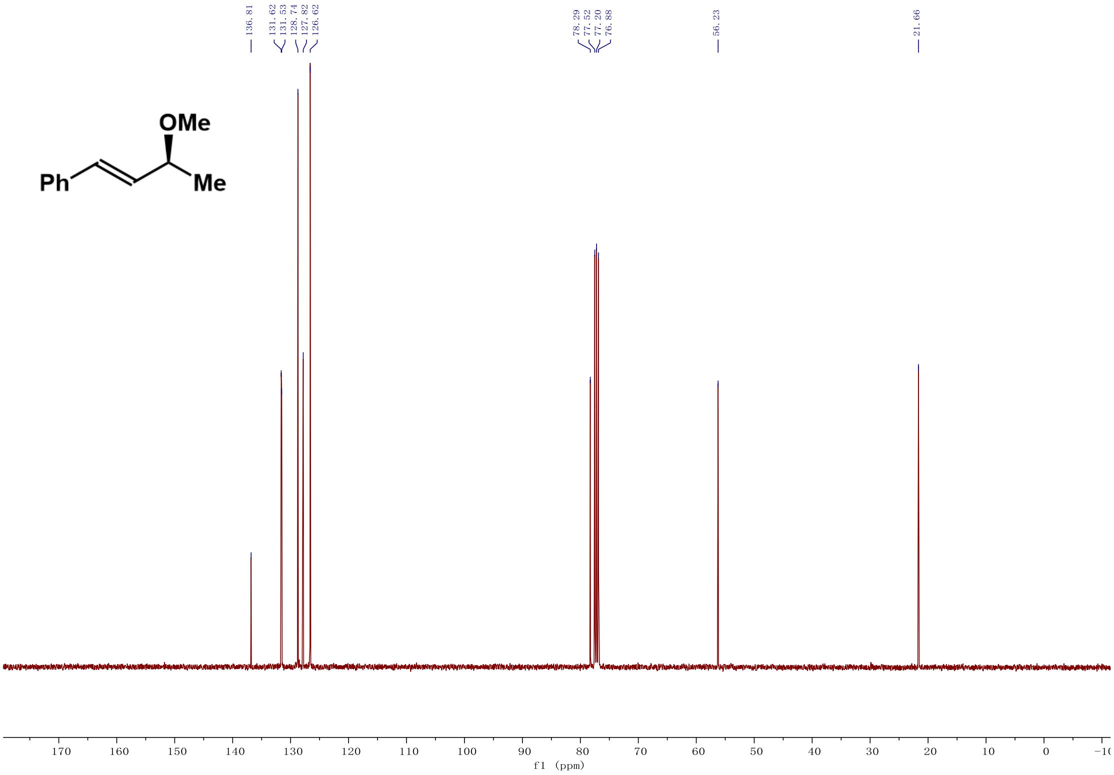

(*S*,*E*)-1-methoxy-4-(3-methoxybut-1-en-1-yl)benzene (3ba)

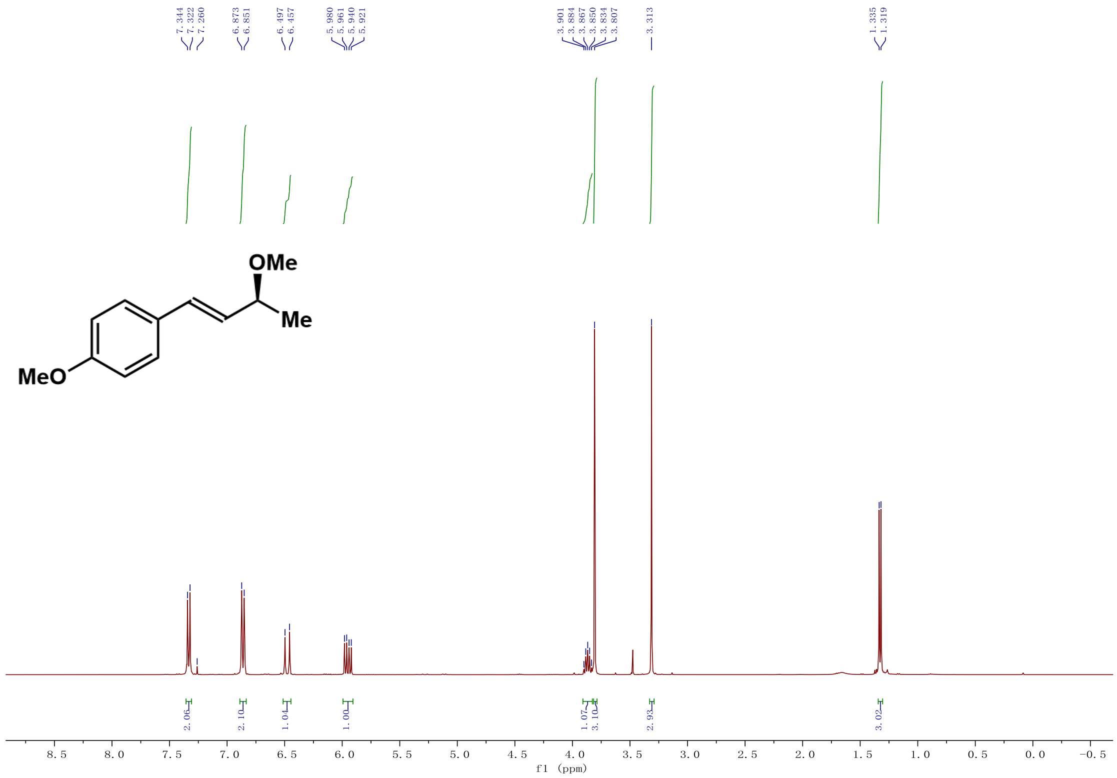

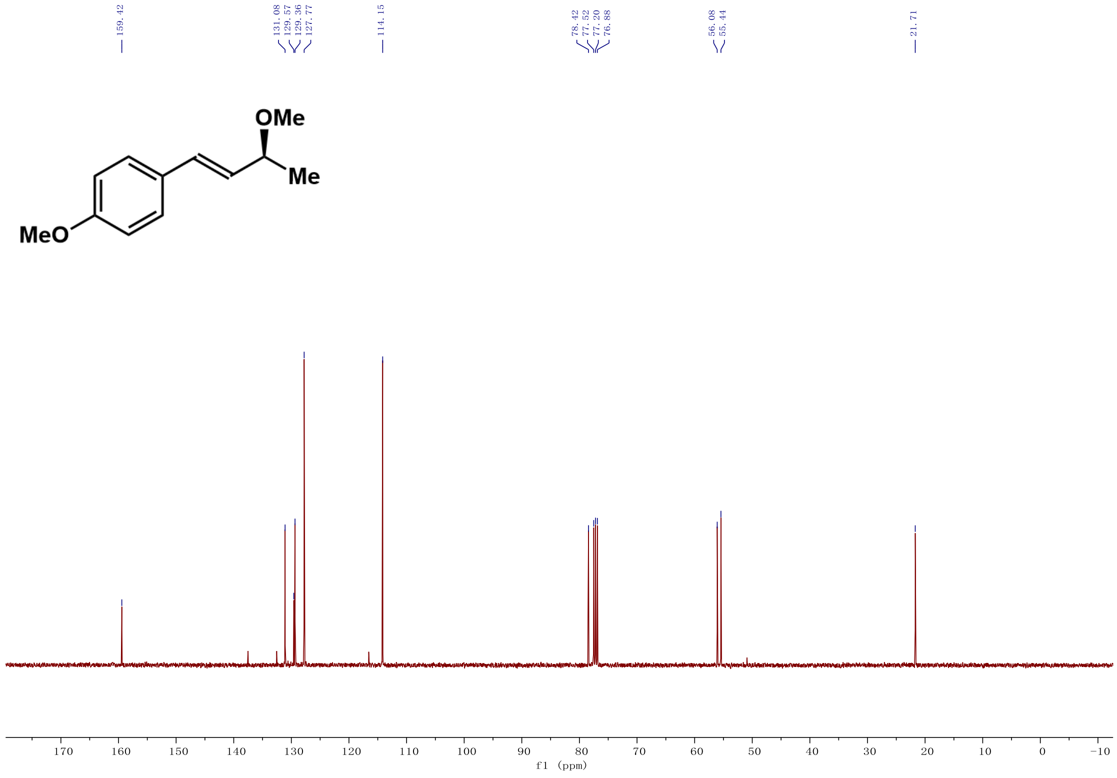

(*S,E*)-1-(3-methoxybut-1-en-1-yl)-4-methylbenzene (3ca)

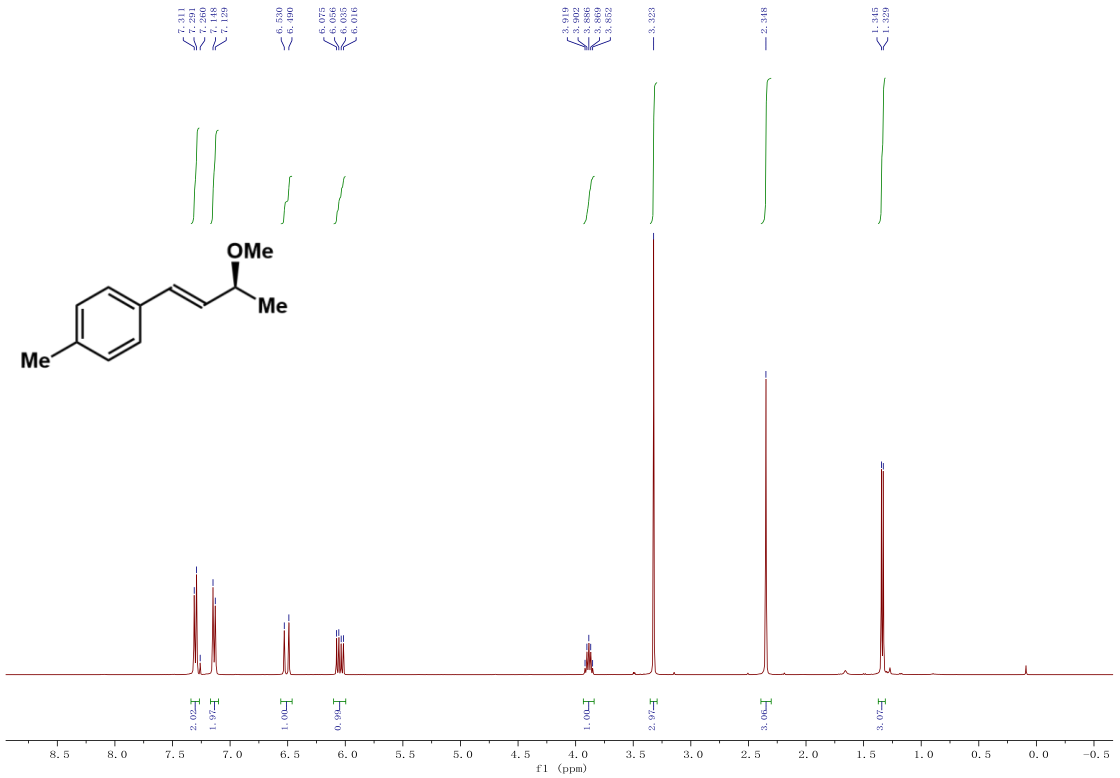

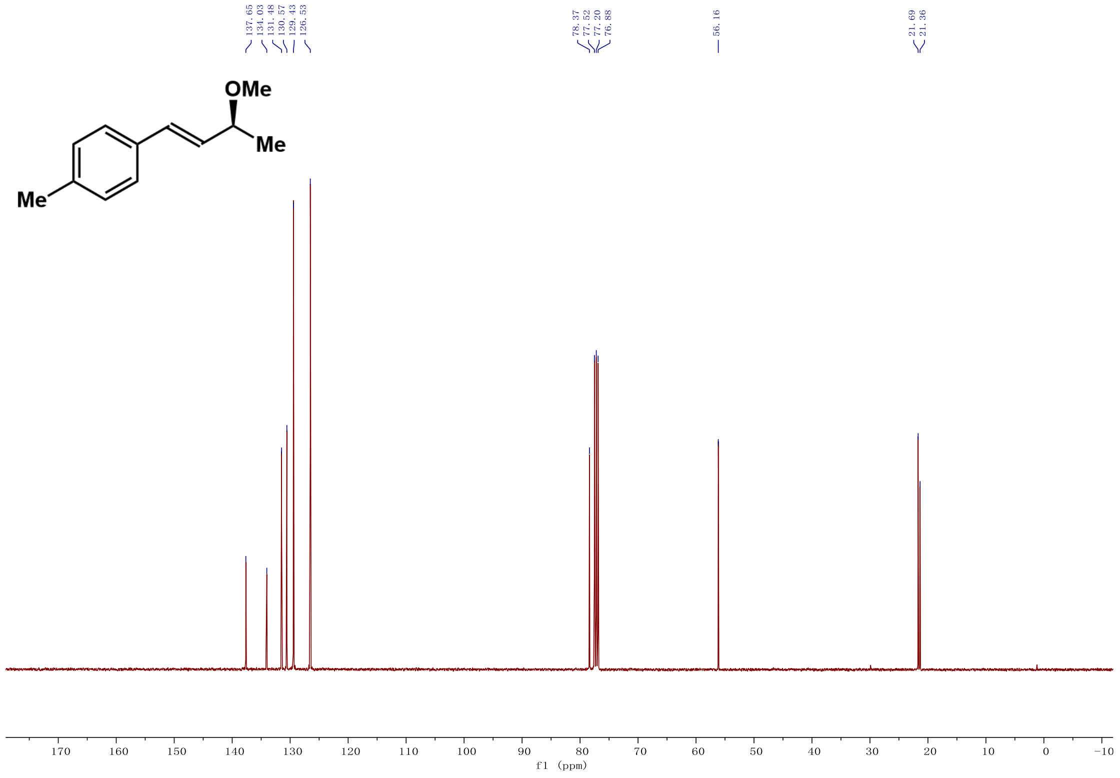

(*S,E*)-1-fluoro-4-(3-methoxybut-1-en-1-yl)benzene (3da)

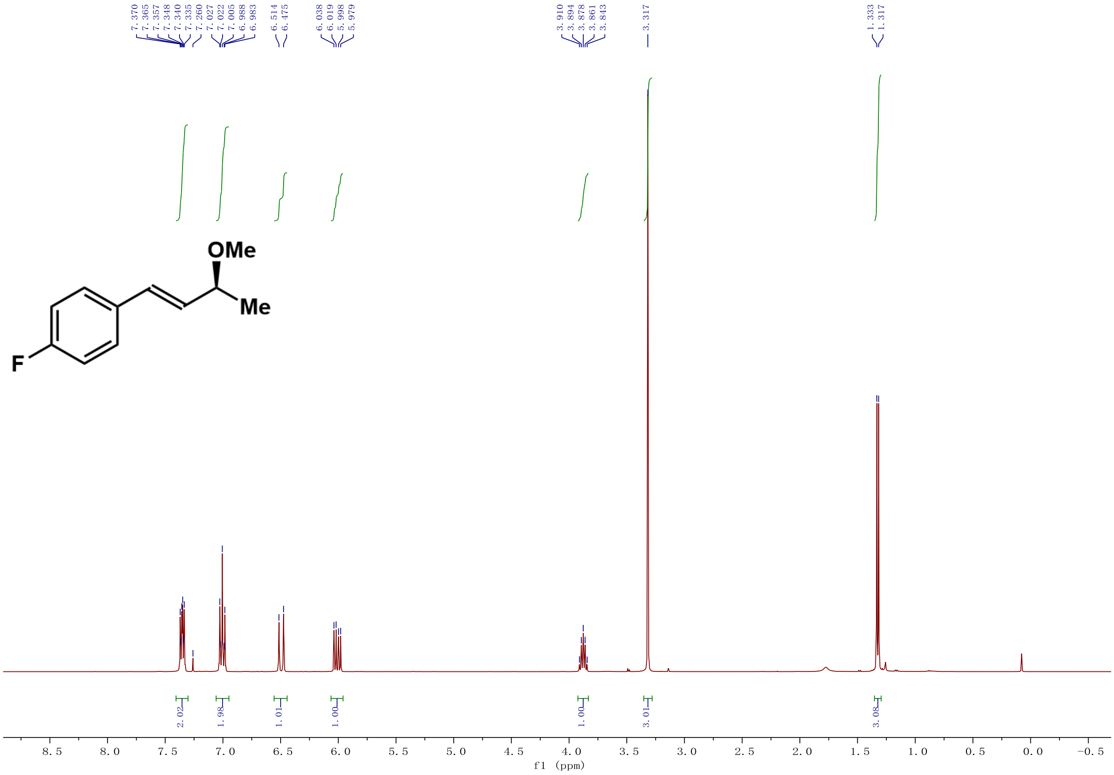

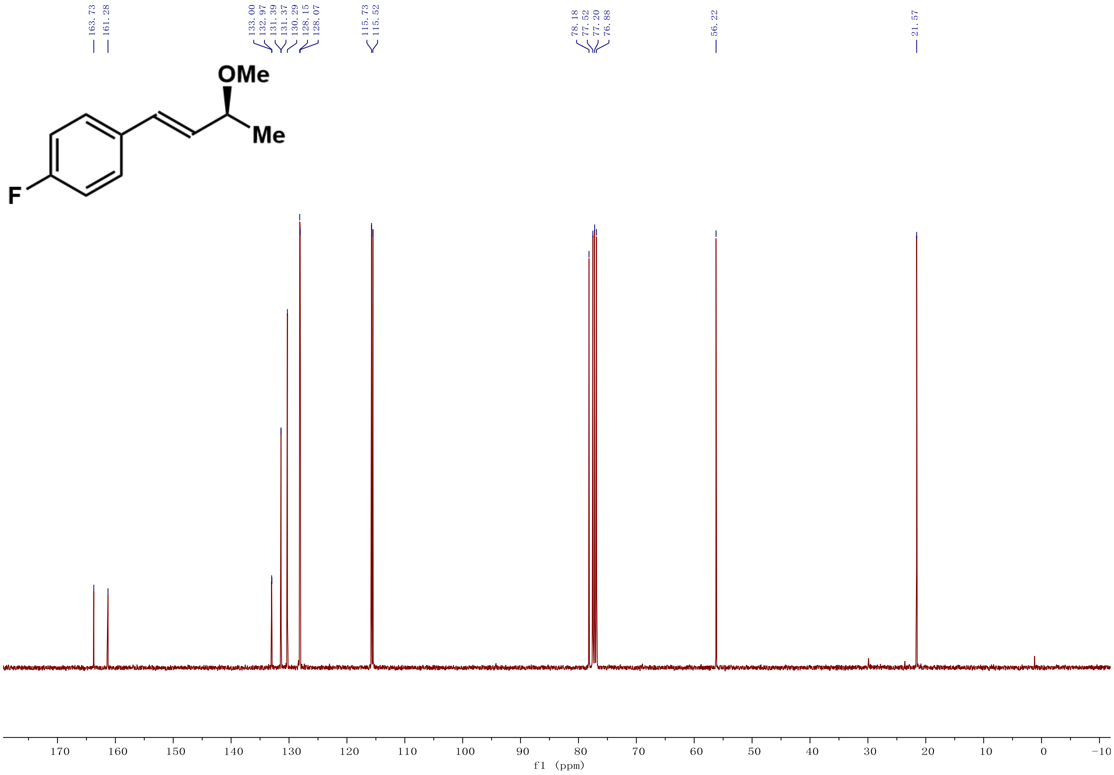

(*S*,*E*)-1-chloro-4-(3-methoxybut-1-en-1-yl)benzene (3ea)

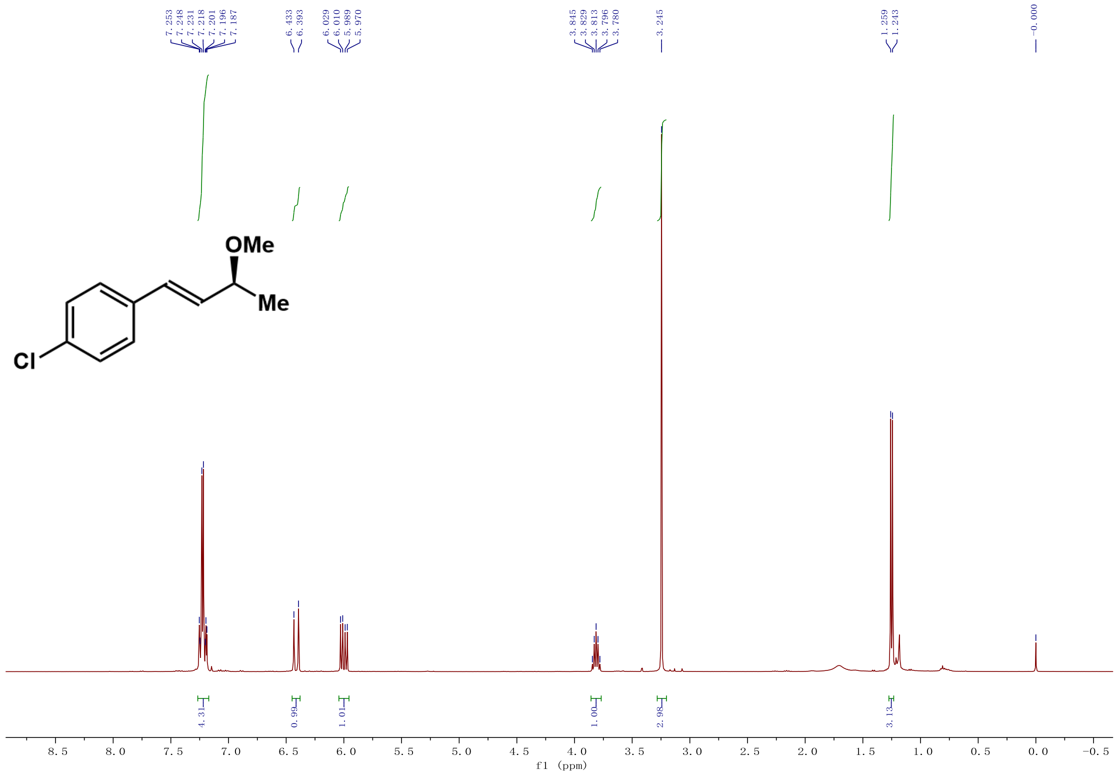

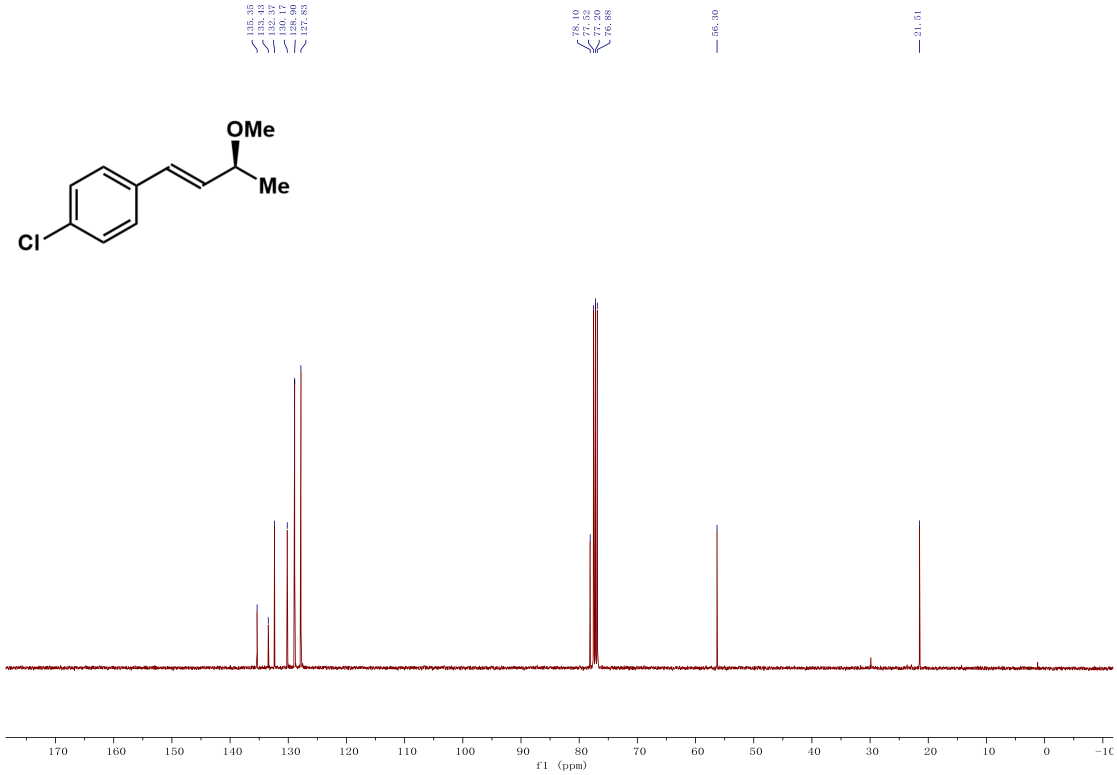

(*S,E*)-4-(3-methoxybut-1-en-1-yl)-*N,N*-dimethylaniline (3fa)

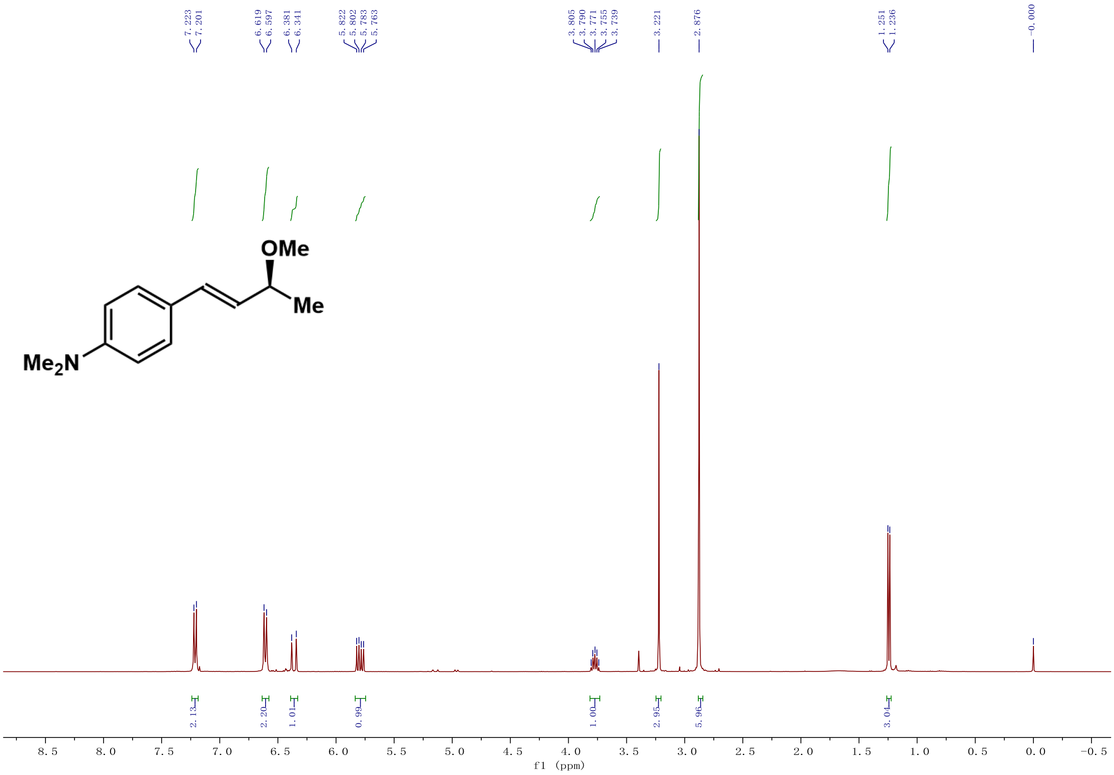

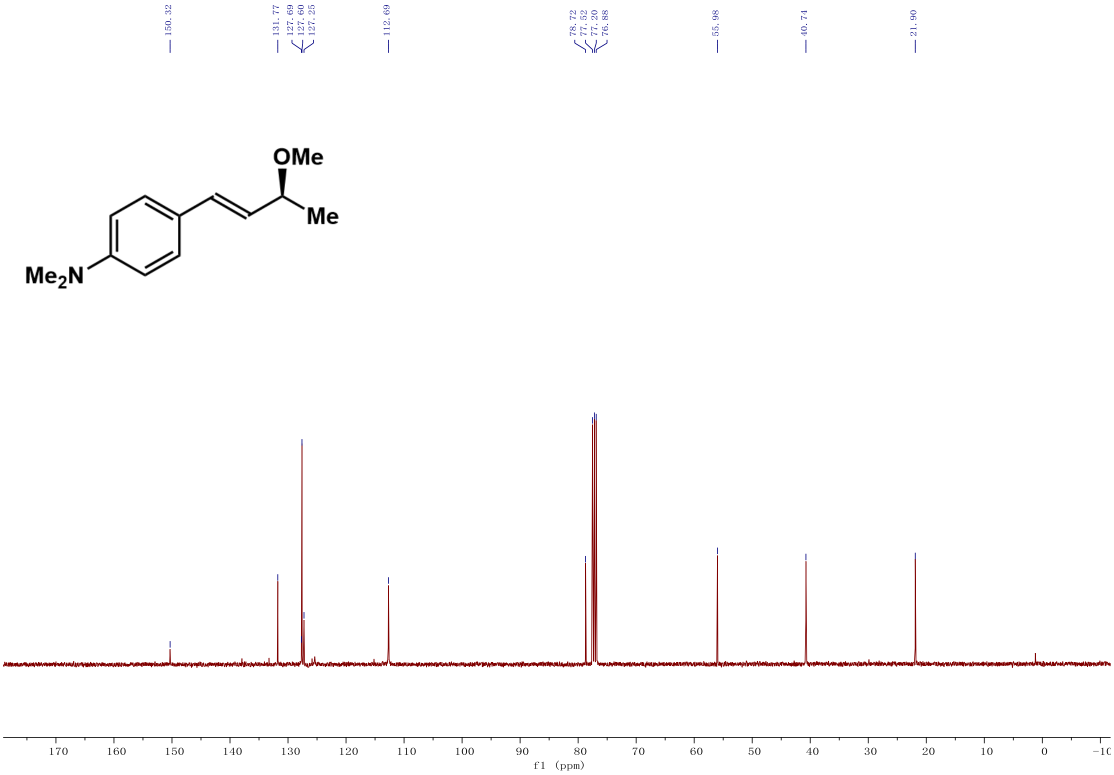

(*S*,*E*)-1-(3-methoxybut-1-en-1-yl)-3-methylbenzene (3ga)

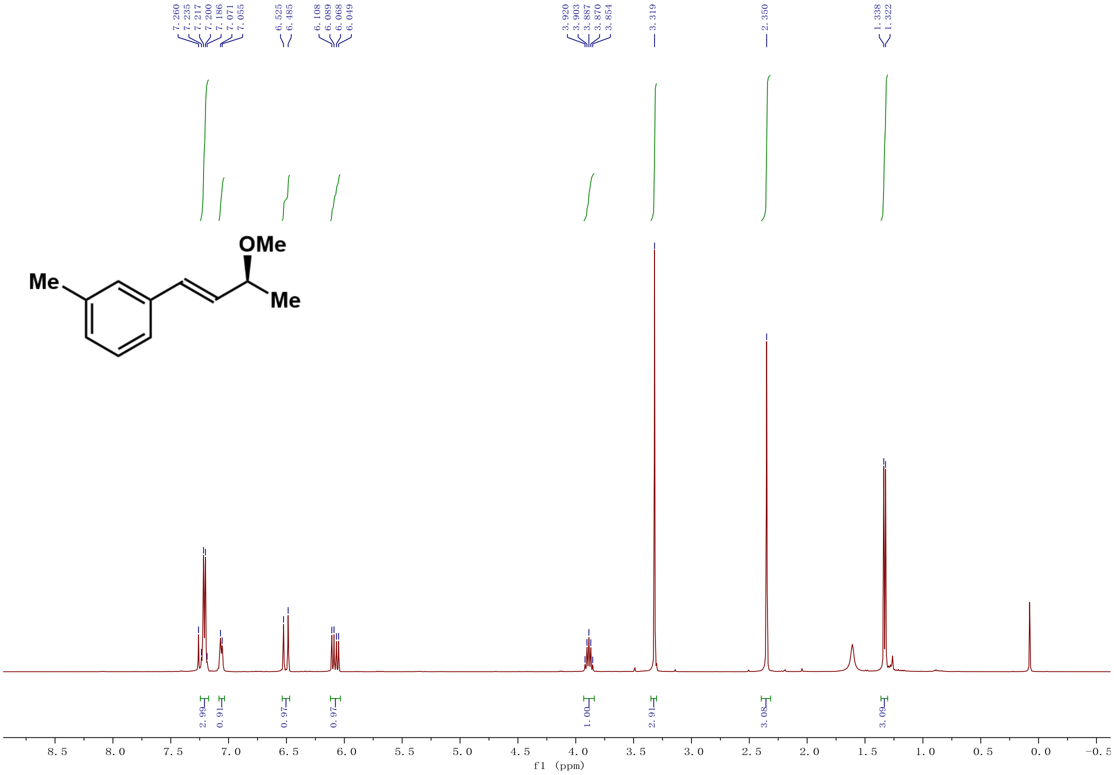

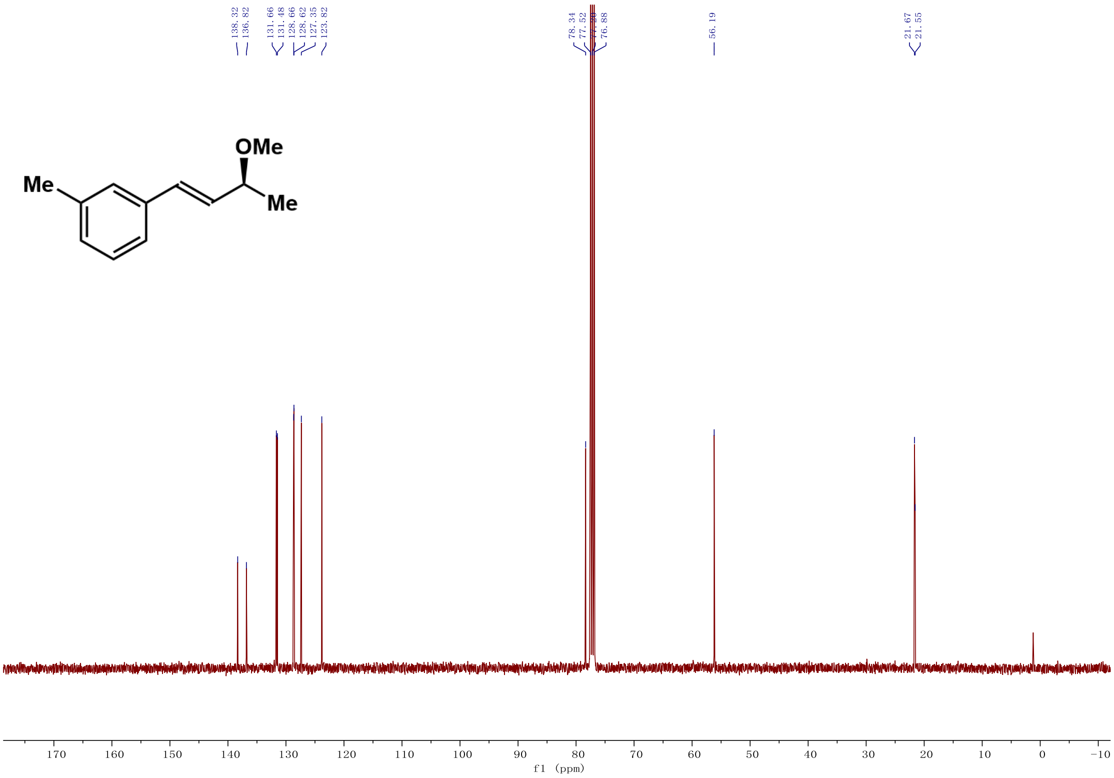

(*S*,*E*)-1-methoxy-2-(3-methoxybut-1-en-1-yl)benzene (3ha)

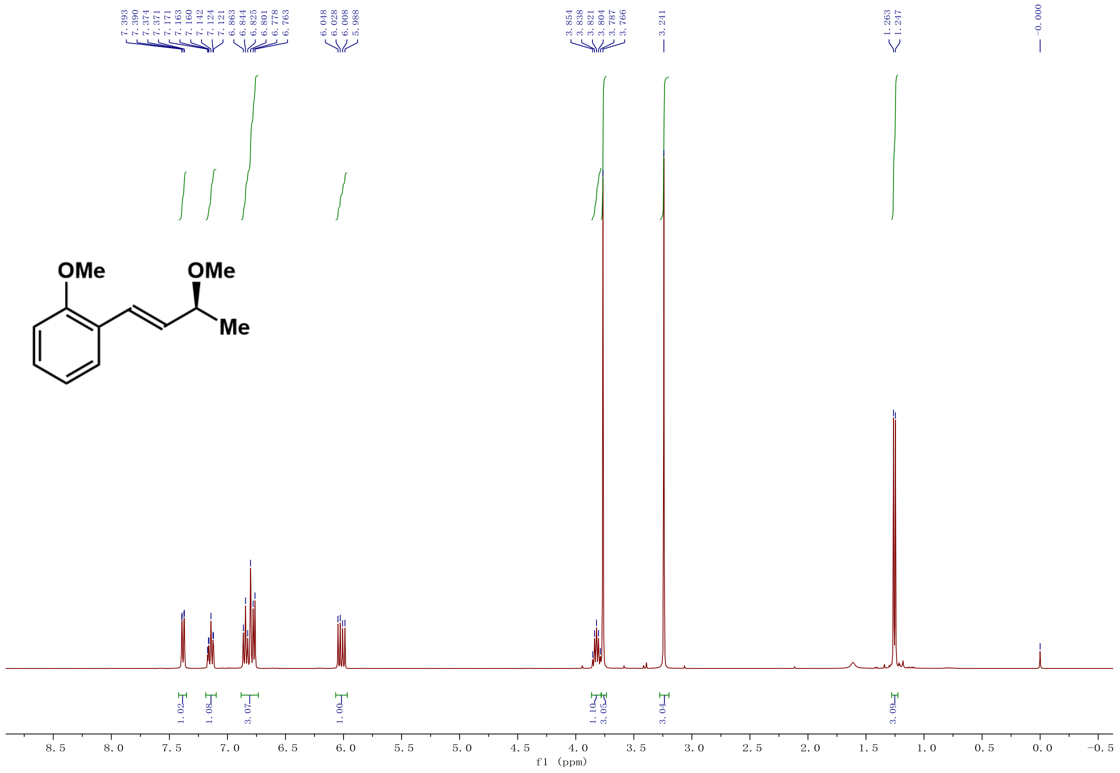

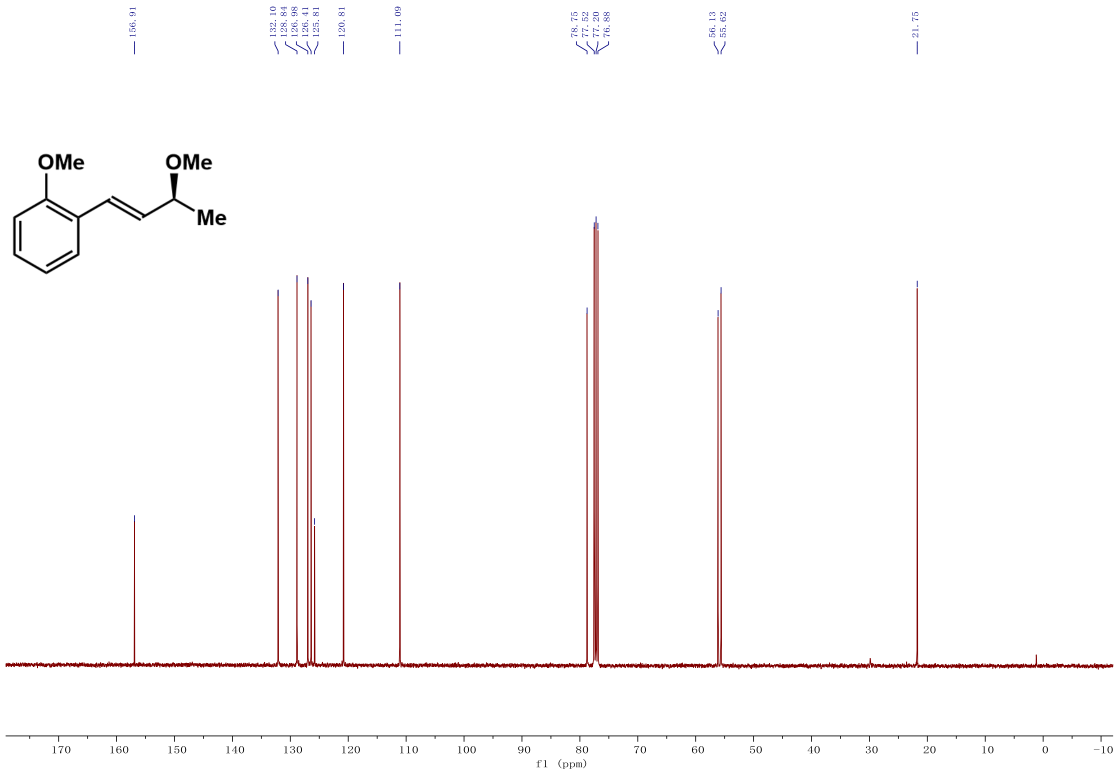

(*S*,*E*)-2-(3-methoxybut-1-en-1-yl)furan (3ia)

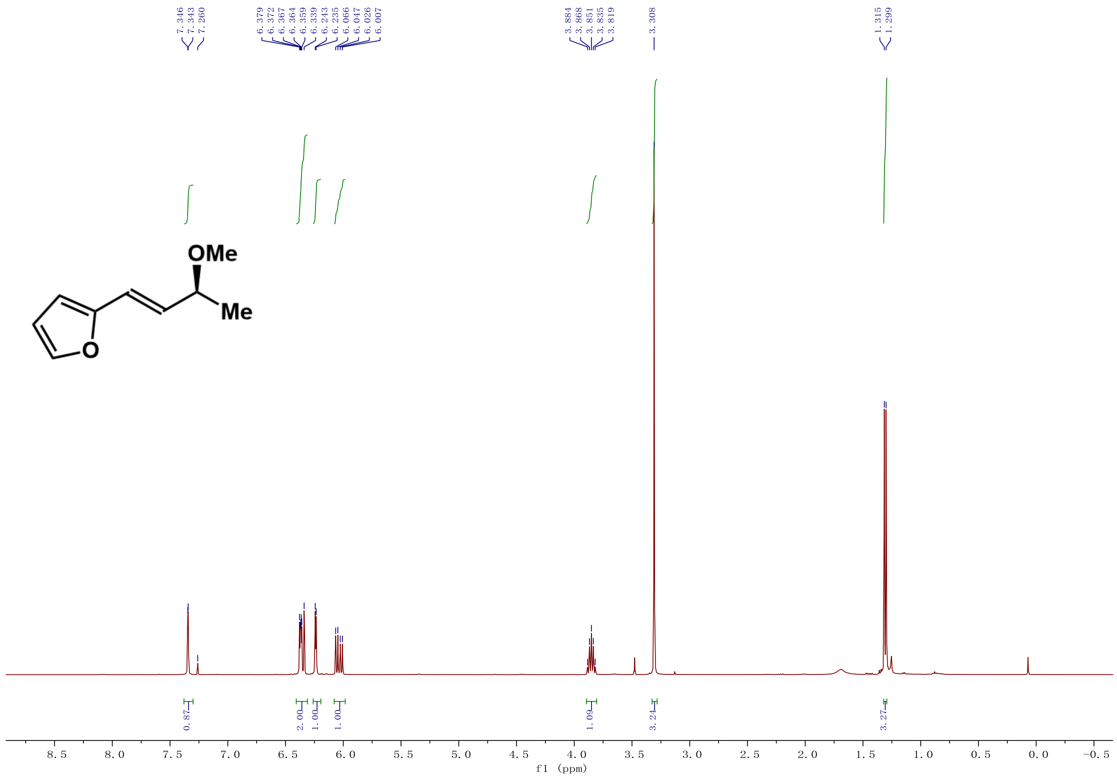

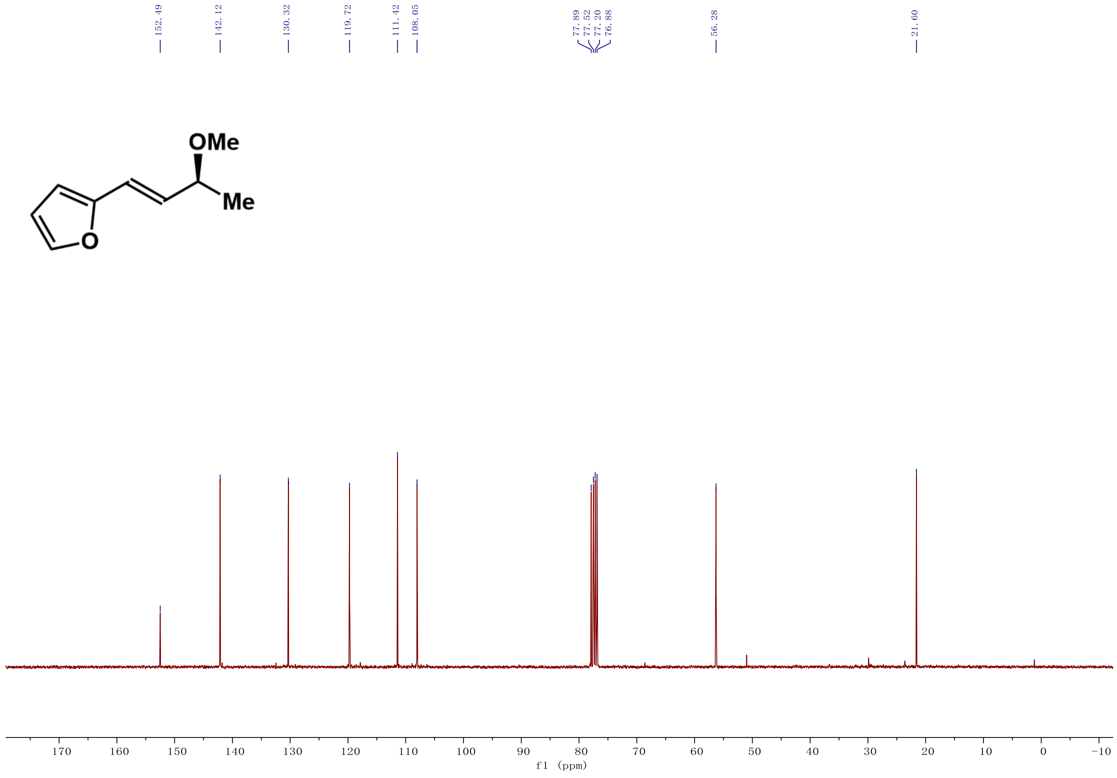

(*S*,*E*)-2-(3-methoxybut-1-en-1-yl)thiophene (3ja)

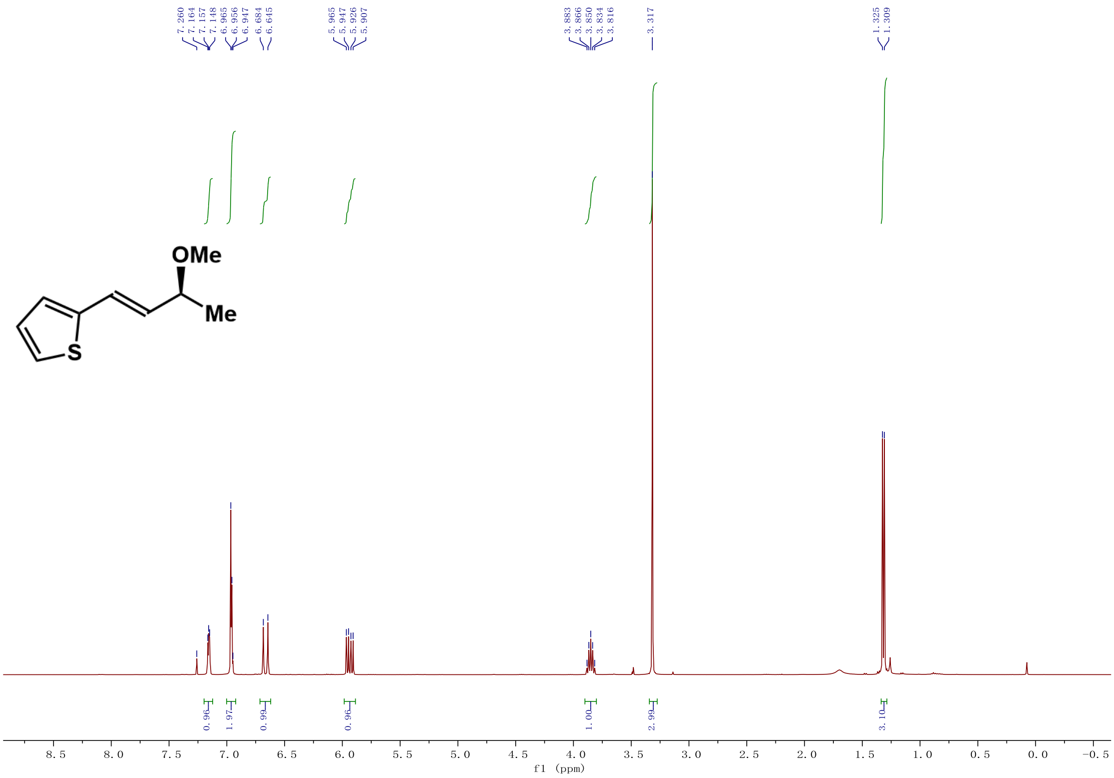

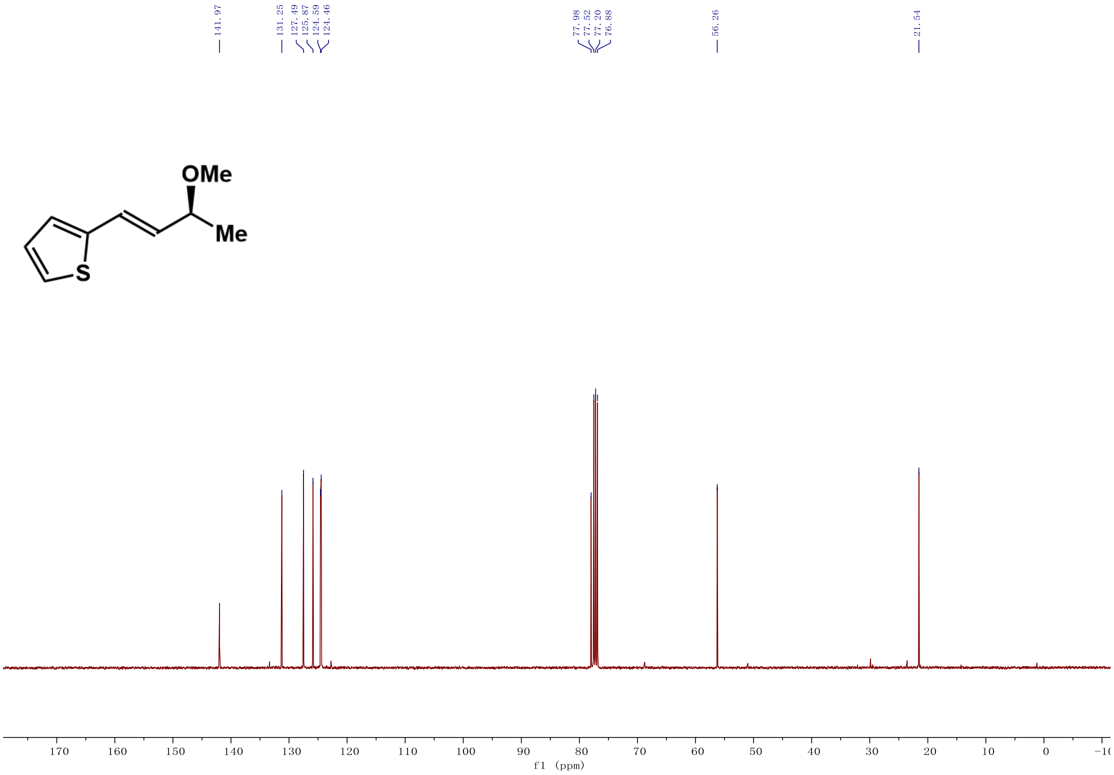

(*S*,*E*)-4-(((4-cyclohexylbut-3-en-2-yl)oxy)methyl)-1,1'-biphenyl (3ka)

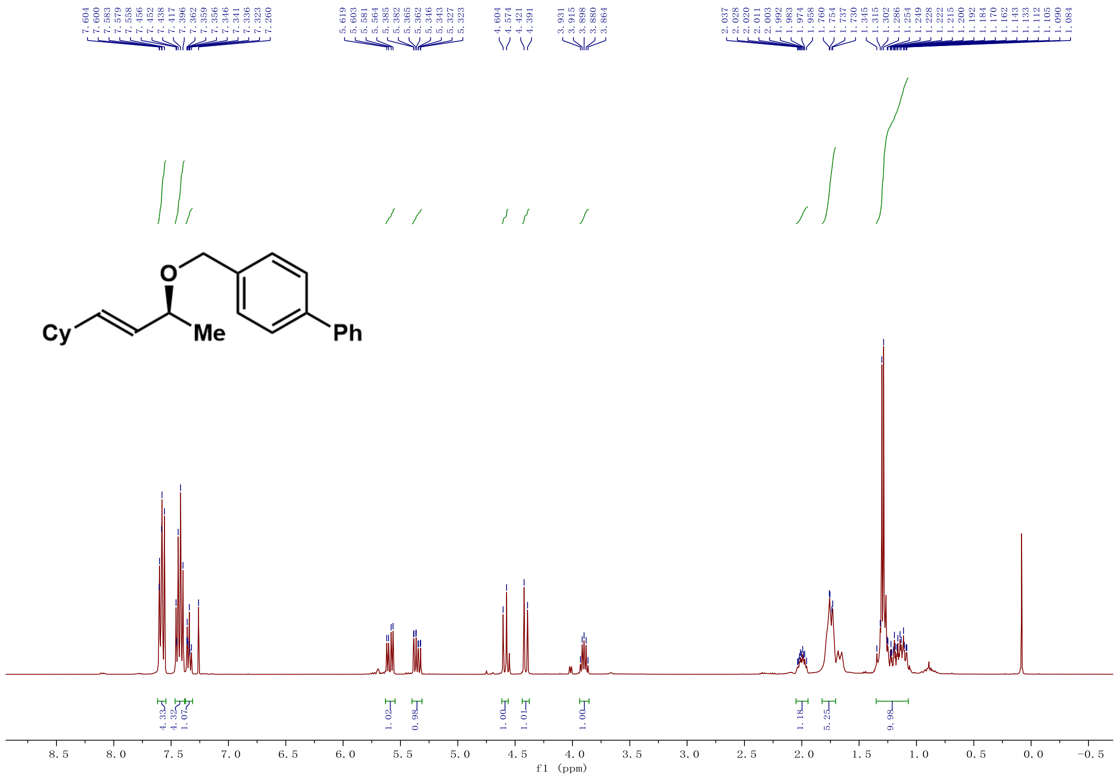

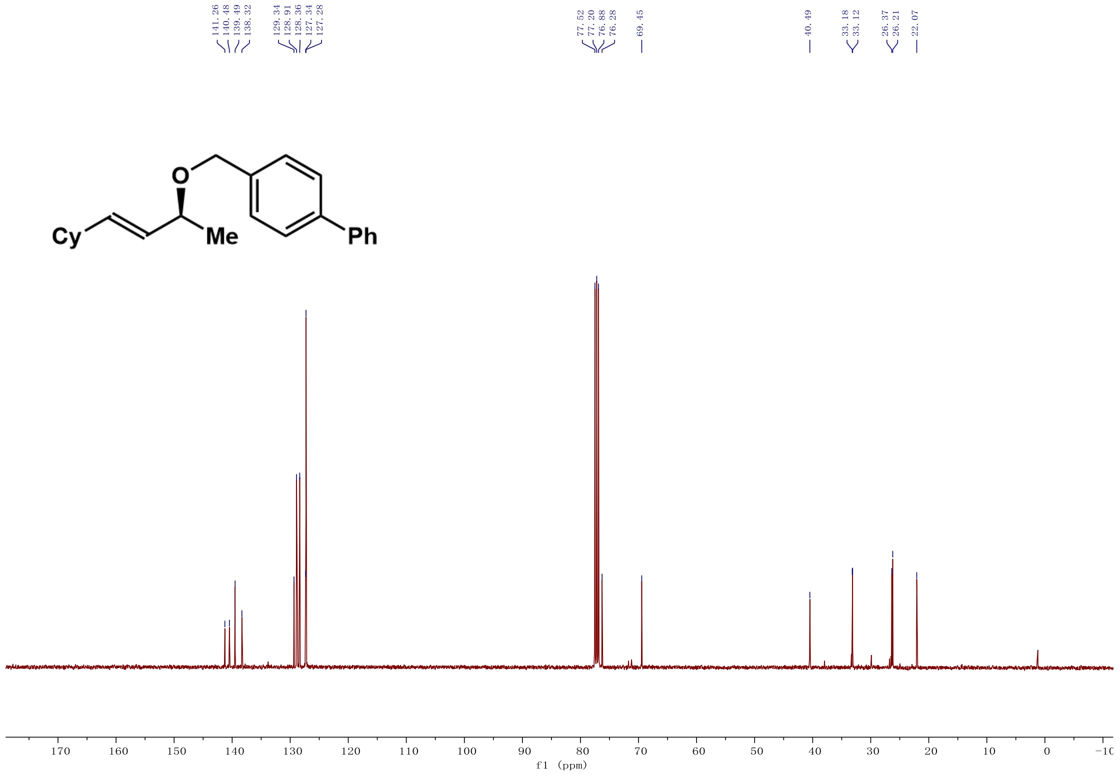

(*S*)-4-((but-3-en-2-yloxy)methyl)-1,1'-biphenyl (3la)

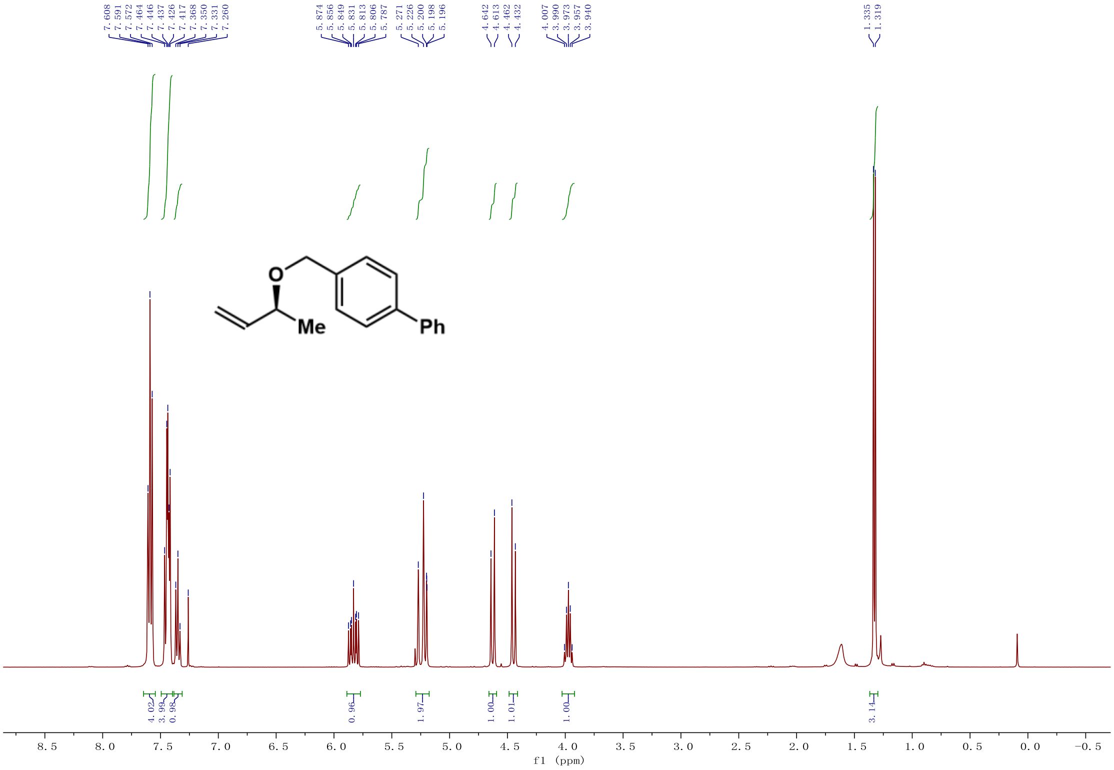

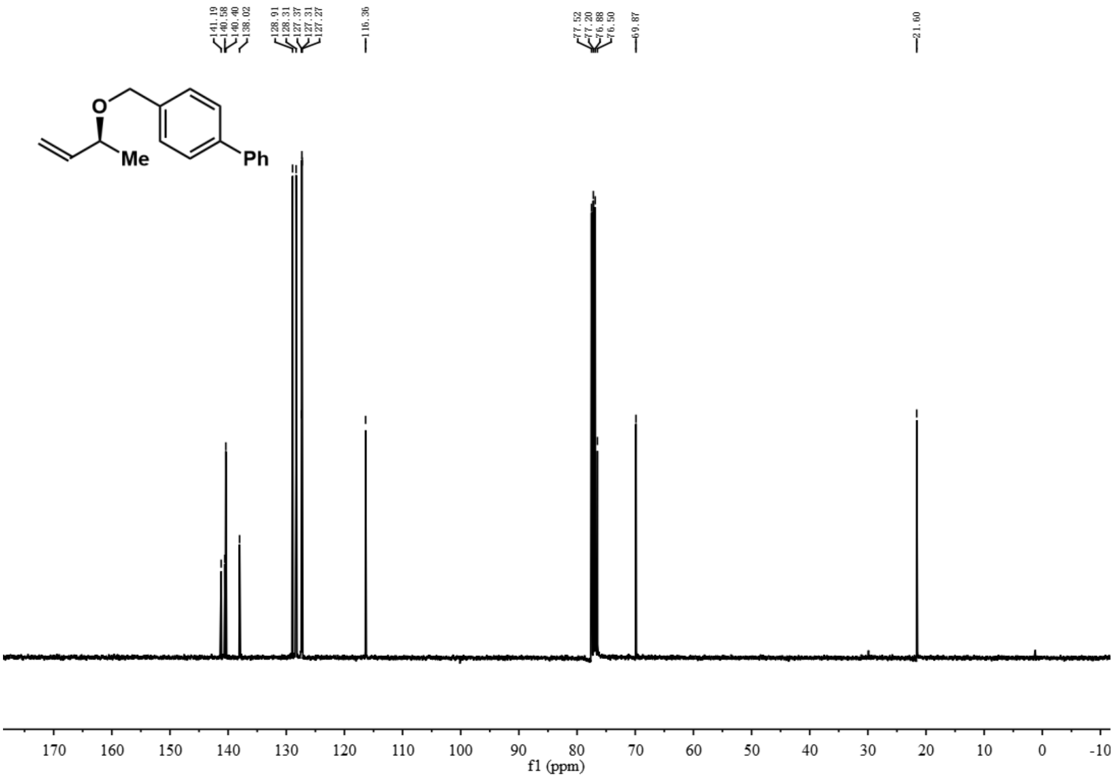

(S)-(3-methoxybut-1-en-2-yl)benzene (3ma)

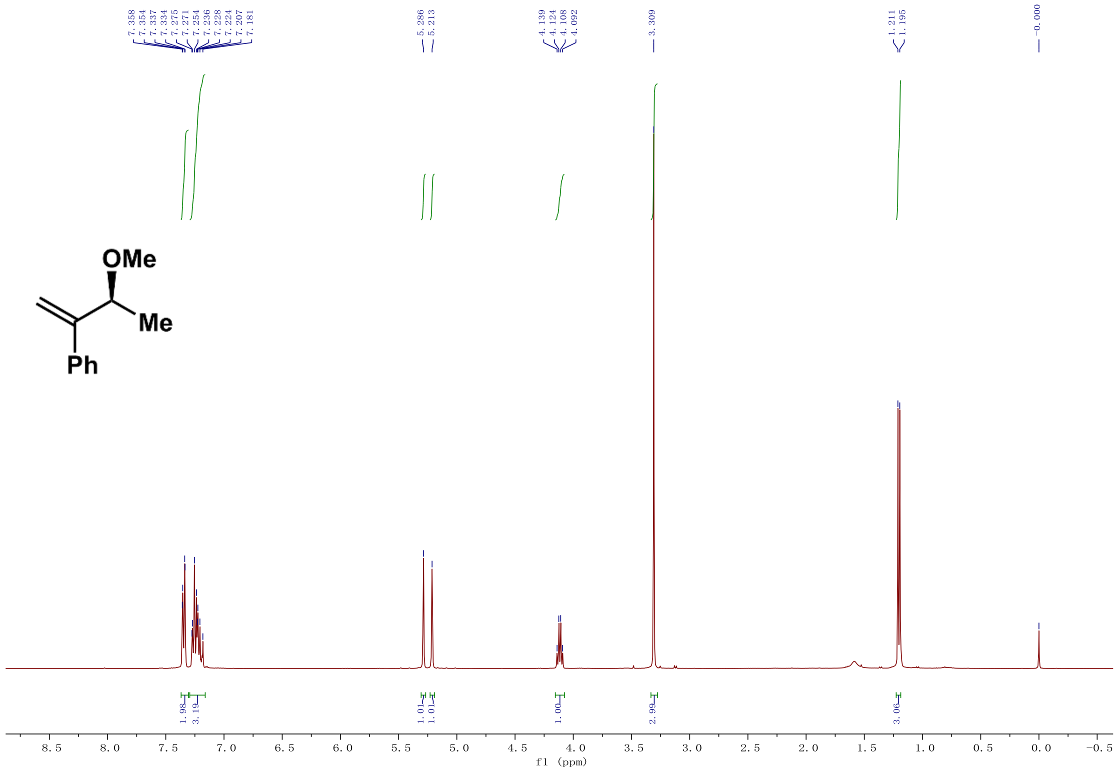

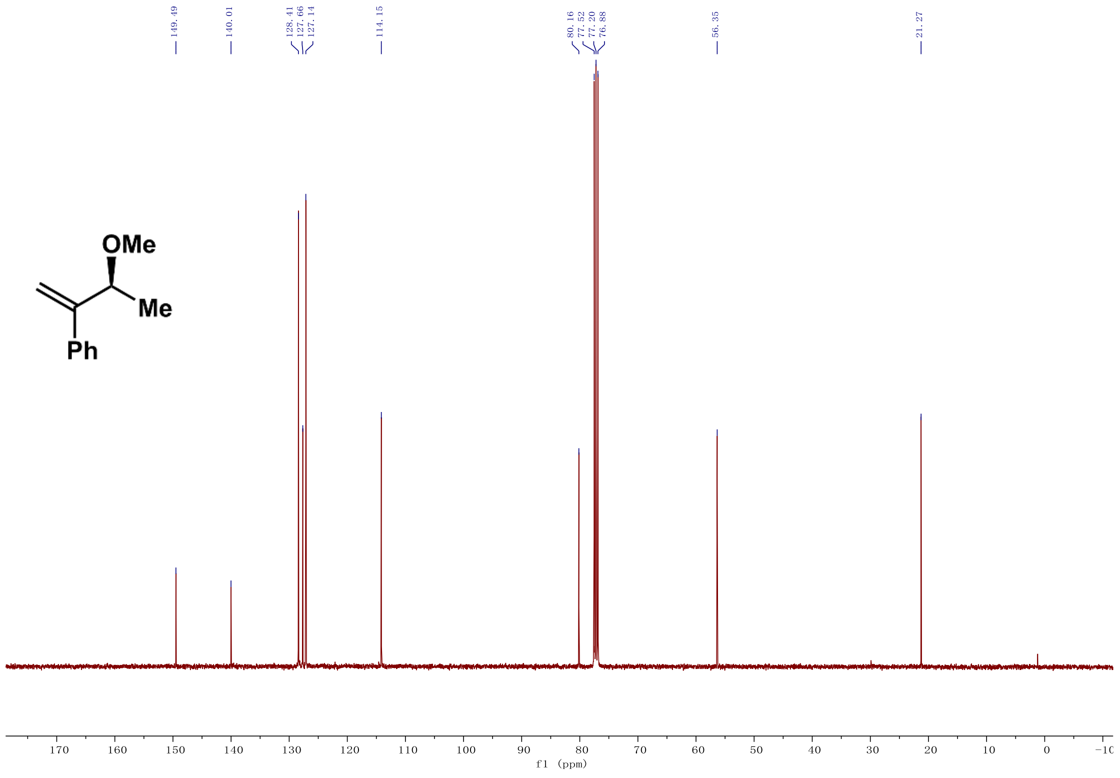

(*S*,*E*)-(3-ethoxybut-1-en-1-yl)benzene (3ab)

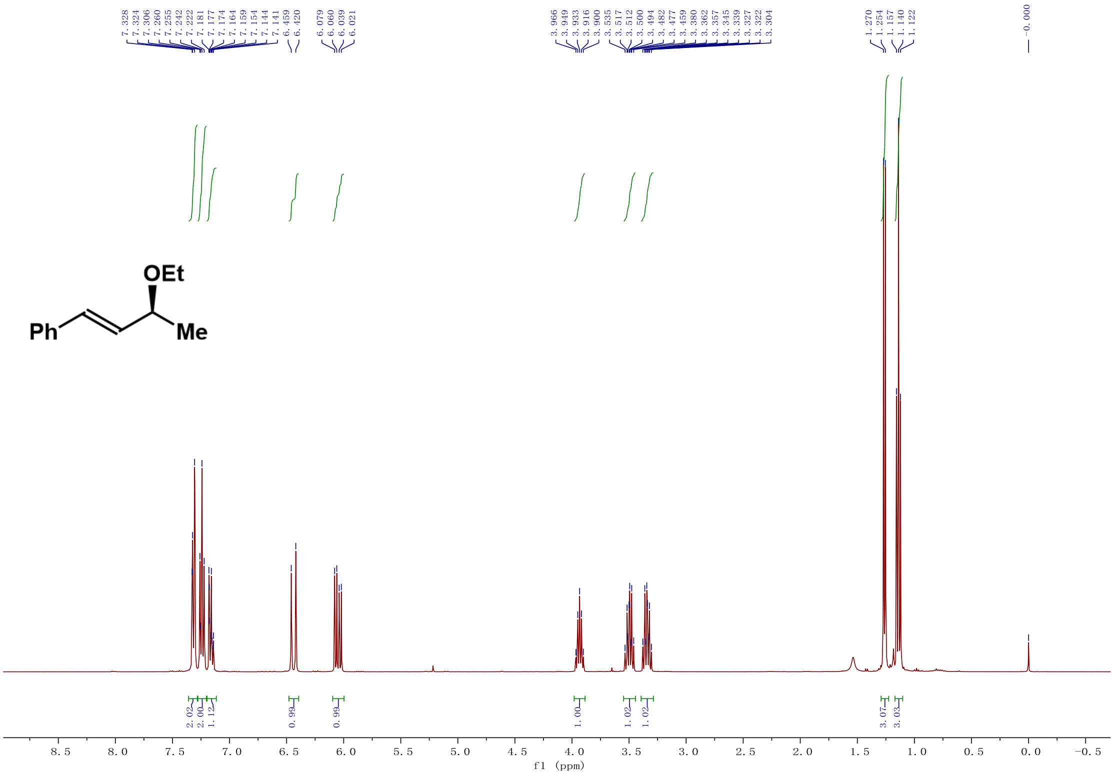

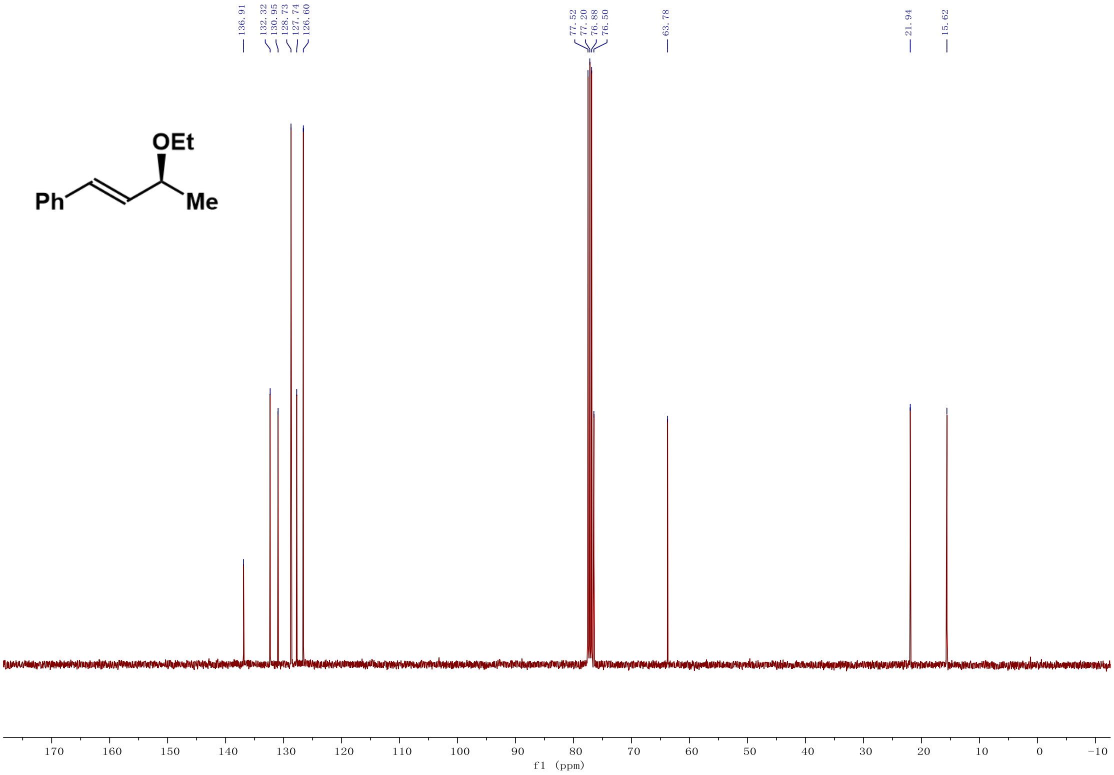

(*S*,*E*)-(3-(benzyloxy)but-1-en-1-yl)benzene (3ac)

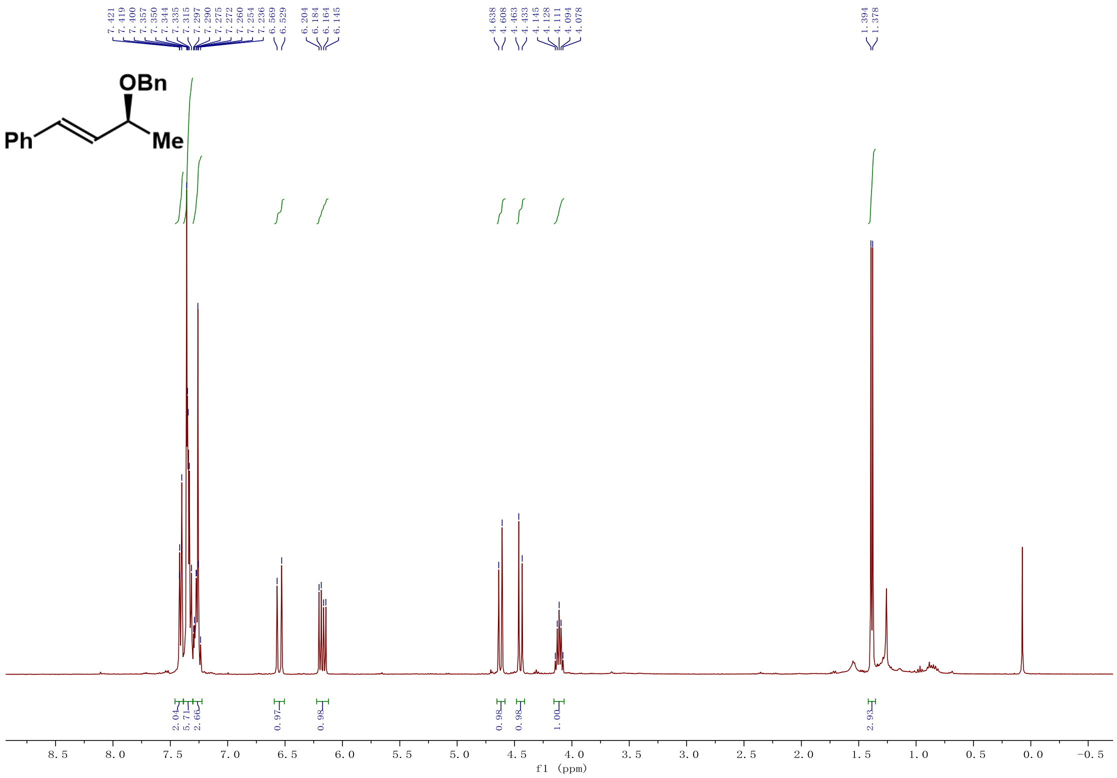

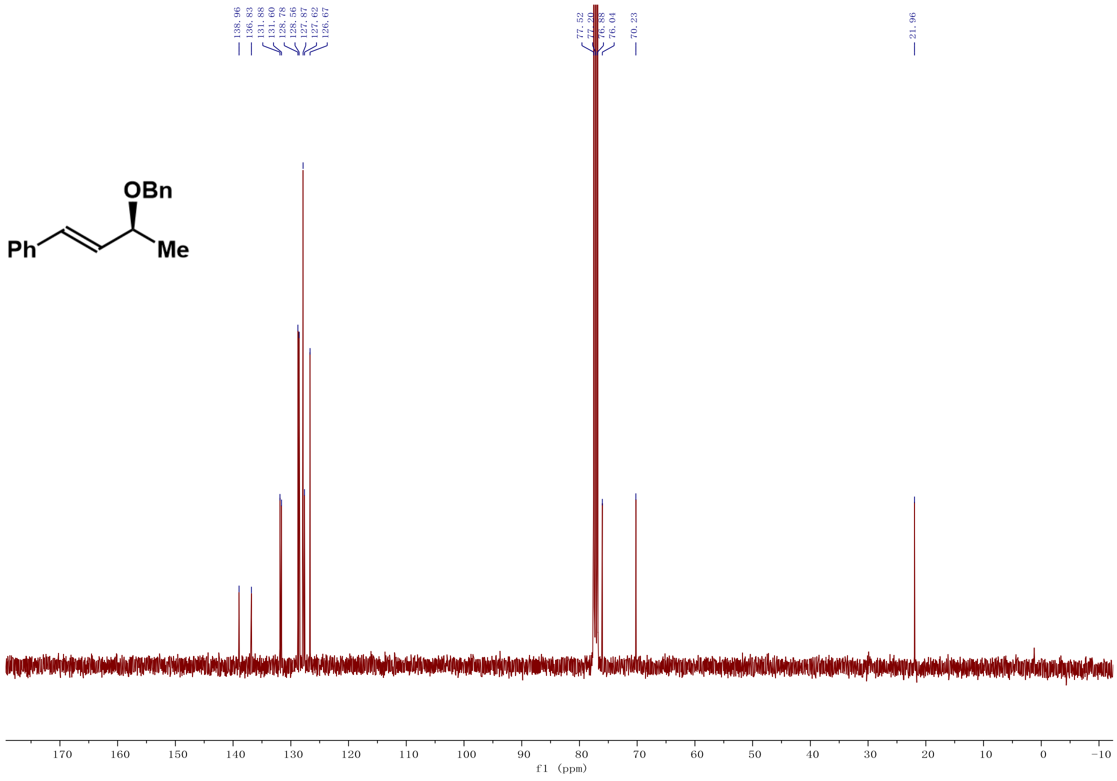

(*S*,*E*)-(3-((4-phenylbut-3-en-2-yl)oxy)propyl)benzene (3ad)

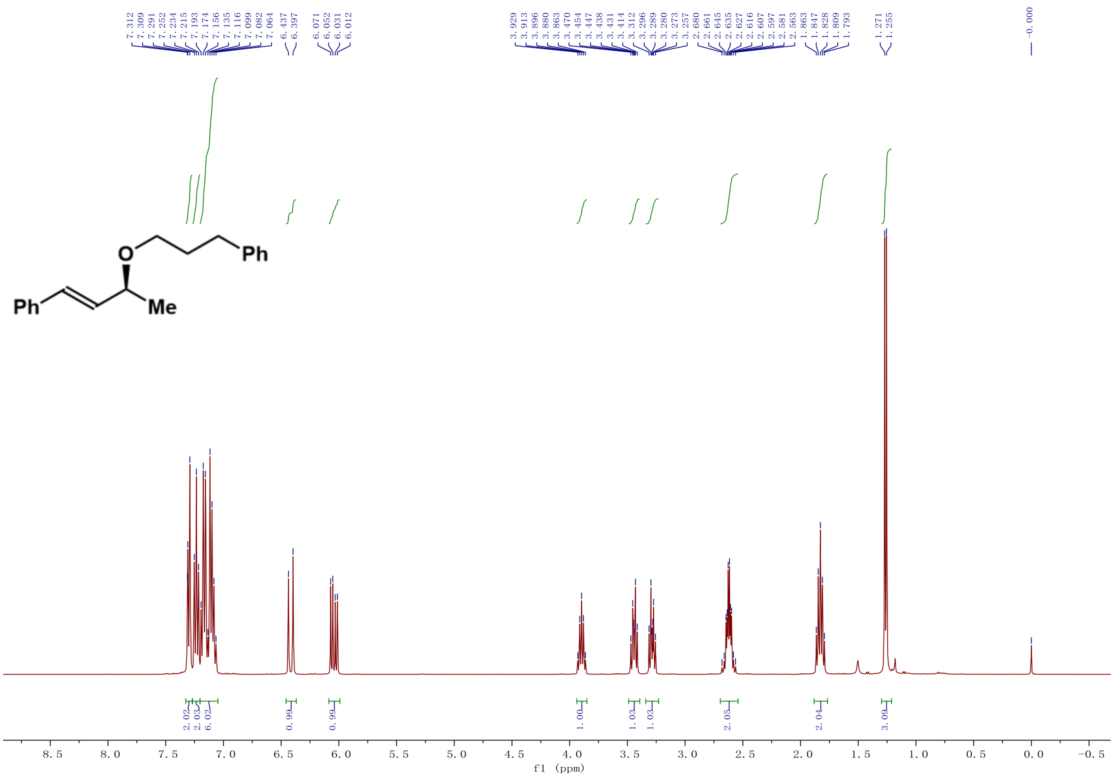

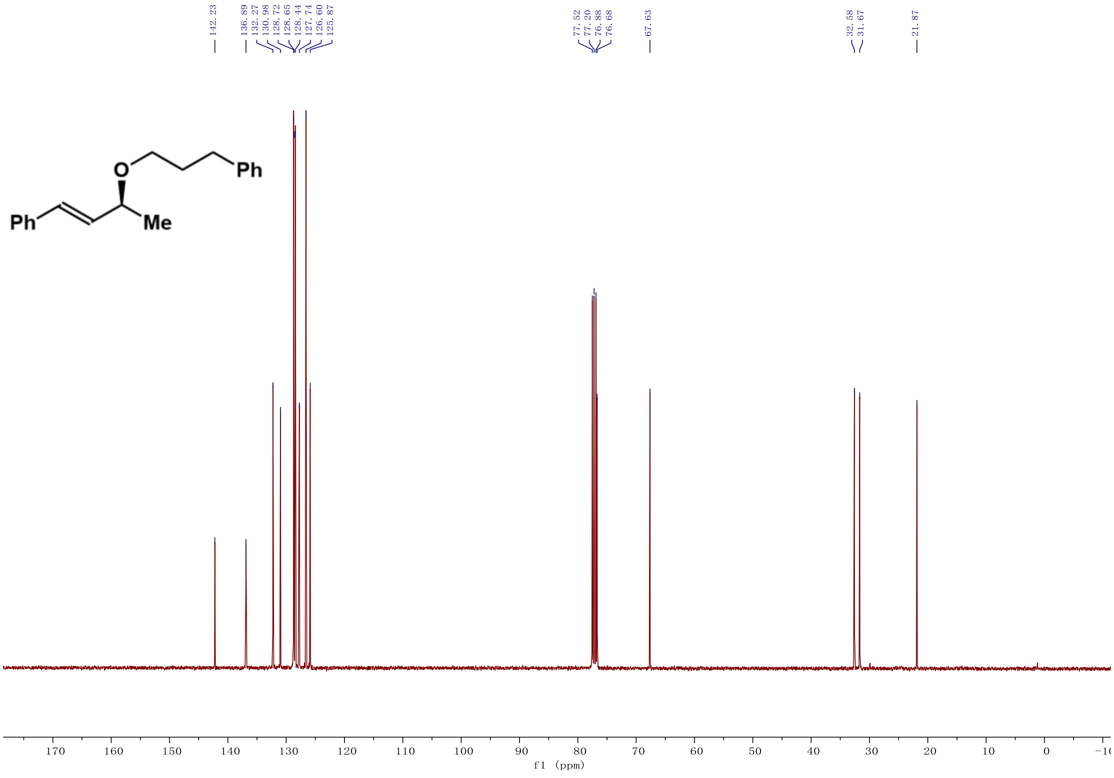

(*S*,*E*)-(3-(3-chloropropoxy)but-1-en-1-yl)benzene (3ae)

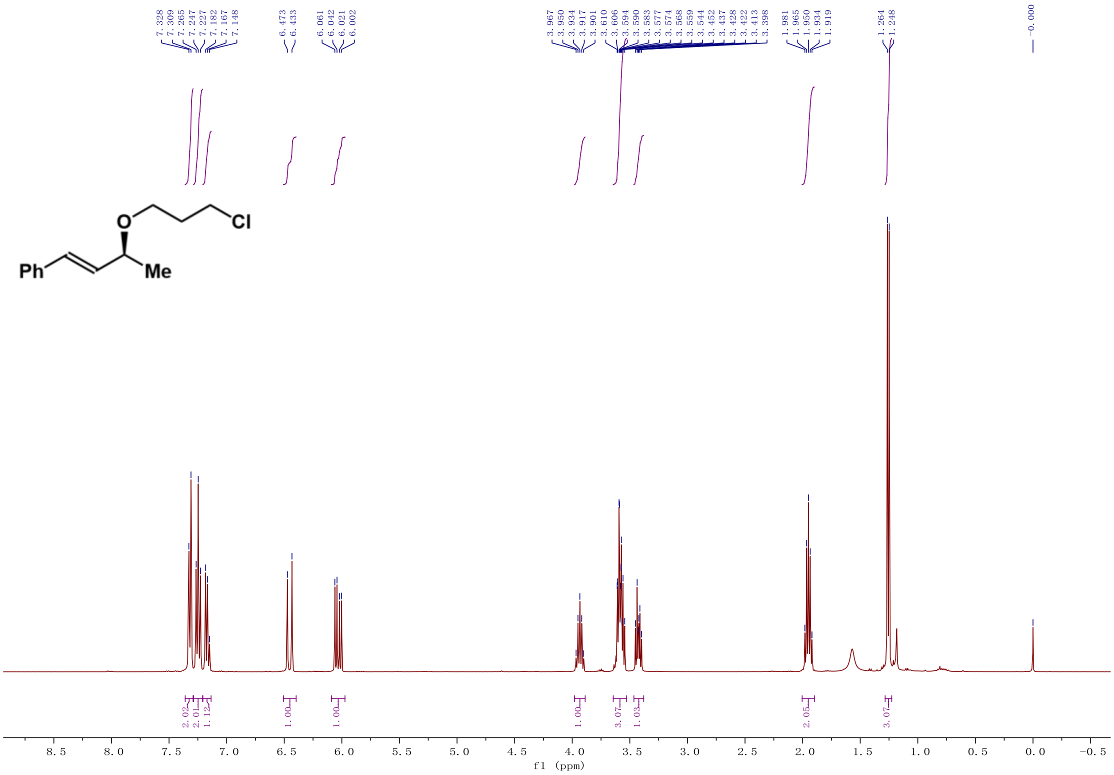

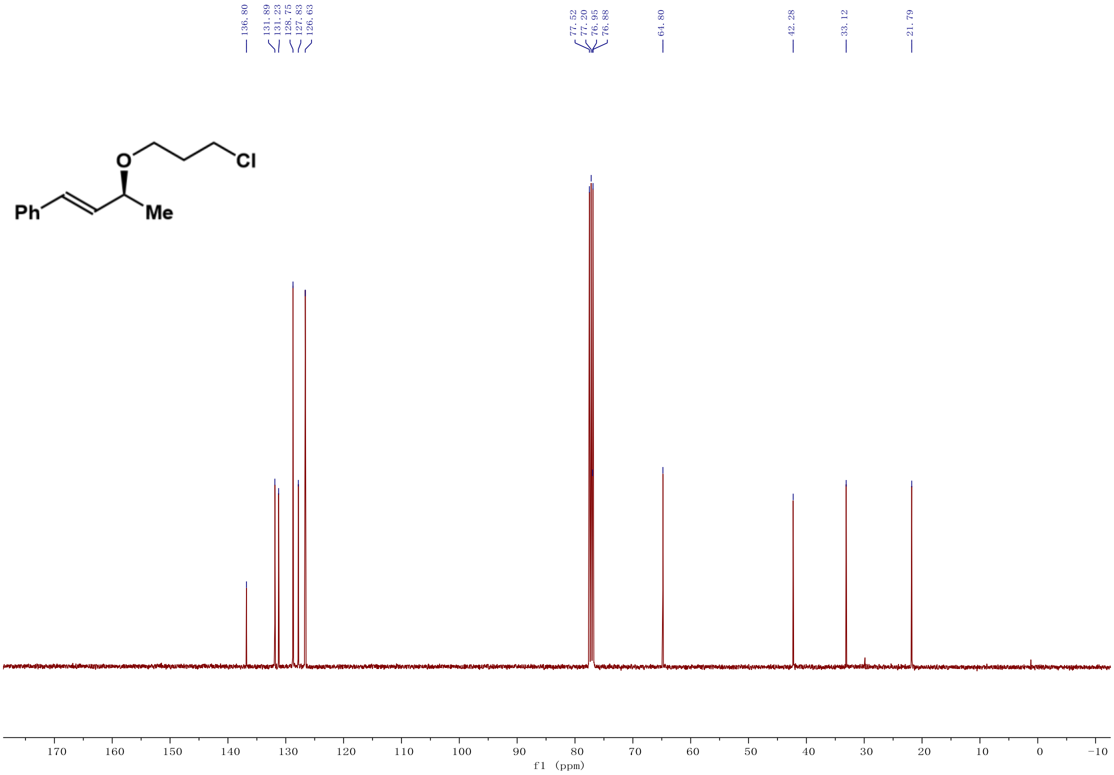

(*S,E*)-trimethyl(3-((4-phenylbut-3-en-2-yl)oxy)propyl)silane (3af)

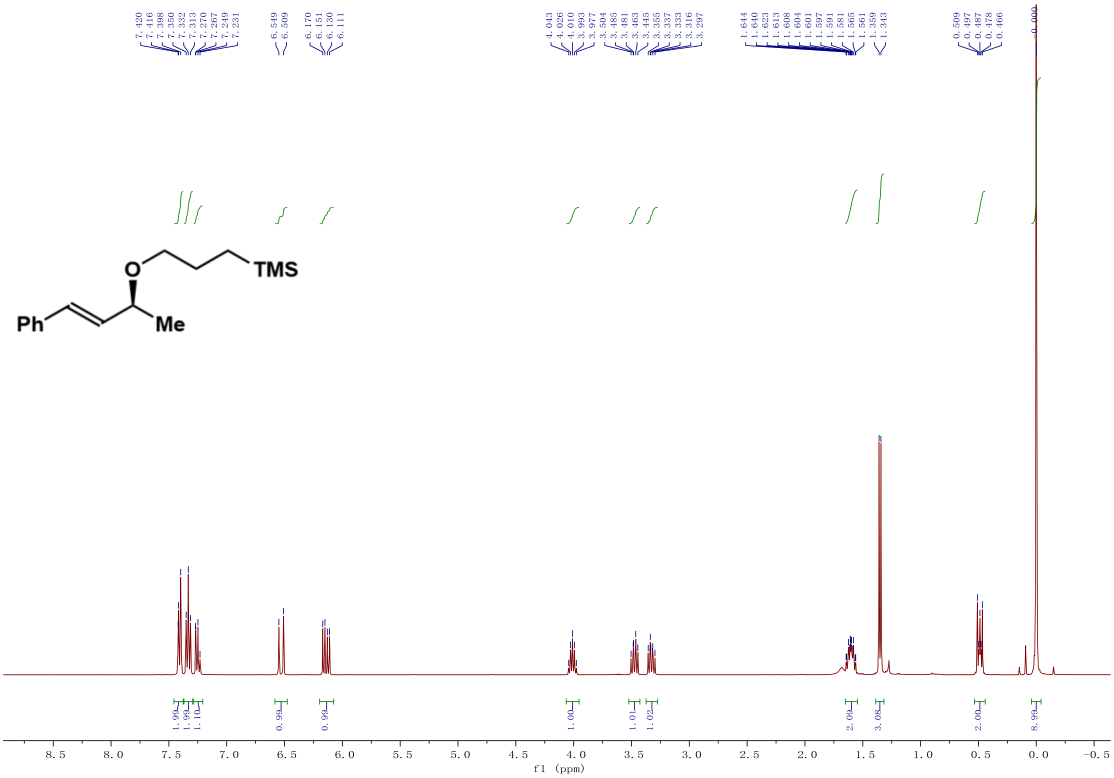

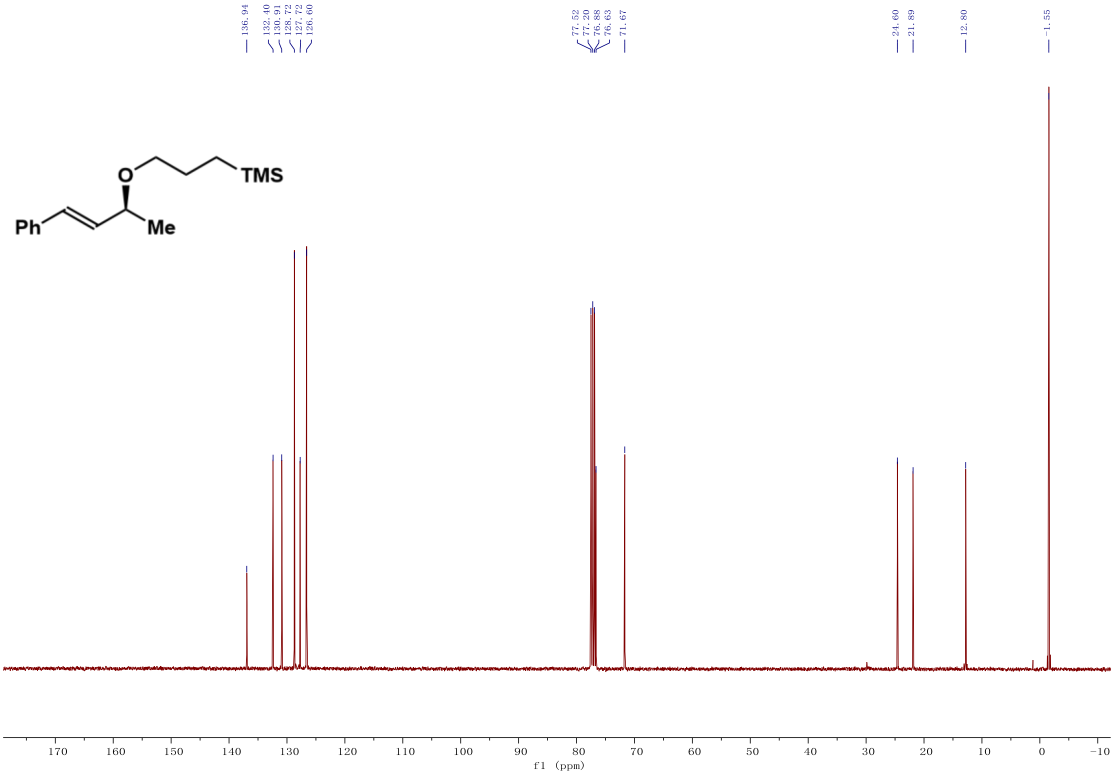

((*S*,*E*)-3-(cinnamyloxy)but-1-en-1-yl)benzene (3ag)

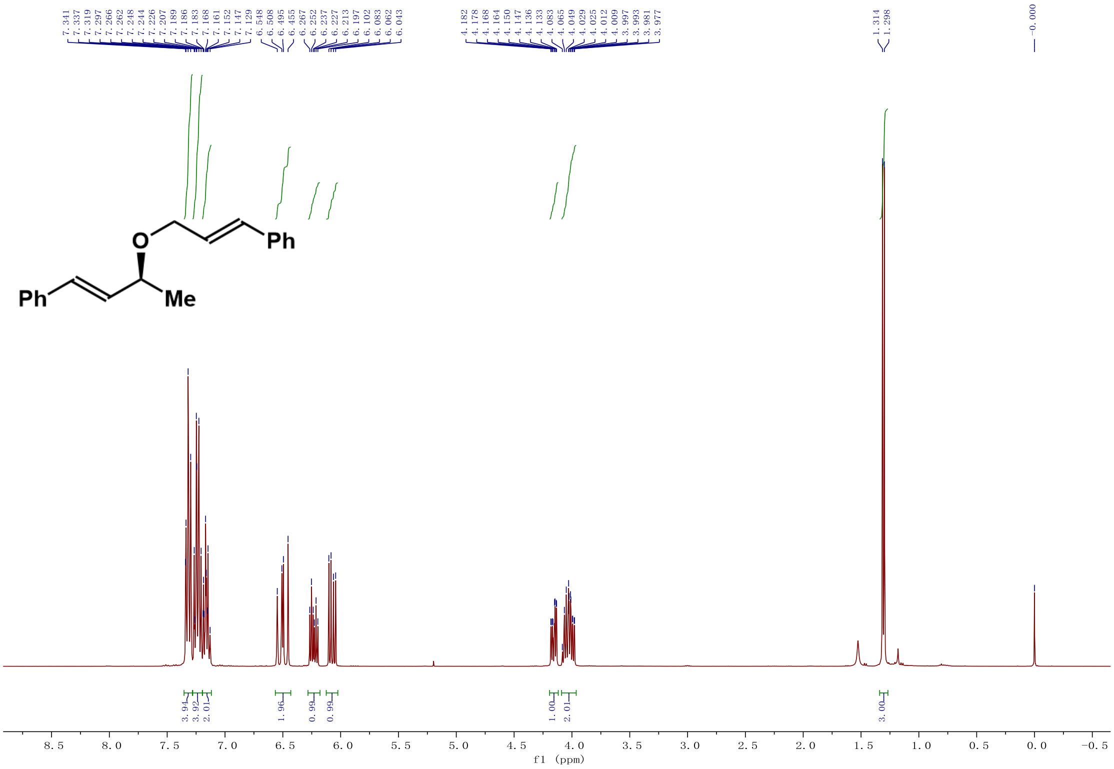

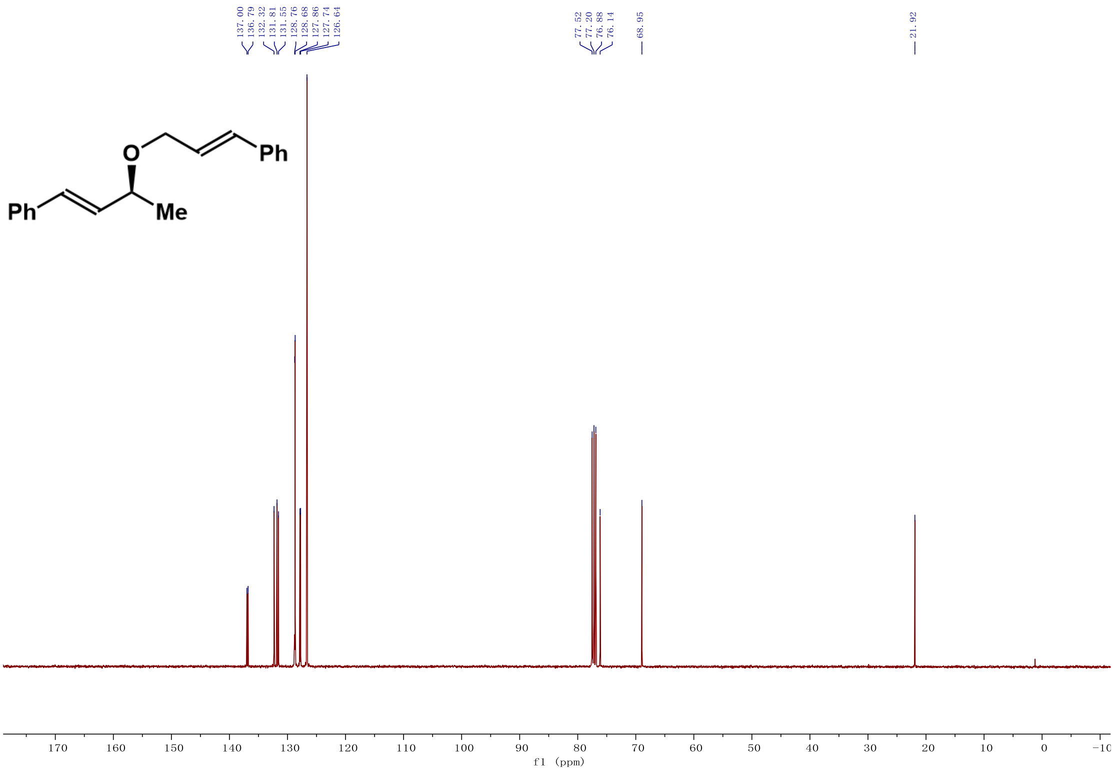

(*S*,*E*)-1-fluoro-4-(((4-phenylbut-3-en-2-yl)oxy)methyl)benzene (3ah)

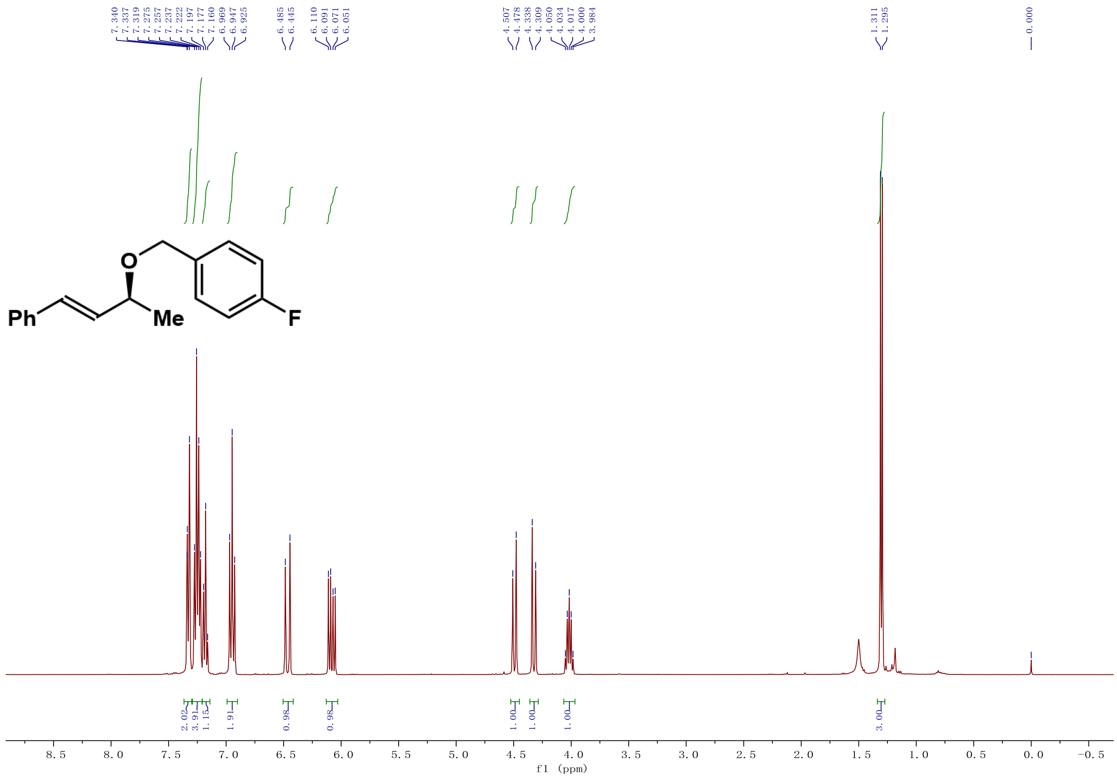

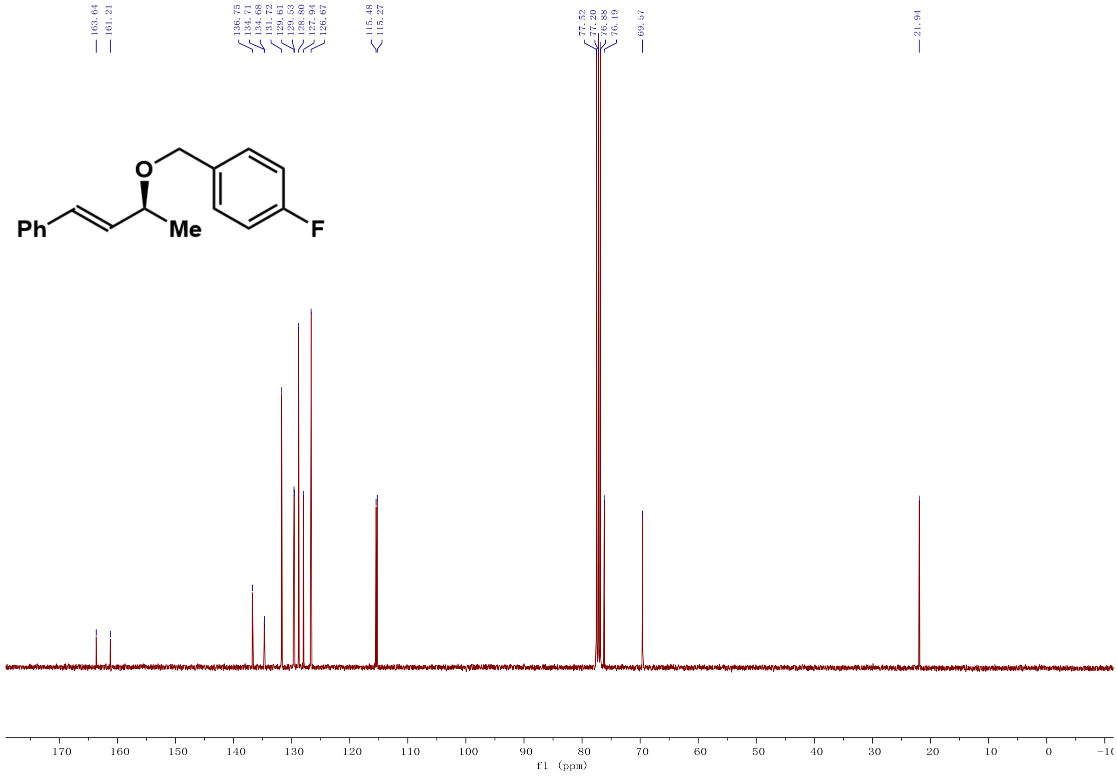

(*S*,*E*)-1-methyl-4-(((4-phenylbut-3-en-2-yl)oxy)methyl)benzene (3ai)

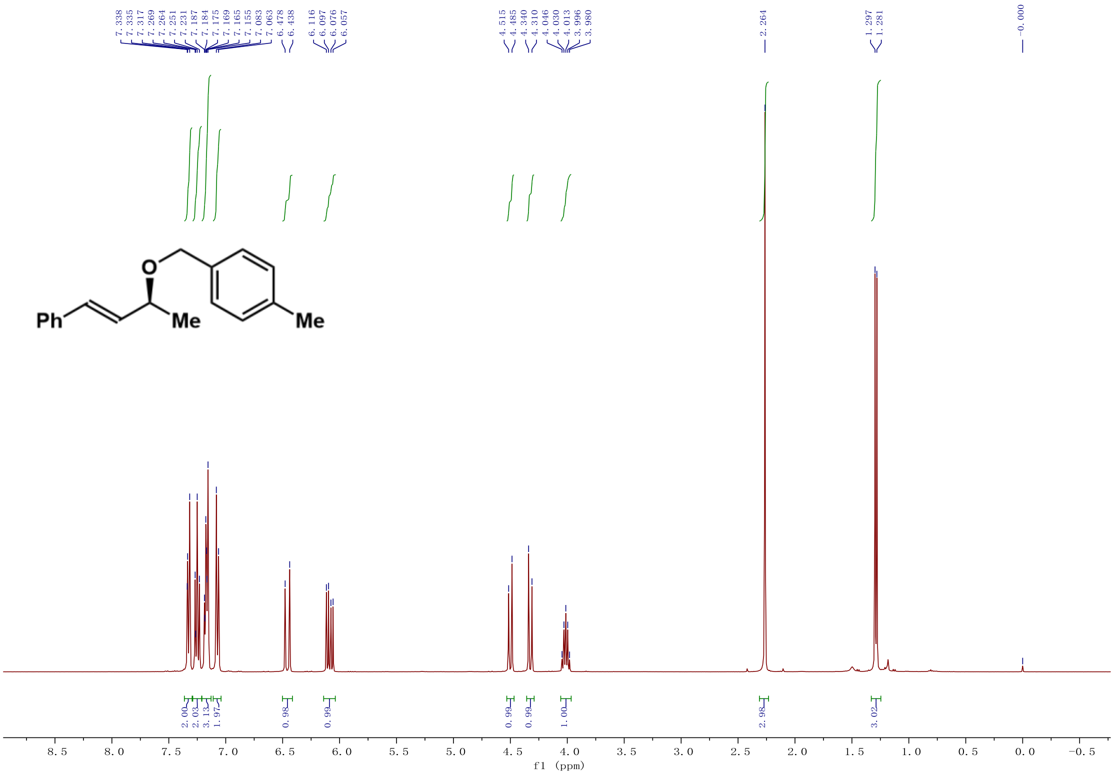

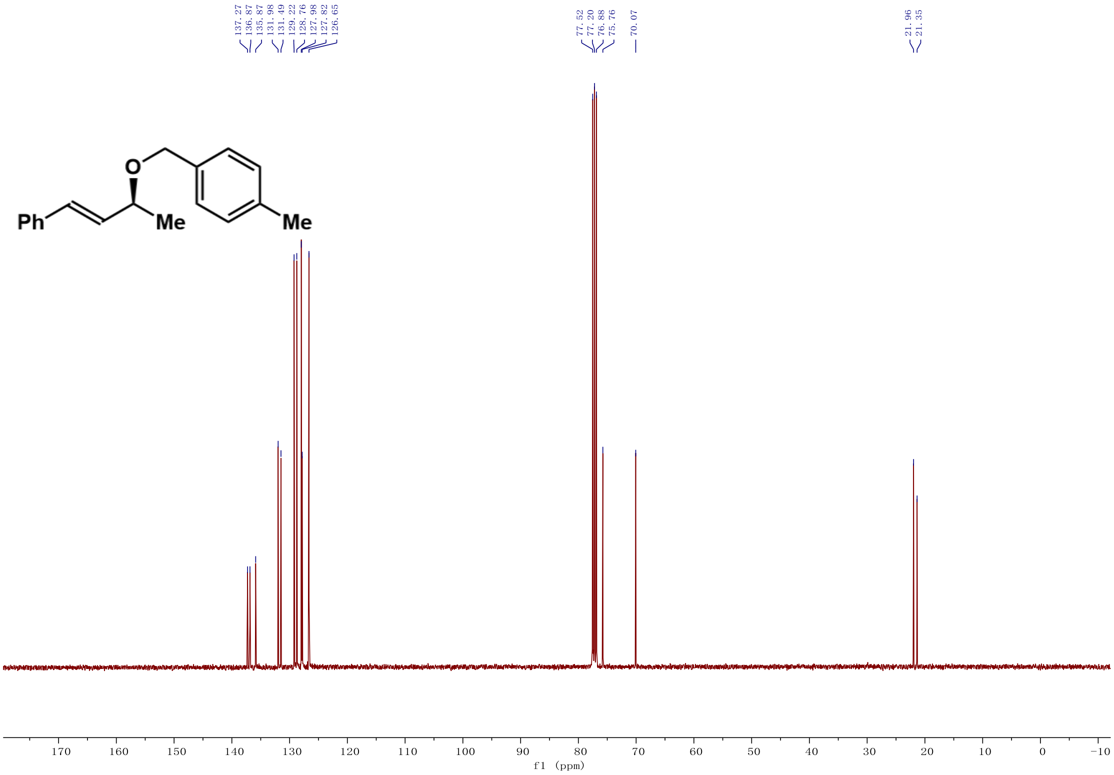

(*S*,*E*)-1-methoxy-4-(((4-phenylbut-3-en-2-yl)oxy)methyl)benzene (3aj)

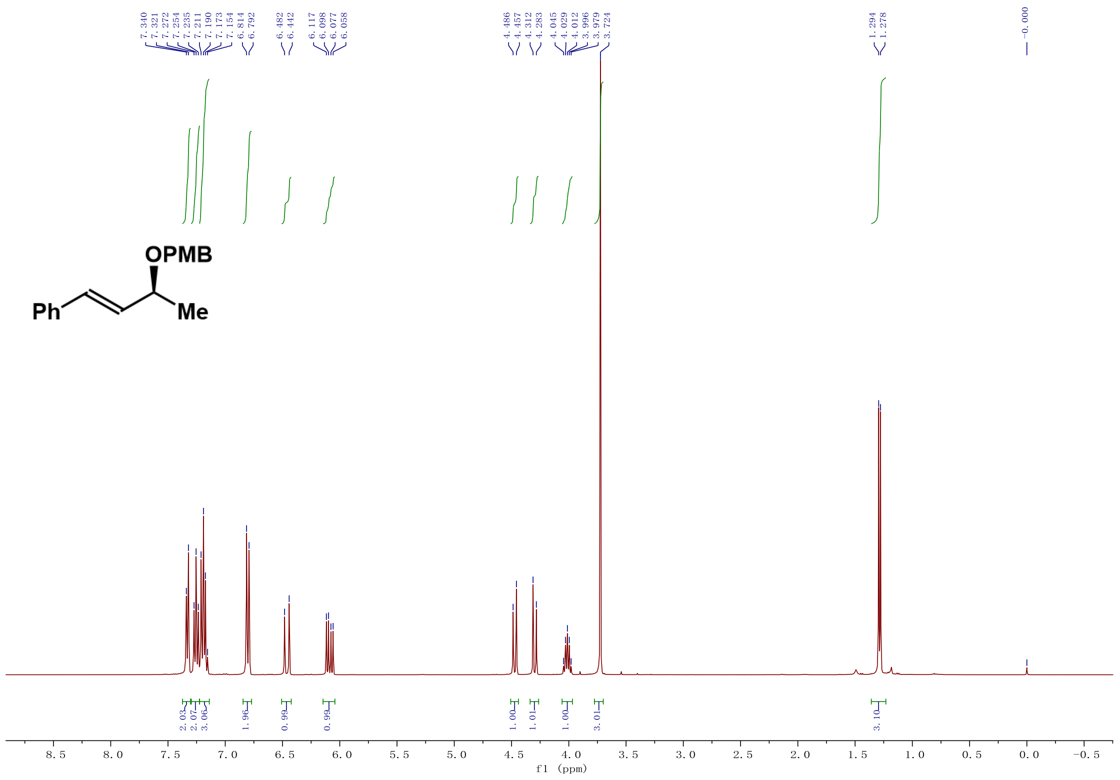

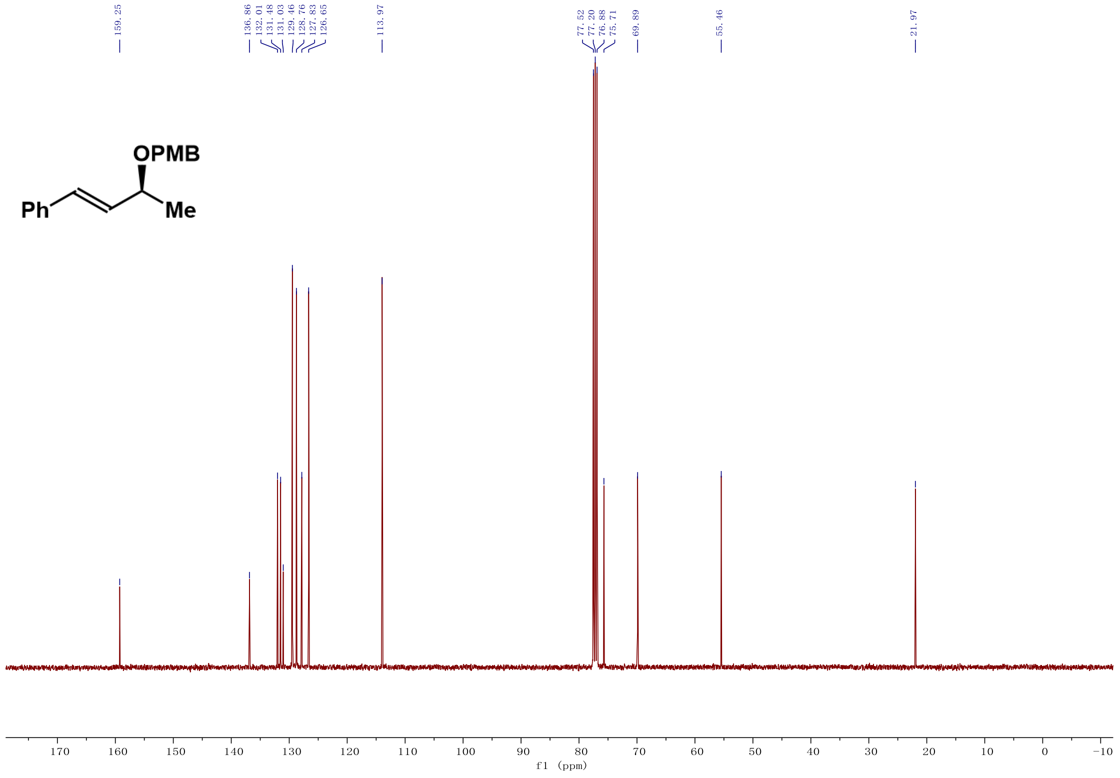

((*S*,*E*)-3-(((*S*)-3,7-dimethyloct-6-en-1-yl)oxy)but-1-en-1-yl)benzene (3ak)

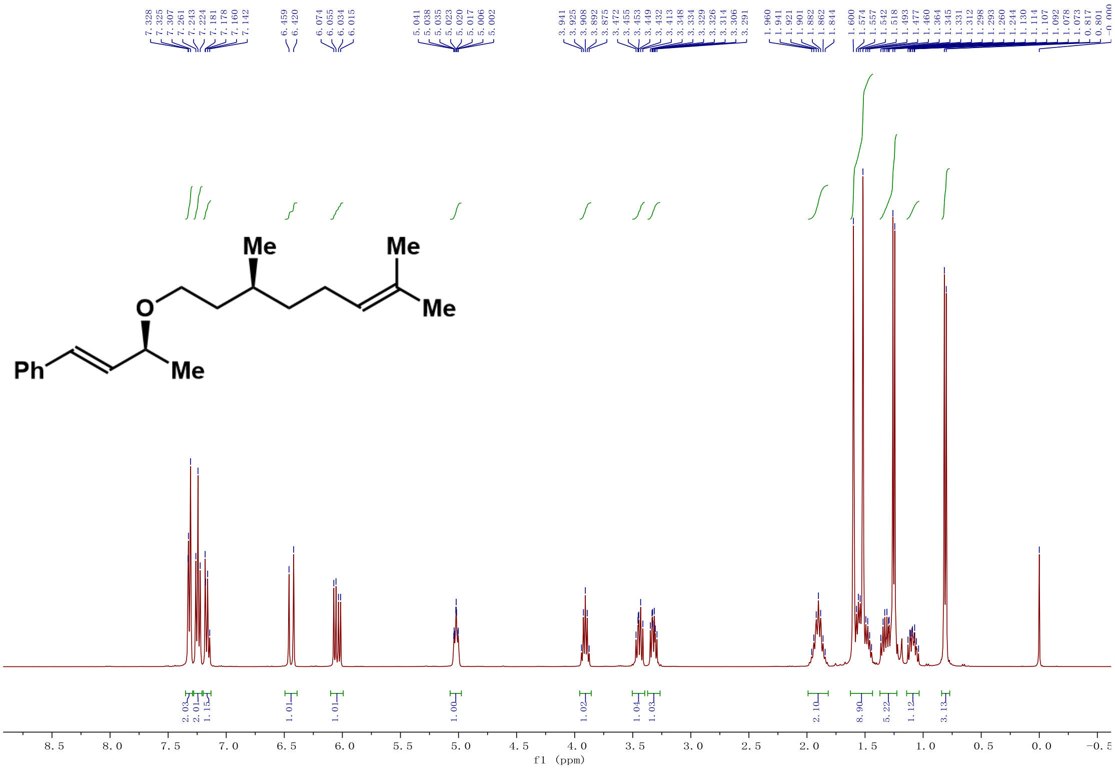

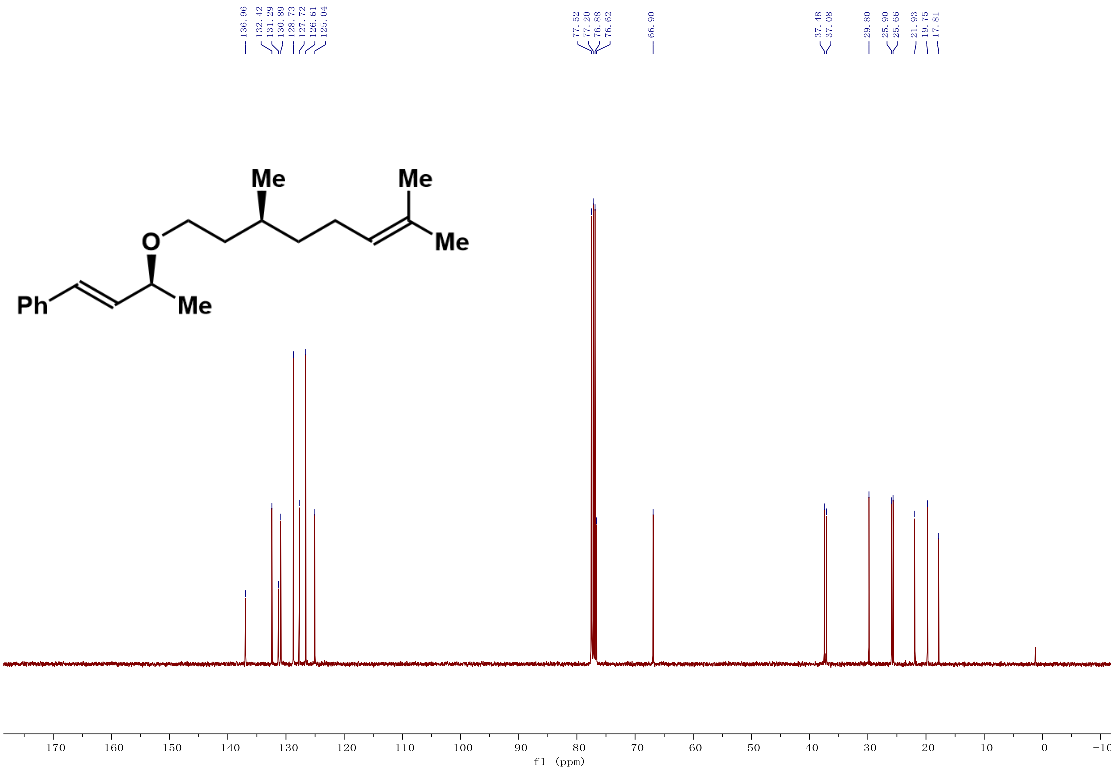

(*S*,*E*)-(3-cyclopropoxybut-1-en-1-yl)benzene (3al)

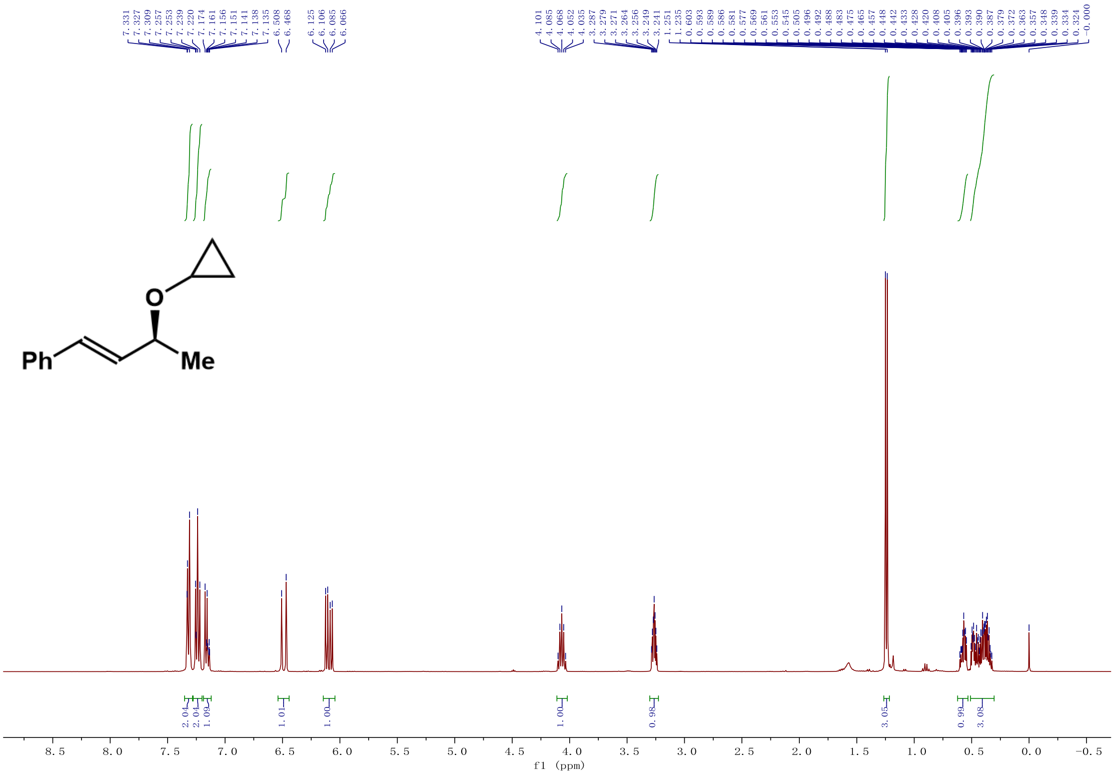

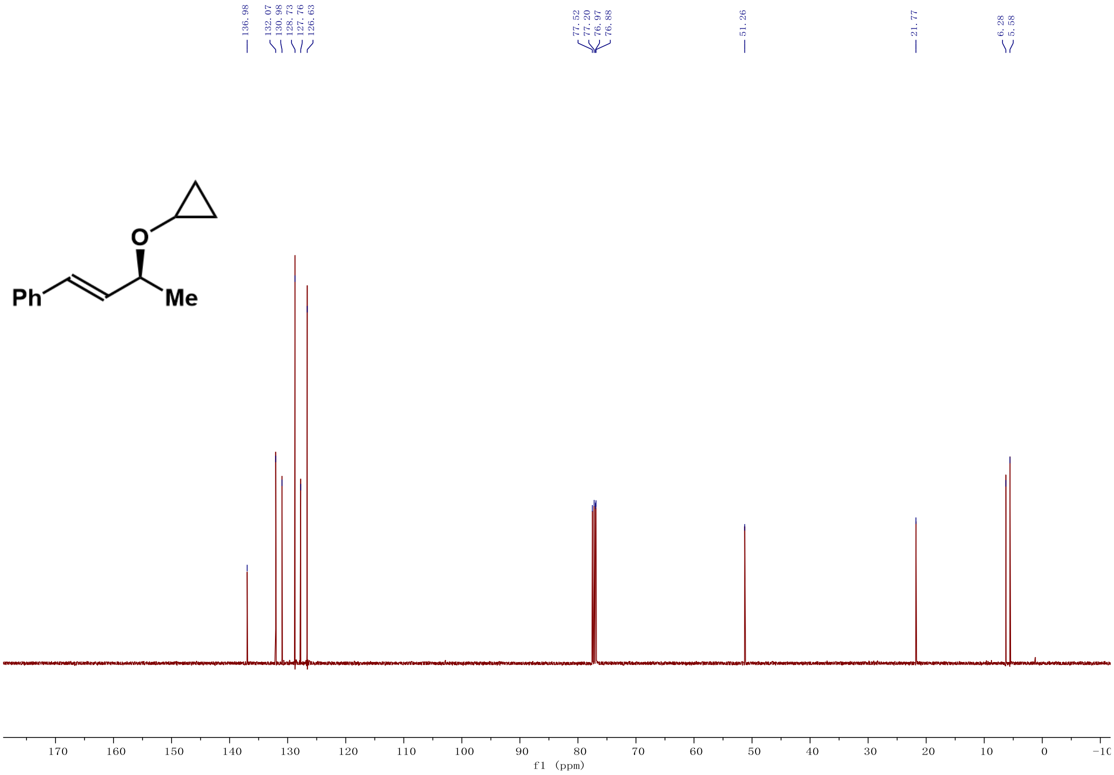

(*S,E*)-(3-(cyclopentyloxy)but-1-en-1-yl)benzene (3am)

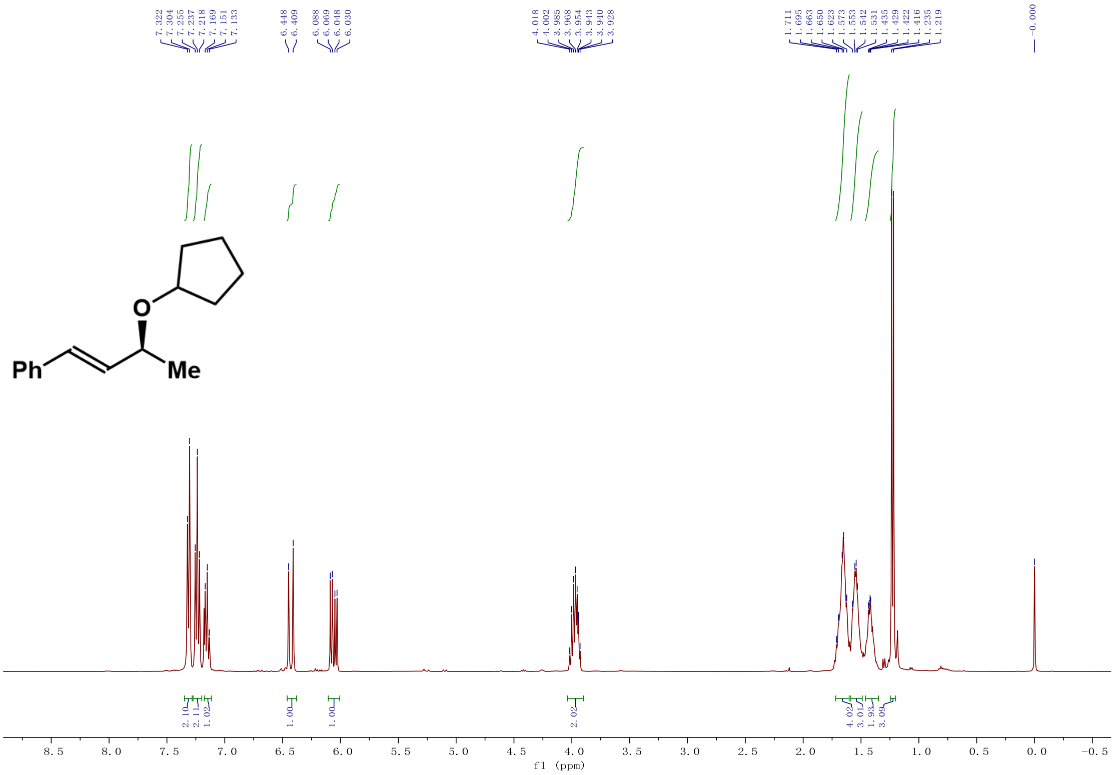

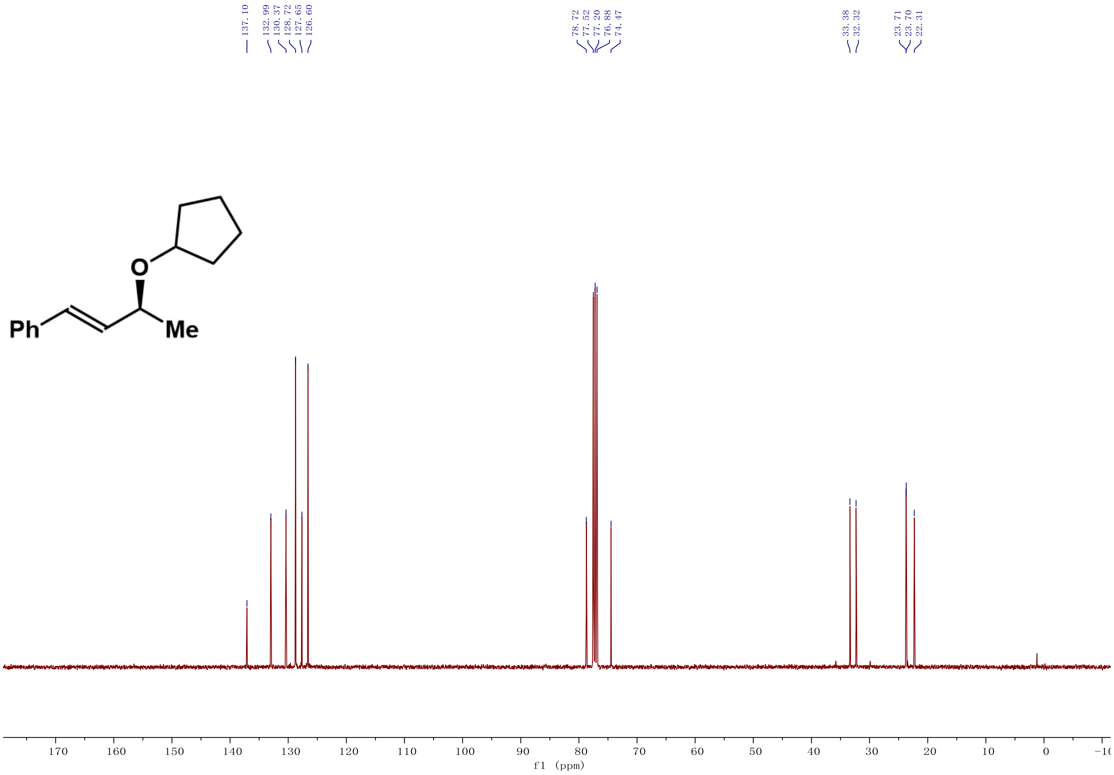

7. HPLC spectra

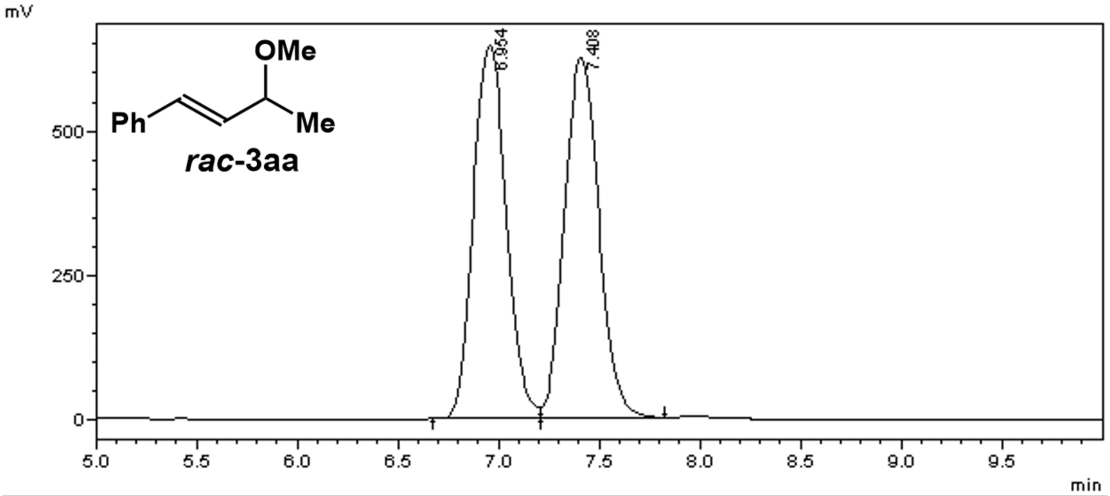

峰表

检测器 A Ch1 254nm

峰#	保留时间	面积	高度	面积 %
1	6.954	7361837	646214	49.697
2	7.408	7451494	624049	50.303

Translation of Chinese characters to English:

Peak	Retention time	Area	Height	Area %
1	6.954	7361837	646214	49.697
2	7.408	7451494	624049	50.303

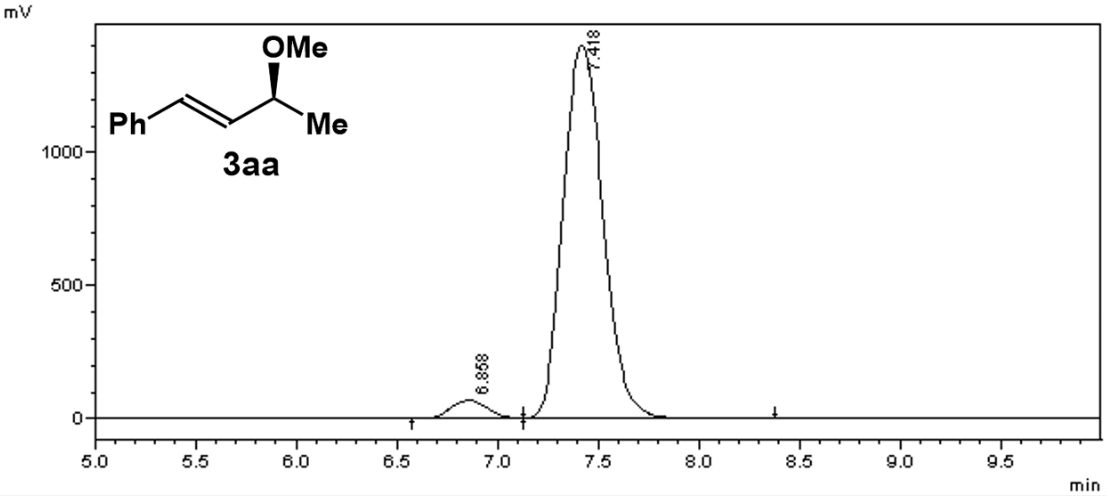

峰表

检测器 A Ch1 254nm

峰#	保留时间	面积	高度	面积 %
1	6.858	864070	67818	4.265
2	7.418	19394157	1399002	95.735

Translation of Chinese characters to English:

Peak	Retention time	Area	Height	Area %
1	6.858	864070	67818	4.265
2	7.418	19394157	1399002	95.735

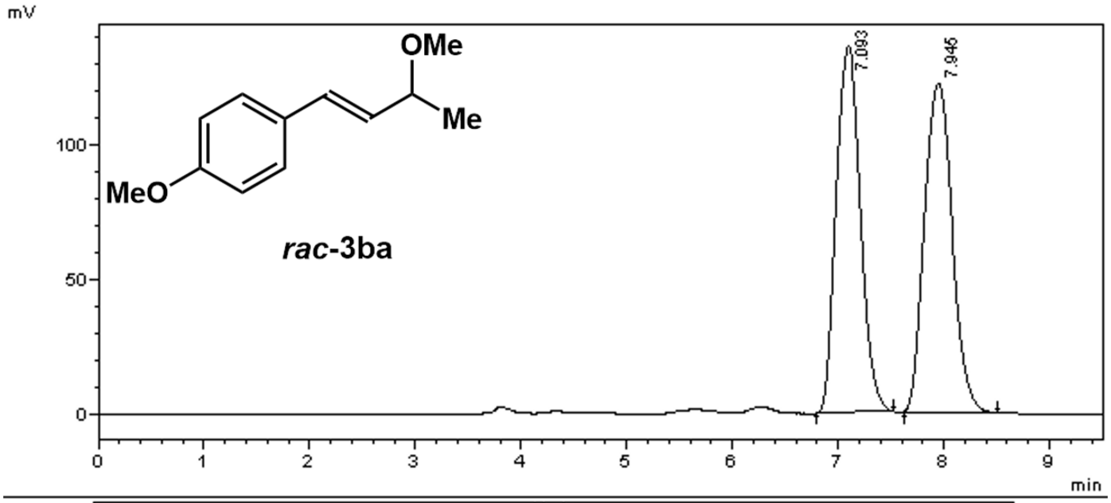

峰表

检测器 A Ch1 254nm

峰#	保留时间	面积	高度	面积 %
1	7.093	2189999	135662	49.640
2	7.945	2221778	121882	50.360

Translation of Chinese characters to English:

Peak	Retention time	Area	Height	Area %
1	7.093	2189999	135662	49.640
2	7.945	2221778	121882	50.360

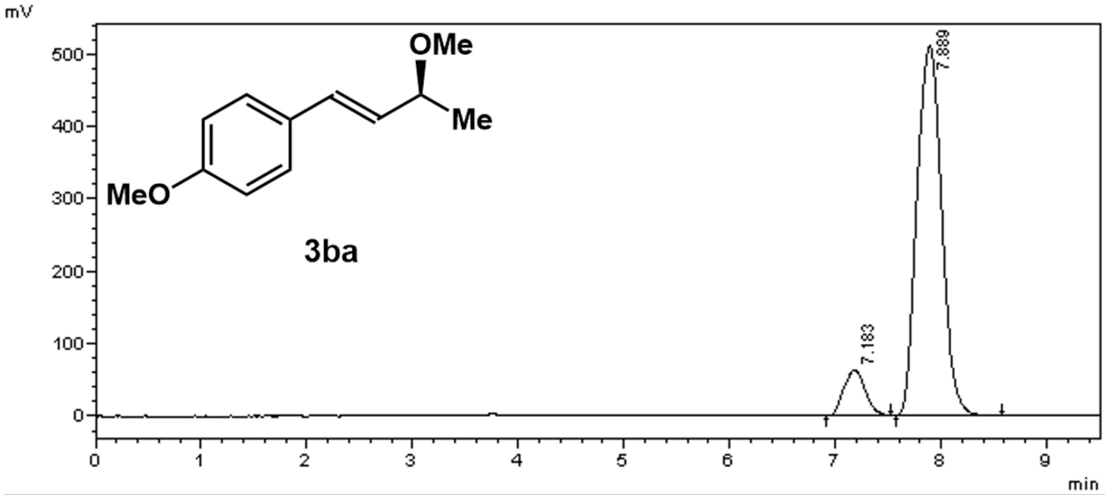

峰表

检测器 A Ch1 254nm

峰#	保留时间	面积	高度	面积 %
1	7.183	903107	62506	9.882
2	7.889	8235719	511176	90.118

Translation of Chinese characters to English:

Peak	Retention time	Area	Height	Area %
1	7.183	903107	62506	9.882
2	7.889	8235719	511176	90.118

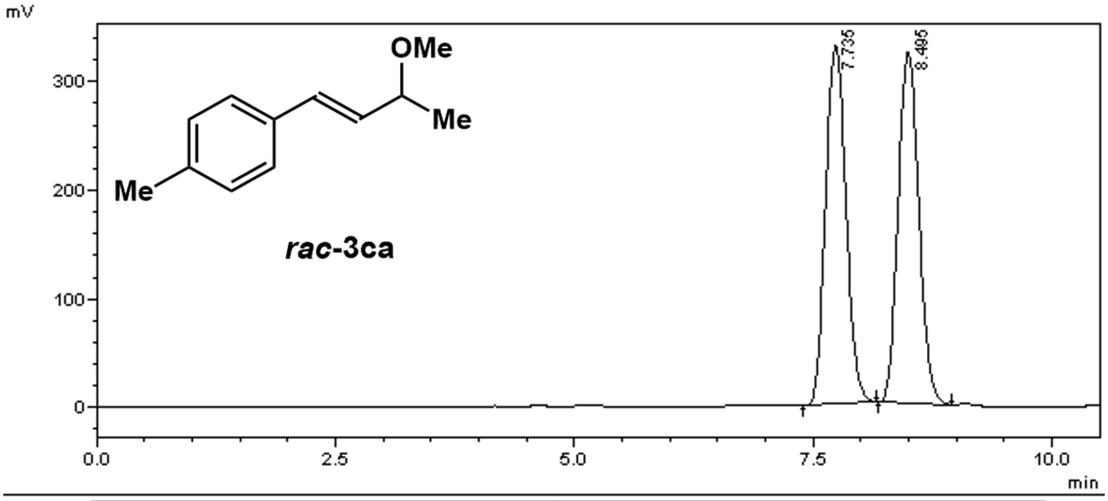

峰表

检测器 A Ch1 254nm

峰#	保留时间	面积	高度	面积 %
1	7.735	4908470	330301	50.146
2	8.495	4879876	323212	49.854

Translation of Chinese characters to English:

Peak	Retention time	Area	Height	Area %
1	7.735	4908470	330301	50.146
2	8.495	4879876	323212	49.854

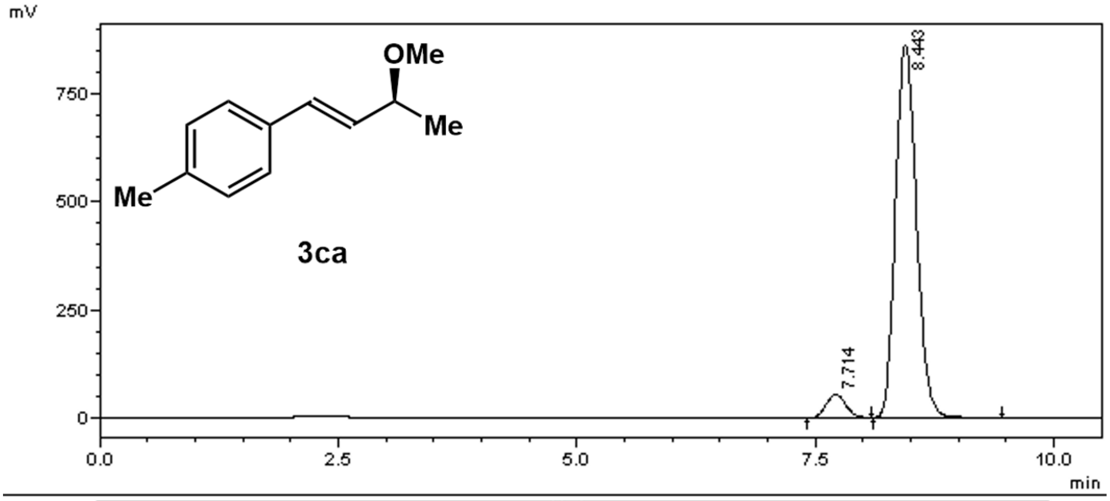

峰表

检测器 A Ch1 254nm

峰#	保留时间	面积	高度	面积 %
1	7.714	765405	53432	5.495
2	8.443	13164465	861908	94.505

Translation of Chinese characters to English:

Peak	Retention time	Area	Height	Area %
1	7.714	765405	53432	5.495
2	8.443	13164465	861908	94.505

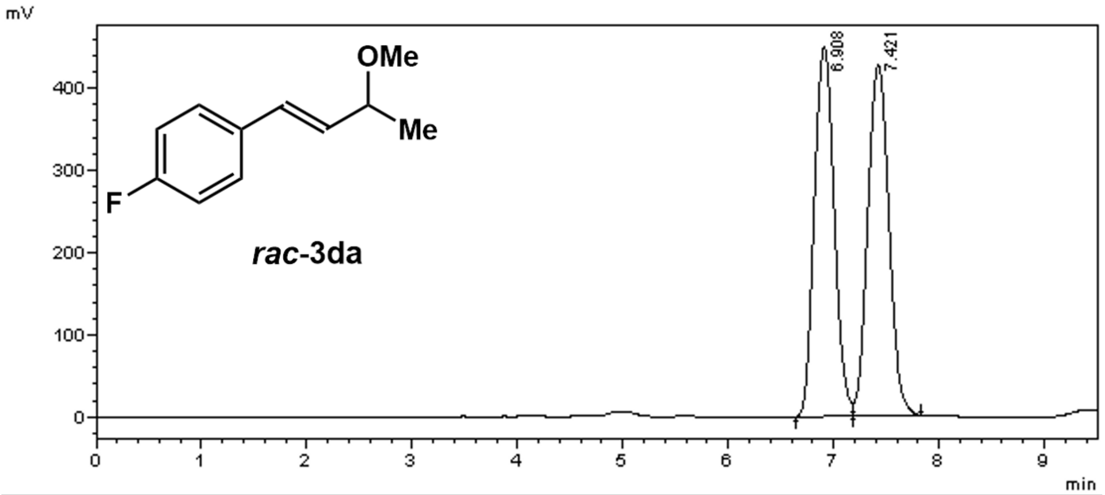

峰表

检测器 A Ch1 254nm

峰#	保留时间	面积	高度	面积 %
1	6.908	5820464	449221	49.676
2	7.421	5896483	426253	50.324

Translation of Chinese characters to English:

Peak	Retention time	Area	Height	Area %
1	6.908	5820464	449221	49.676
2	7.421	5896483	426253	50.324

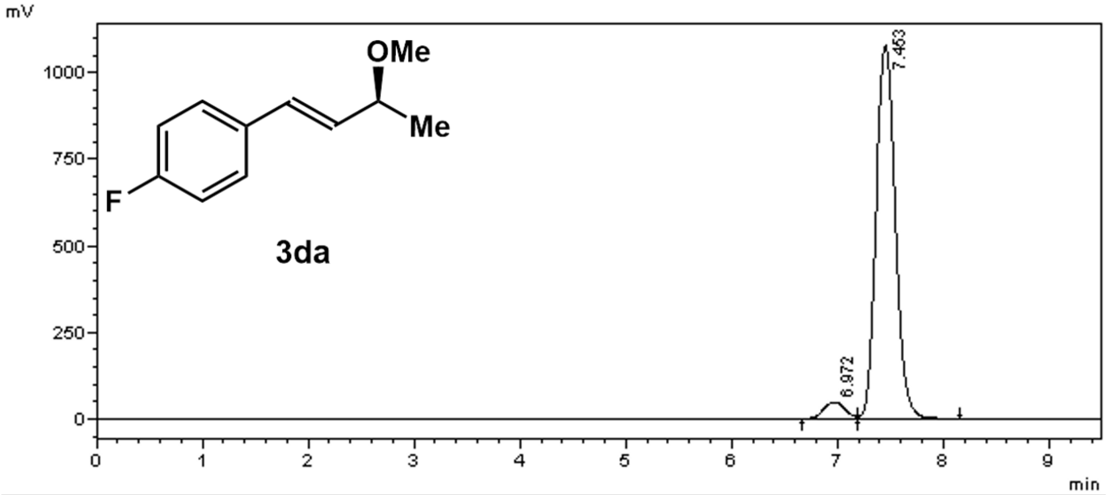

峰表

检测器 A Ch1 254nm

峰#	保留时间	面积	高度	面积 %
1	6.972	670855	47841	4.663
2	7.453	13714505	1078721	95.337

Translation of Chinese characters to English:

Peak	Retention time	Area	Height	Area %
1	6.972	670855	47841	4.663
2	7.453	13714505	1078721	95.337

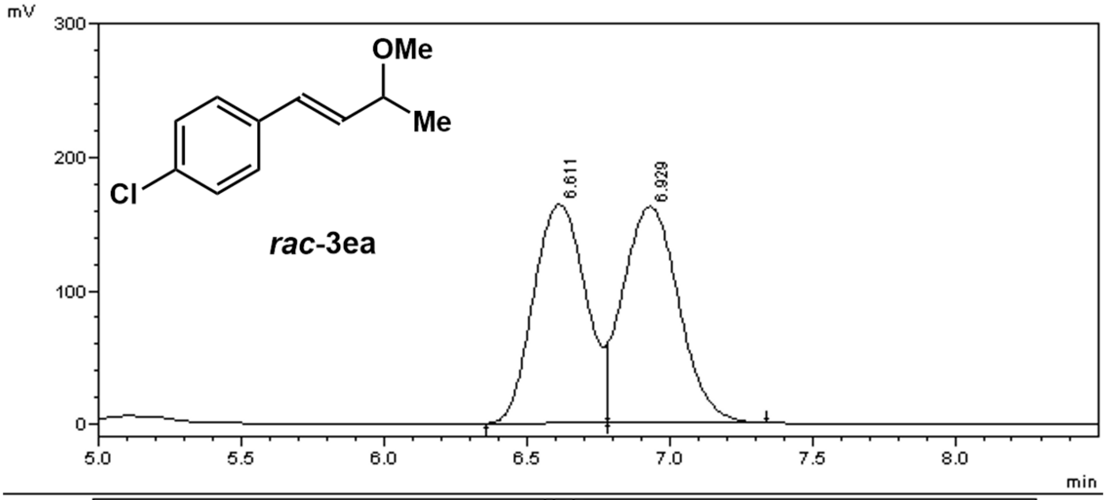

峰表

检测器 A Ch1 254nm

峰#	保留时间	面积	高度	面积 %
1	6.611	2123040	164452	49.149
2	6.929	2196522	162631	50.851

Translation of Chinese characters to English:

Peak	Retention time	Area	Height	Area %
1	6.611	2123040	164452	49.149
2	6.929	2196552	162631	50.851

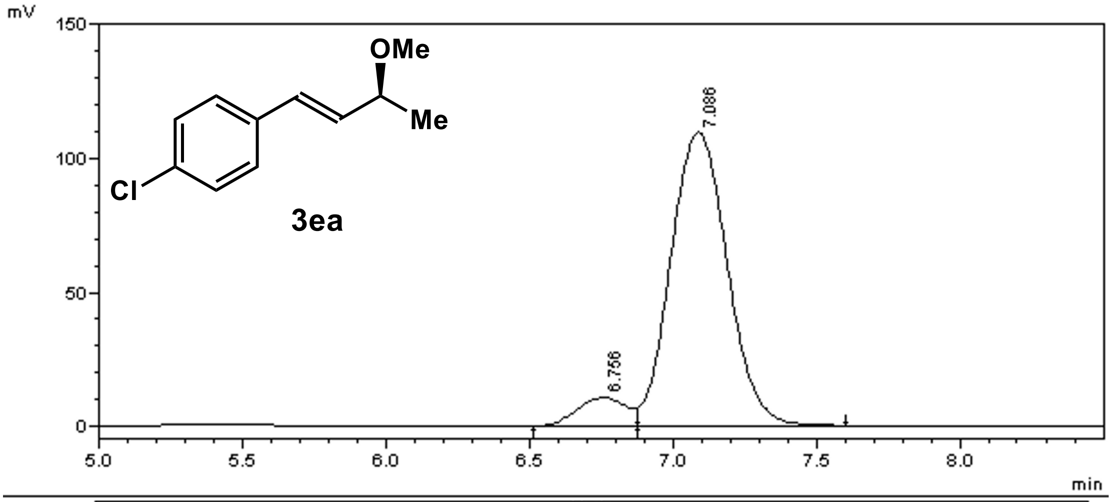

峰表

检测器 A Ch1 254nm

峰#	保留时间	面积	高度	面积 %
1	6.756	126281	10394	7.788
2	7.086	1495234	109577	92.212

Translation of Chinese characters to English:

Peak	Retention time	Area	Height	Area %
1	6.756	126281	10394	7.788
2	7.086	1495234	109577	92.212

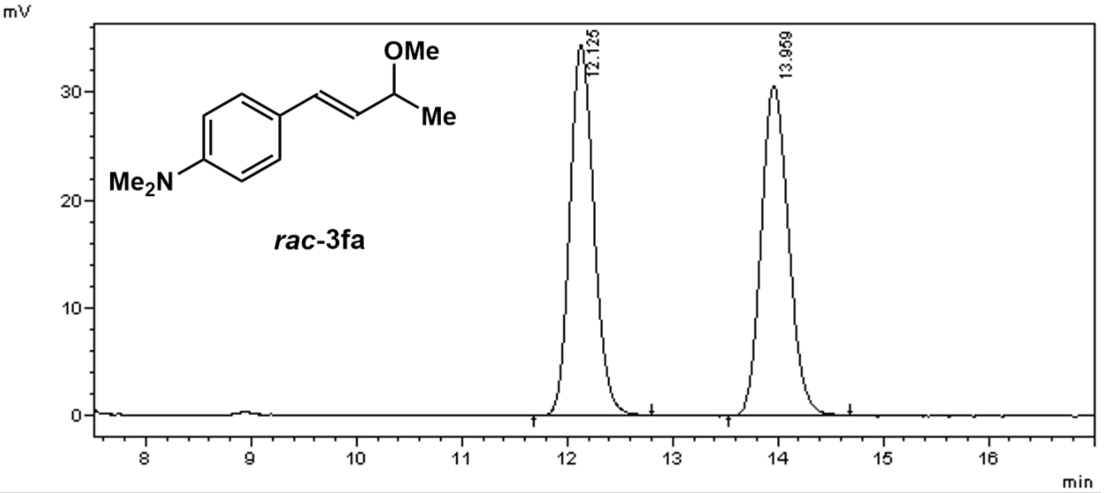

峰表

检测器 A Ch1 254nm

峰#	保留时间	面积	高度	面积 %
1	12.125	542871	34333	49.794
2	13.959	547357	30616	50.206

Translation of Chinese characters to English:

Peak	Retention time	Area	Height	Area %
1	12.125	542871	34333	49.794
2	13.959	547357	30616	50.206

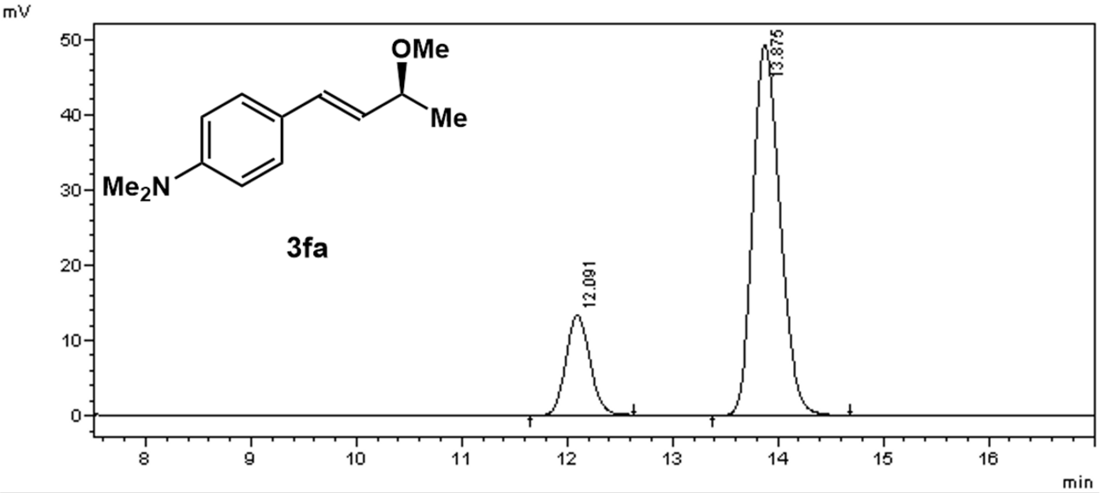

峰表

检测器 A Ch1 254nm

峰#	保留时间	面积	高度	面积 %
1	12.091	210754	13304	19.341
2	13.875	878904	49172	80.659

Translation of Chinese characters to English:

Peak	Retention time	Area	Height	Area %
1	12.091	210754	13304	19.341
2	13.875	878904	49172	80.659

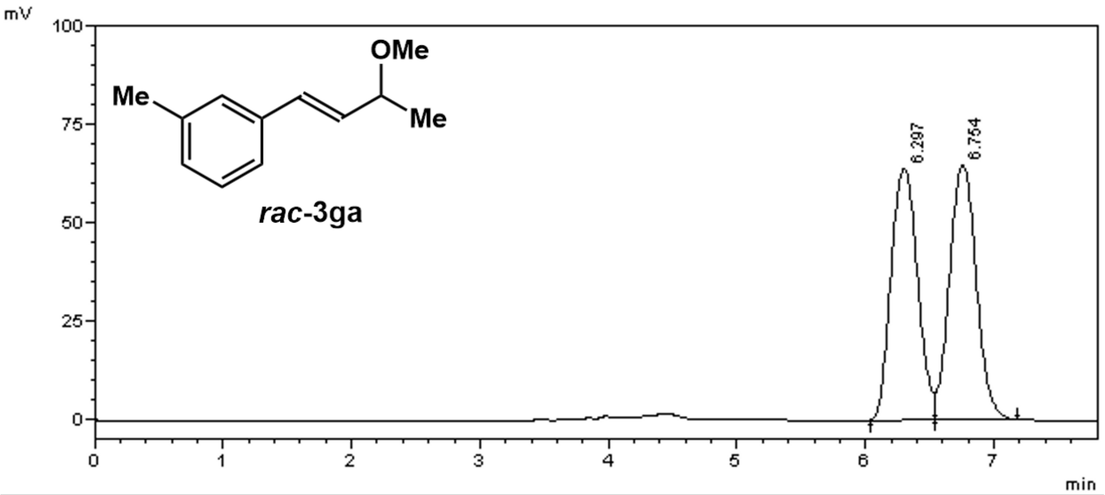

峰表

检测器 A Ch1 254nm

峰#	保留时间	面积	高度	面积 %
1	6.297	917149	64056	49.337
2	6.754	941812	64908	50.663

Translation of Chinese characters to English:

Peak	Retention time	Area	Height	Area %
1	6.297	917149	64056	49.337
2	6.754	941812	64908	50.663

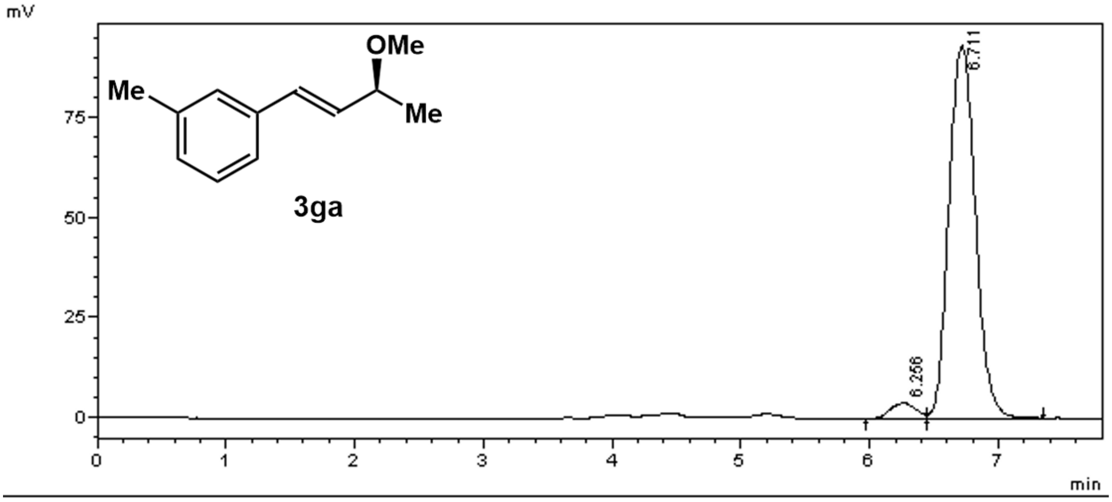

峰表

检测器 A Ch1 254nm

峰#	保留时间	面积	高度	面积 %
1	6.256	52956	3817	3.807
2	6.711	1338129	93240	96.193

Translation of Chinese characters to English:

Peak	Retention time	Area	Height	Area %
1	6.256	52956	3817	3.807
2	6.711	1338129	93240	96.193

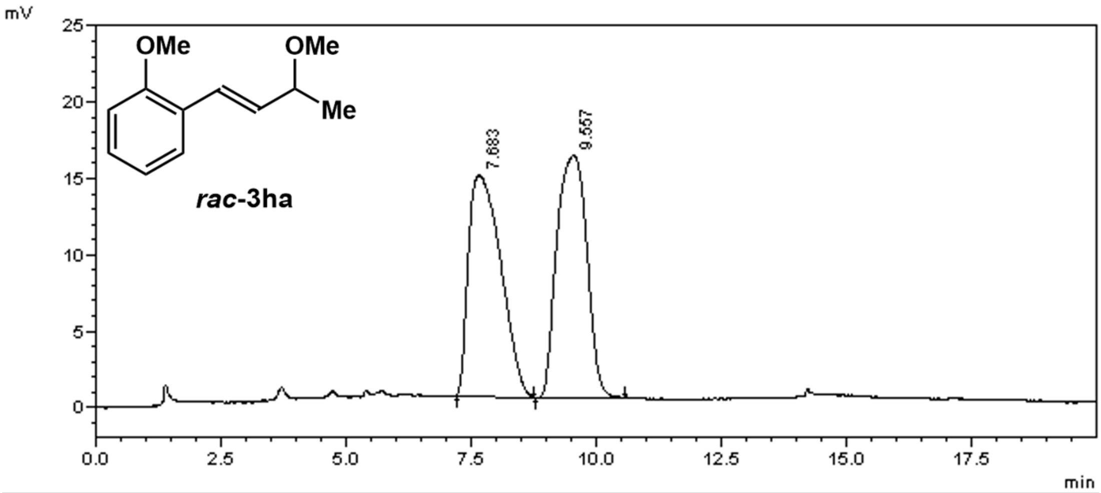

峰表

检测器 A Ch1 254nm

峰#	保留时间	面积	高度	面积 %
1	7.683	652837	14434	49.591
2	9.557	663614	15858	50.409

Translation of Chinese characters to English:

Peak	Retention time	Area	Height	Area %
1	7.683	652837	14434	49.591
2	9.557	663614	15858	50.409

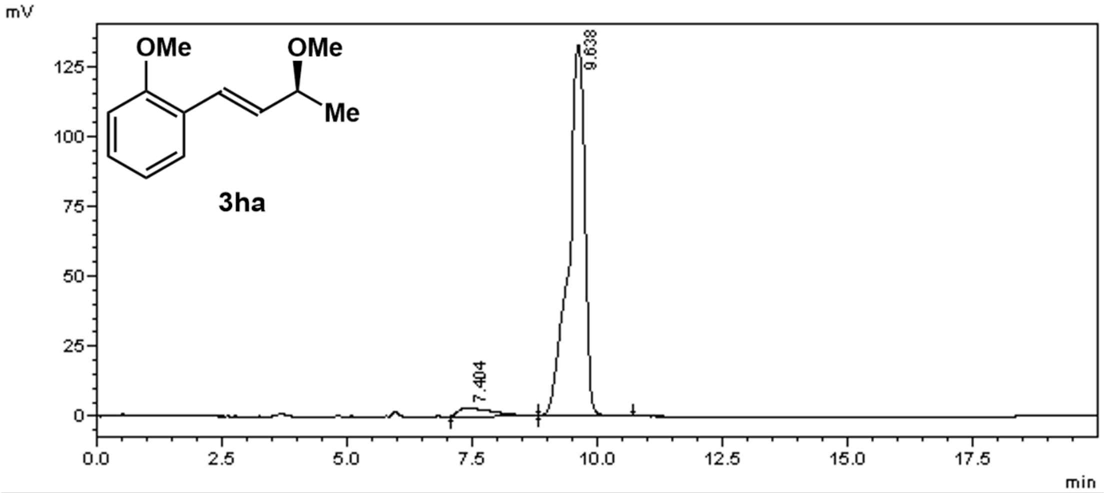

峰表

检测器 A Ch1 254nm

峰#	保留时间	面积	高度	面积 %
1	7.404	143603	2915	4.603
2	9.638	2976499	132271	95.397

Translation of Chinese characters to English:

Peak	Retention time	Area	Height	Area %
1	7.404	143603	2915	4.603
2	9.638	2976499	132271	95.397

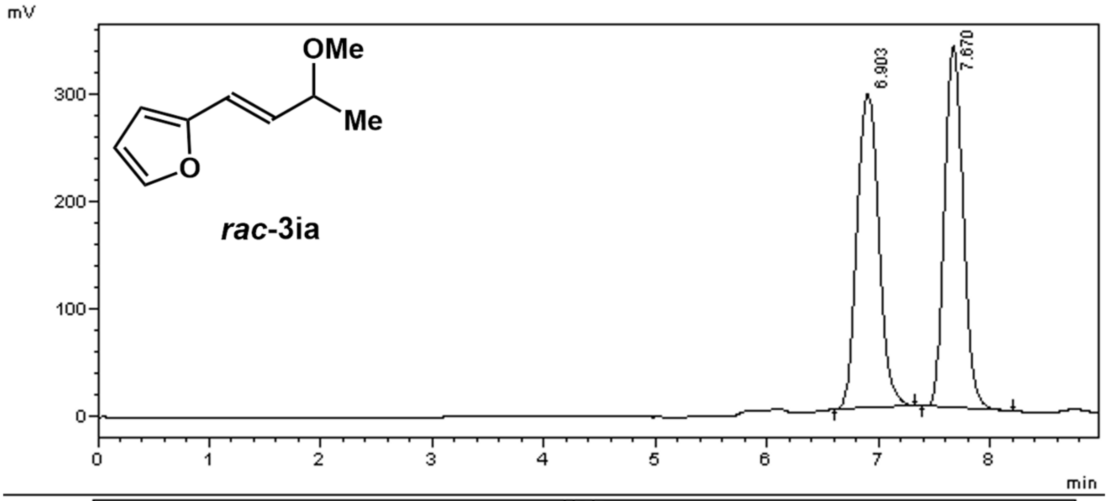

峰表

检测器 A Ch1 254nm

峰#	保留时间	面积	高度	面积 %
1	6.903	3985067	292219	50.299
2	7.670	3937662	336405	49.701

Translation of Chinese characters to English:

Peak	Retention time	Area	Height	Area %
1	6.903	3985067	292219	50.299
2	7.670	3937662	336405	49.701

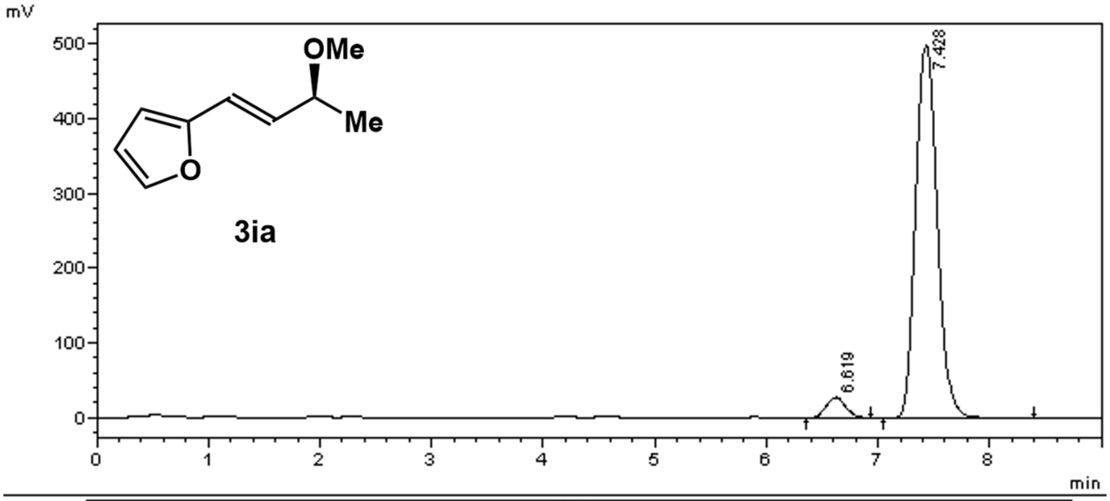

峰表

检测器 A Ch1 254nm

峰#	保留时间	面积	高度	面积 %
1	6.619	333662	27319	4.740
2	7.428	6705253	498772	95.260

Translation of Chinese characters to English:

Peak	Retention time	Area	Height	Area %
1	6.619	333662	27319	4.740
2	7.428	6705253	498772	95.260

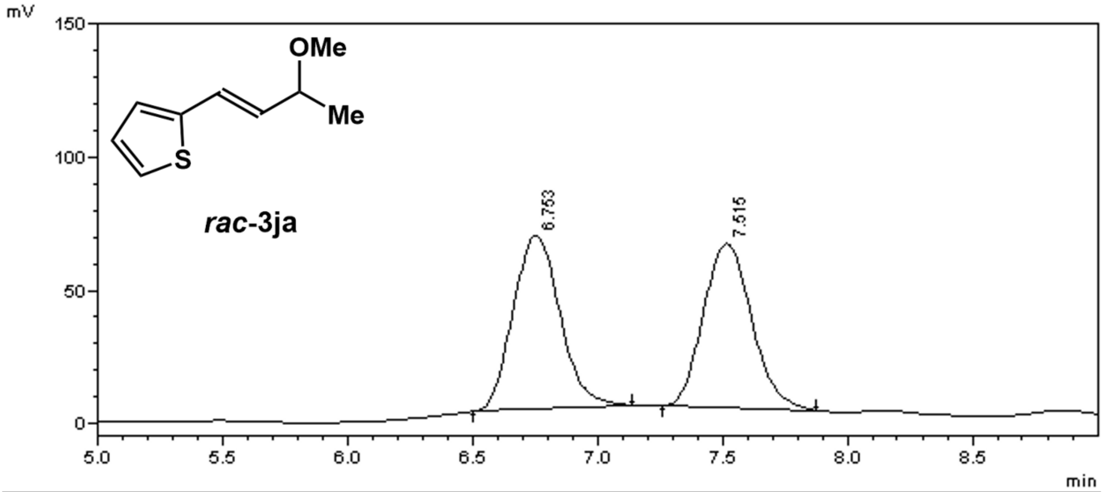

峰表

检测器 A Ch1 254nm

峰#	保留时间	面积	高度	面积 %
1	6.753	854765	65122	50.694
2	7.515	831370	61704	49.306

Translation of Chinese characters to English:

Peak	Retention time	Area	Height	Area %
1	6.753	854765	65122	50.694
2	7.515	831370	61704	49.306

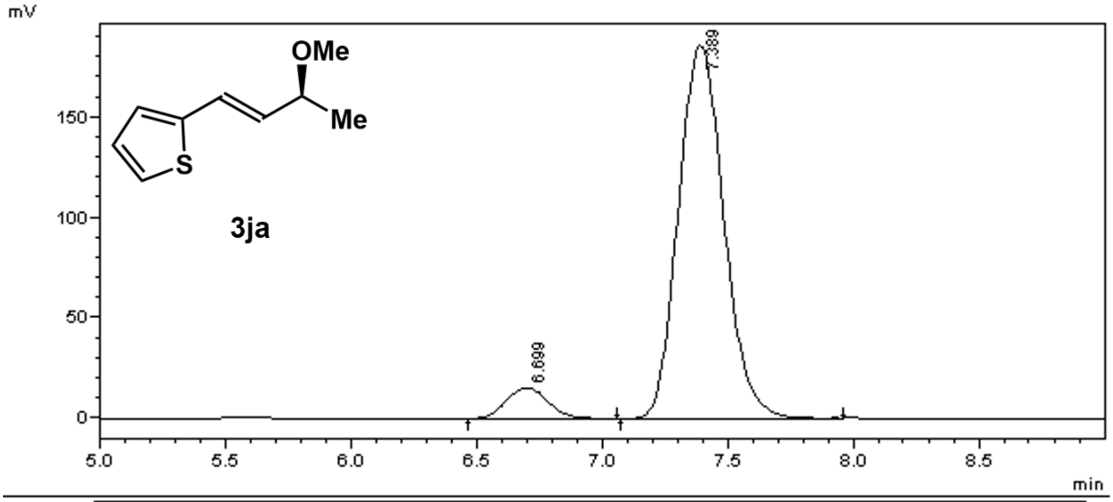

峰表

检测器 A Ch1 254nm

峰#	保留时间	面积	高度	面积 %
1	6.699	177380	15400	7.062
2	7.389	2334469	185746	92.938

Translation of Chinese characters to English:

Peak	Retention time	Area	Height	Area %
1	6.699	177380	15400	7.062
2	7.389	2334469	185746	92.938

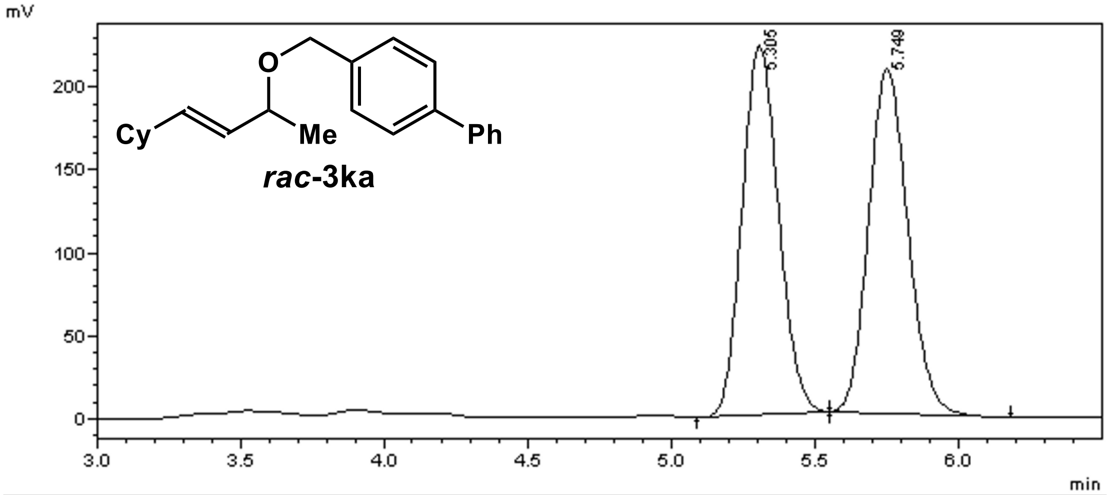

峰表

检测器 A Ch1 254nm

峰#	保留时间	面积	高度	面积 %
1	5.305	1974656	222984	49.876
2	5.749	1984489	207748	50.124

Translation of Chinese characters to English:

Peak	Retention time	Area	Height	Area %
1	5.305	1974656	222984	49.876
2	5.749	1984489	207748	50.124

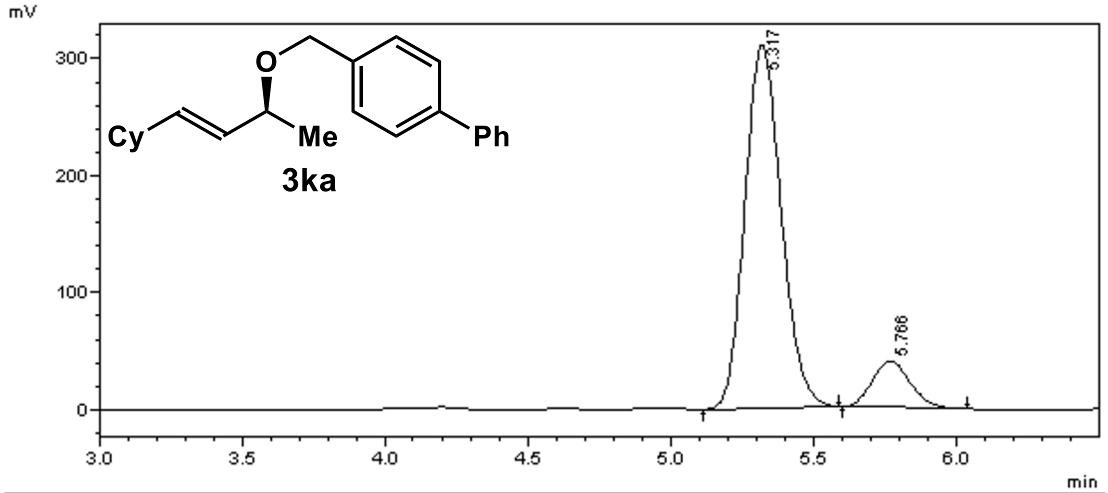

峰表

检测器 A Ch1 254nm

峰#	保留时间	面积	高度	面积 %
1	5.317	2757799	310101	88.284
2	5.766	365968	39404	11.716

Translation of Chinese characters to English:

Peak	Retention time	Area	Height	Area %
1	5.317	2757799	310101	88.284
2	5.766	365968	39404	11.716

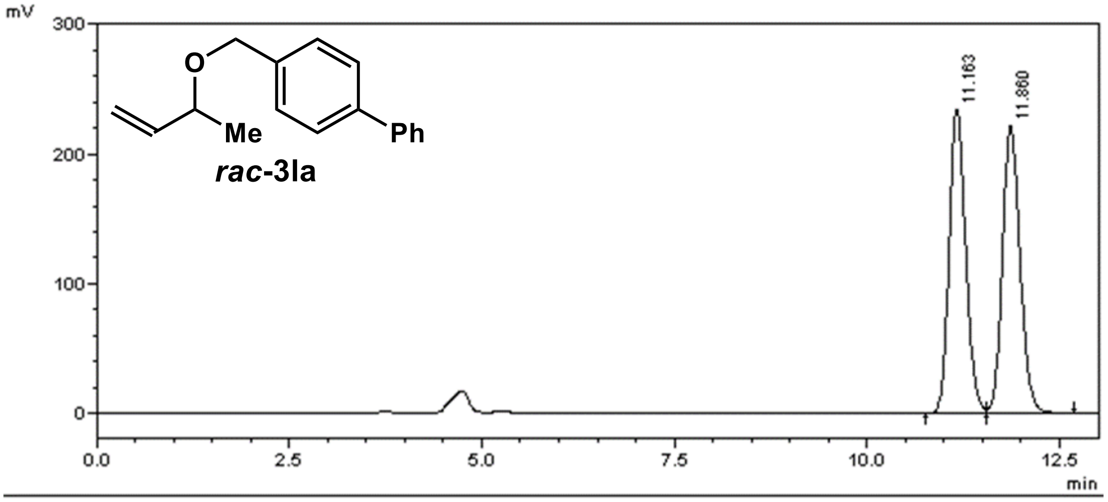

峰表

检测器 A Ch1 254nm

峰#	保留时间	面积	高度	面积 %
1	11.163	3500676	234352	49.858
2	11.860	3520673	221613	50.142

Translation of Chinese characters to English:

Peak	Retention time	Area	Height	Area %
1	11.163	3500676	234352	49.858
2	11.860	3520673	221613	50.142

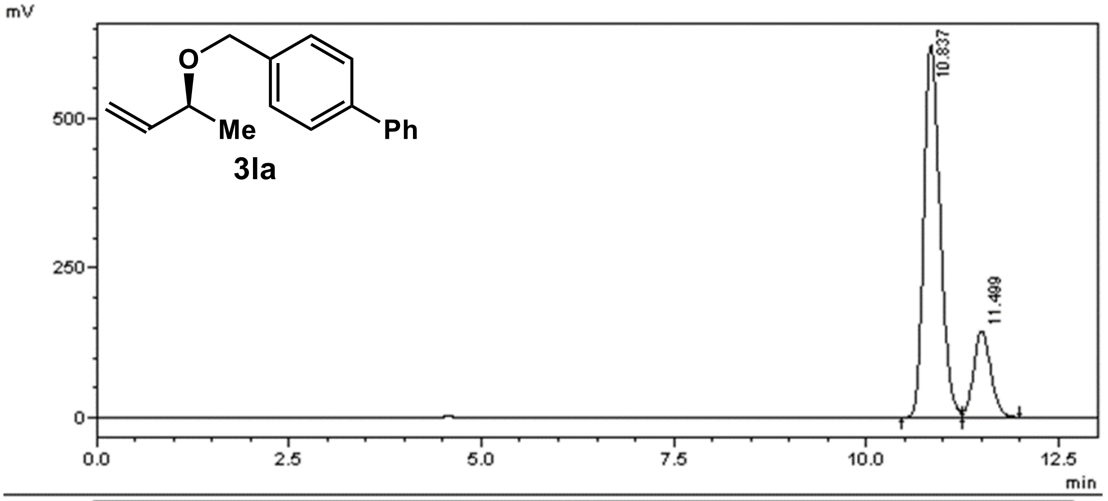

峰表

检测器 A Ch1 254nm

峰#	保留时间	面积	高度	面积 %
1	10.837	9167220	622015	80.363
2	11.499	2239977	144200	19.637

Translation of Chinese characters to English:

Peak	Retention time	Area	Height	Area %
1	10.837	9167220	622015	80.363
2	11.499	2239977	144200	19.637

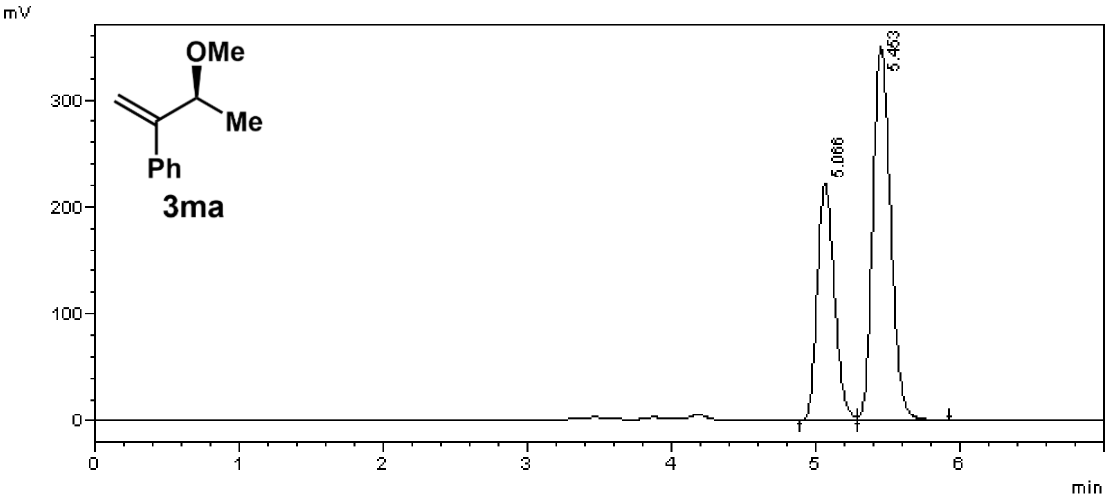

峰表

检测器 A Ch1 254nm

峰#	保留时间	面积	高度	面积 %
1	5.066	1833508	221477	37.687
2	5.453	3031566	349684	62.313

Translation of Chinese characters to English:

Peak	Retention time	Area	Height	Area %
1	5.066	1833508	221477	37.687
2	5.453	3031566	349684	62.313

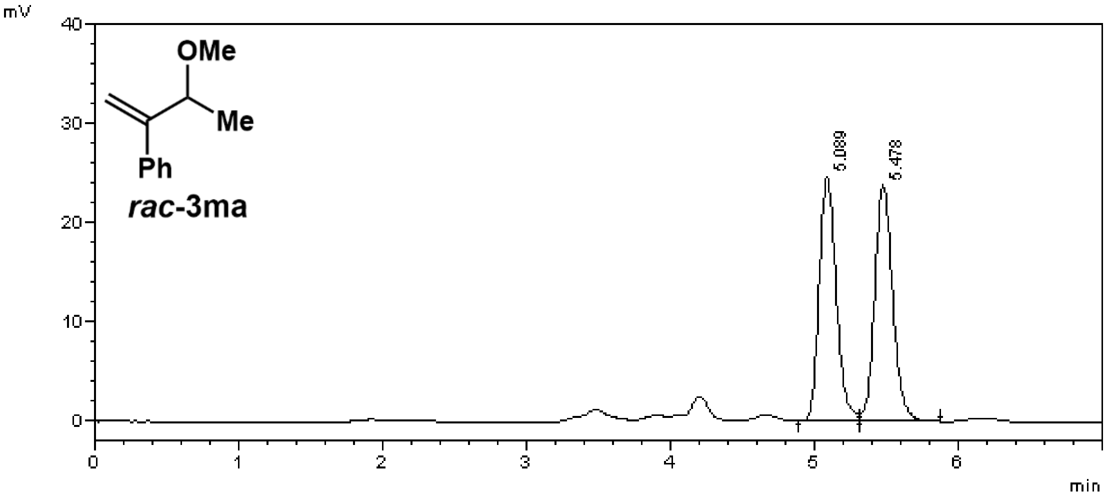

峰表

检测器 A Ch1 254nm

峰#	保留时间	面积	高度	面积 %
1	5.089	203900	24668	49.562
2	5.478	207502	23914	50.438

Translation of Chinese characters to English:

Peak	Retention time	Area	Height	Area %
1	5.089	203900	24668	49.562
2	5.478	207502	23914	50.438

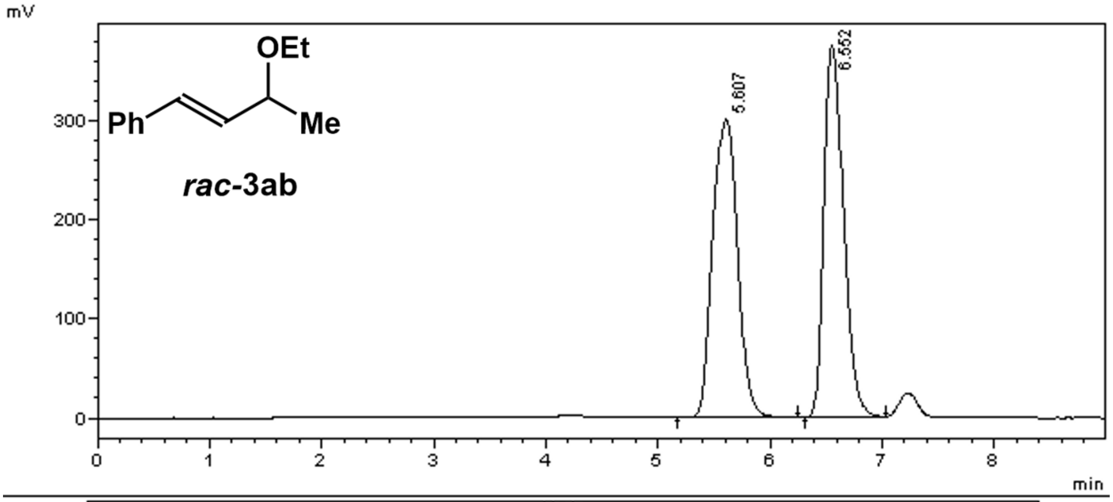

峰表

检测器 A Ch1 254nm

峰#	保留时间	面积	高度	面积 %
1	5.607	4676002	300572	50.372
2	6.552	4607002	374846	49.628

Translation of Chinese characters to English:

Peak	Retention time	Area	Height	Area %
1	5.607	4676002	300572	50.372
2	6.552	4607002	374846	49.628

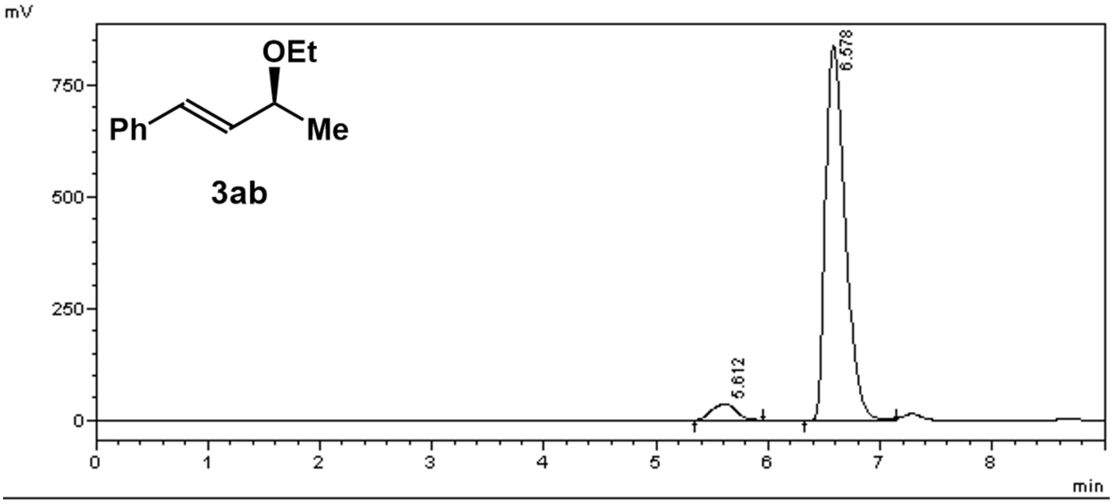

峰表

检测器 A Ch1 254nm

峰#	保留时间	面积	高度	面积 %
1	5.612	555403	35720	5.144
2	6.578	10242003	837491	94.856

Translation of Chinese characters to English:

Peak	Retention time	Area	Height	Area %
1	5.612	555403	35720	5.144
2	6.578	10242003	837491	94.856

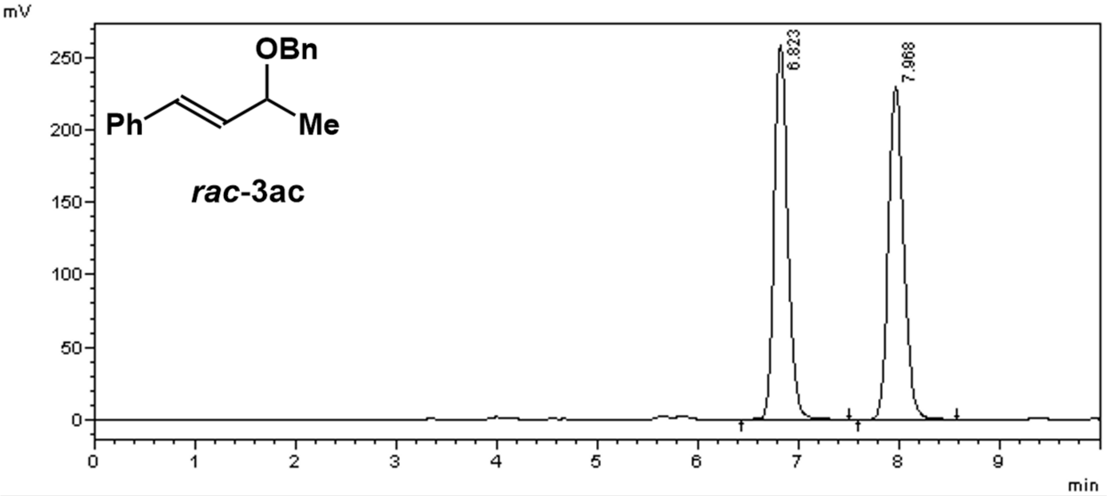

峰表
检测器 A Ch1 254nm

峰#	保留时间	面积	高度	面积 %
1	6.823	2433660	258091	50.083
2	7.968	2425594	229293	49.917

Translation of Chinese characters to English:

Peak	Retention time	Area	Height	Area %
1	6.823	2433660	258091	50.083
2	7.968	2425594	229293	49.917

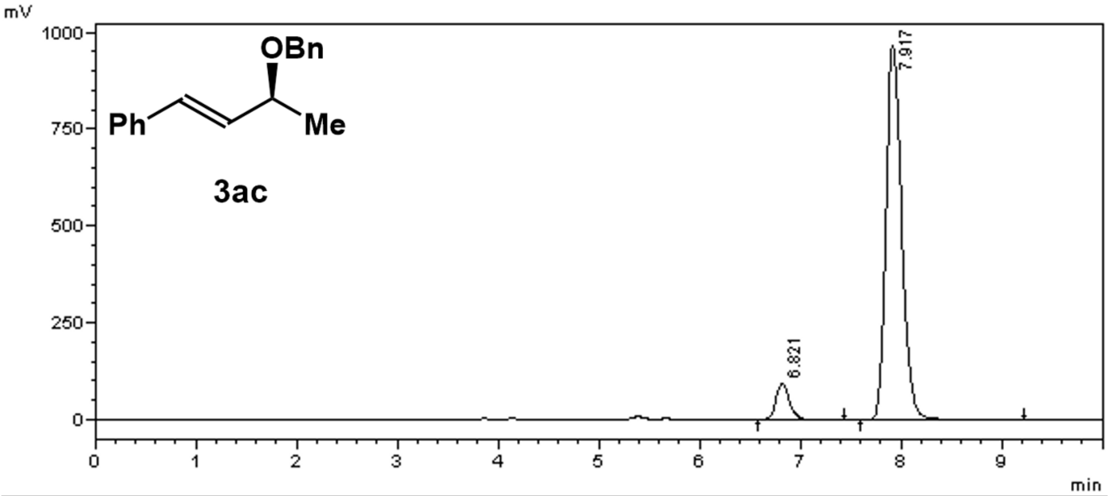

峰表
检测器 A Ch1 254nm

峰#	保留时间	面积	高度	面积 %
1	6.821	863101	92478	7.637
2	7.917	10437828	964916	92.363

Translation of Chinese characters to English:

Peak	Retention time	Area	Height	Area %
1	6.821	863101	92478	7.637
2	7.917	10437828	964916	92.363

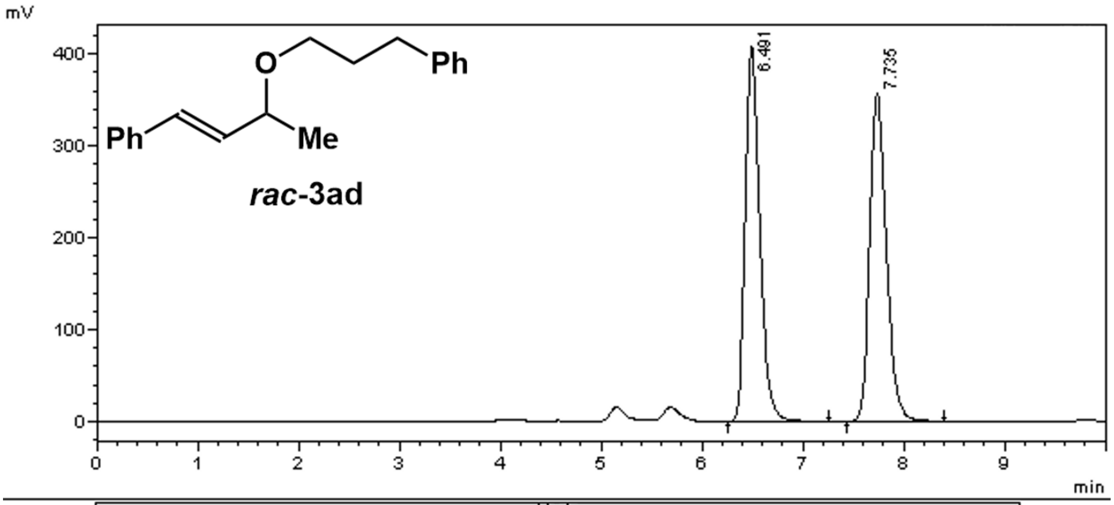

峰表

检测器 A Ch1 254nm

峰#	保留时间	面积	高度	面积 %
1	6.491	4106887	408283	49.996
2	7.735	4107480	356851	50.004

Translation of Chinese characters to English:

Peak	Retention time	Area	Height	Area %
1	6.491	4106887	408283	49.996
2	7.735	4107480	356851	50.004

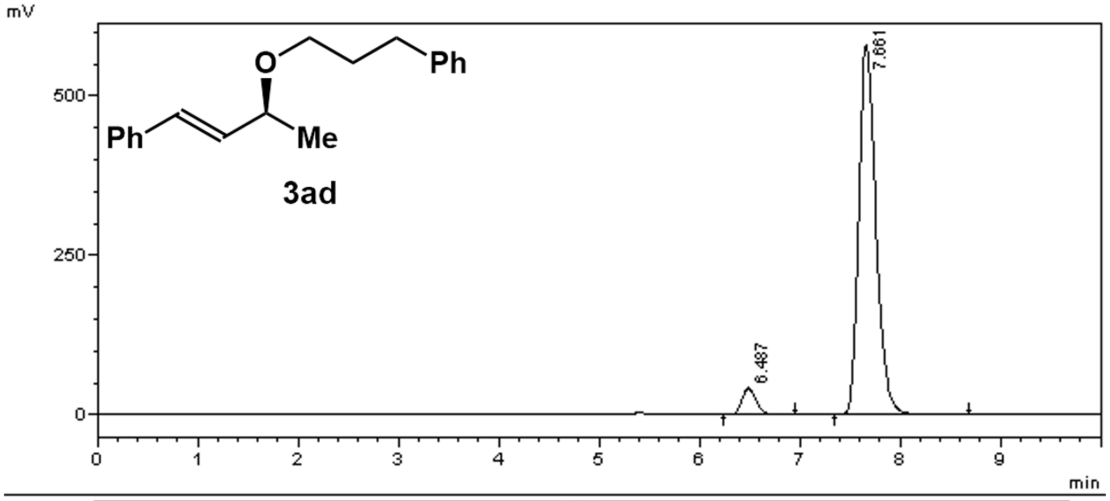

峰表

检测器 A Ch1 254nm

峰#	保留时间	面积	高度	面积 %
1	6.487	412972	41759	5.636
2	7.661	6914198	579719	94.364

Translation of Chinese characters to English:

Peak	Retention time	Area	Height	Area %
1	6.487	412972	41759	5.636
2	7.661	6914198	579719	94.364

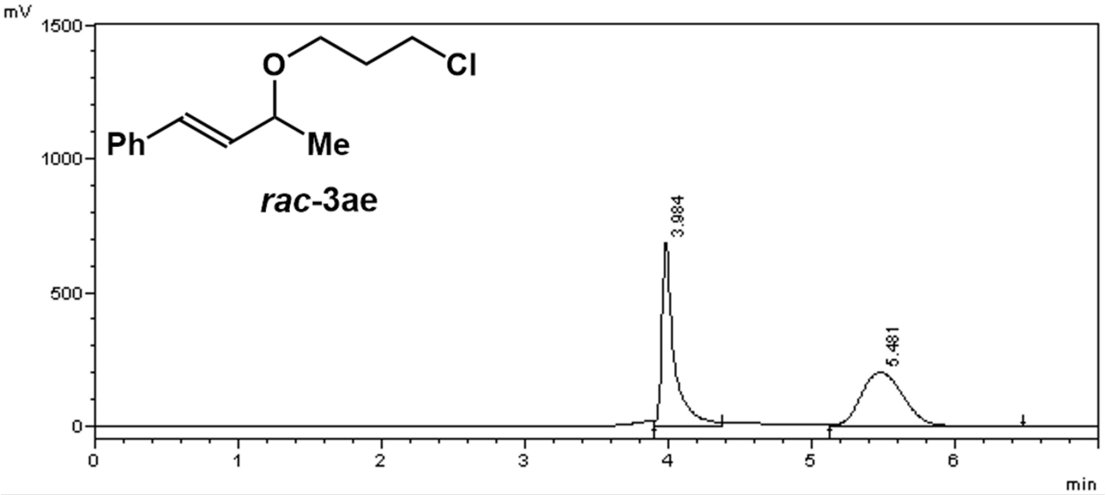

峰表

检测器 A Ch1 254nm

峰#	保留时间	面积	高度	面积 %
1	3.984	4117332	683702	50.098
2	5.481	4101230	200694	49.902

Translation of Chinese characters to English:

Peak	Retention time	Area	Height	Area %
1	3.984	4117332	683702	50.098
2	5.481	4101230	200694	49.902

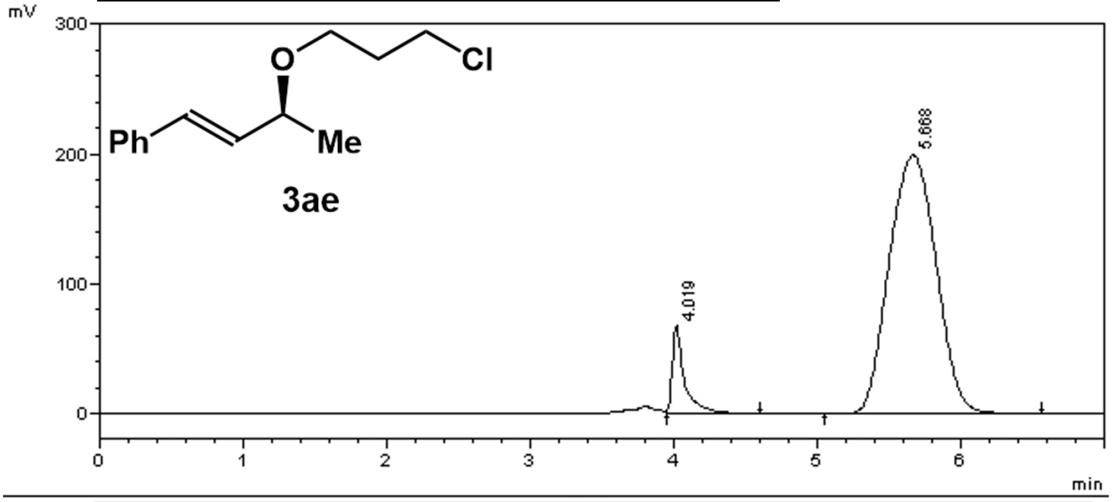

峰表

检测器 A Ch1 254nm

峰#	保留时间	面积	高度	面积 %
1	4.019	405981	68164	8.231
2	5.668	4526268	198950	91.769

Translation of Chinese characters to English:

Peak	Retention time	Area	Height	Area %
1	4.019	405981	68164	8.231
2	5.668	4526268	198950	91.769

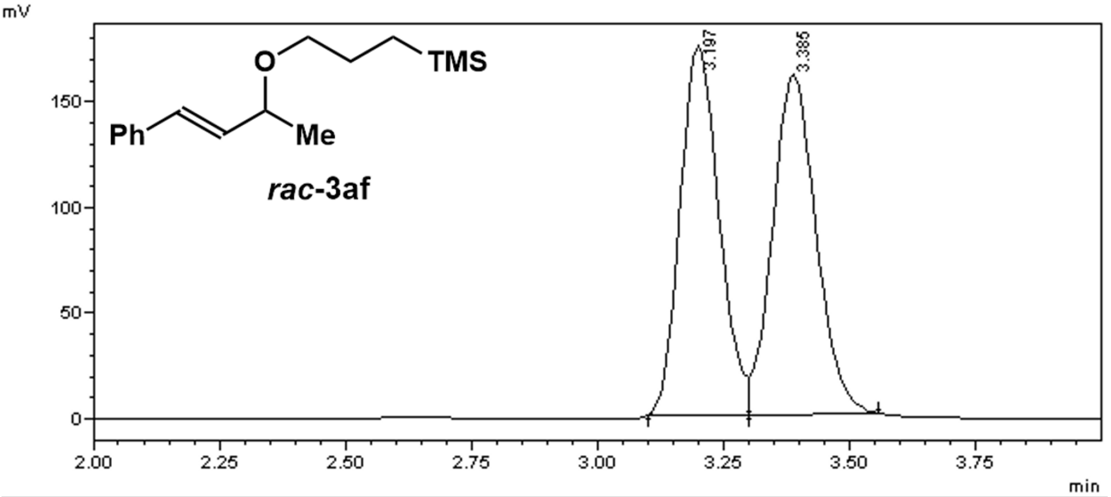

峰表

检测器 A Ch1 254nm

峰#	保留时间	面积	高度	面积 %
1	3.197	946509	175278	49.531
2	3.385	964446	160650	50.469

Translation of Chinese characters to English:

Peak	Retention time	Area	Height	Area %
1	3.197	946509	175278	49.531
2	3.385	964446	160650	50.469

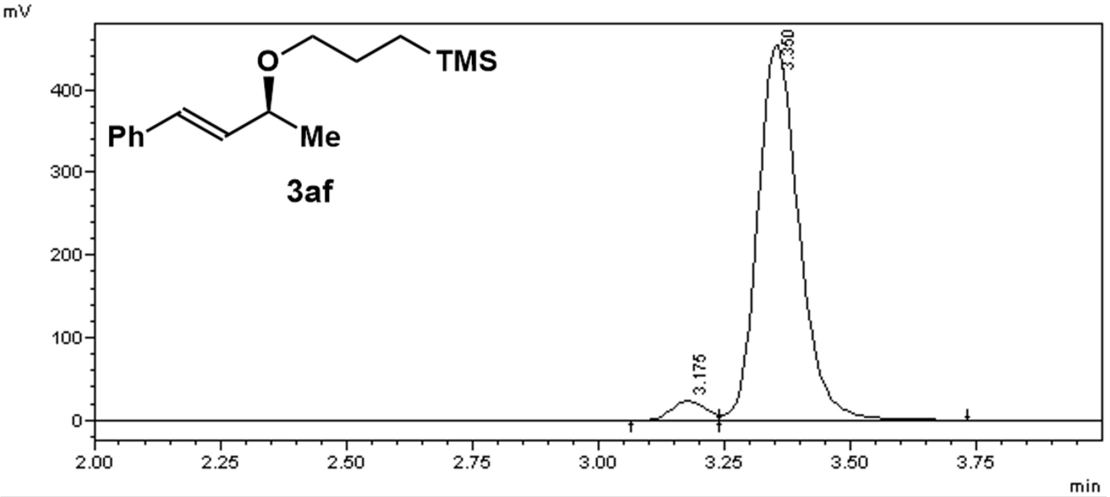

峰表

检测器 A Ch1 254nm

峰#	保留时间	面积	高度	面积 %
1	3.175	113213	23482	4.205
2	3.350	2578891	453904	95.795

Translation of Chinese characters to English:

Peak	Retention time	Area	Height	Area %
1	3.175	113213	23482	4.205
2	3.350	2578891	453904	95.795

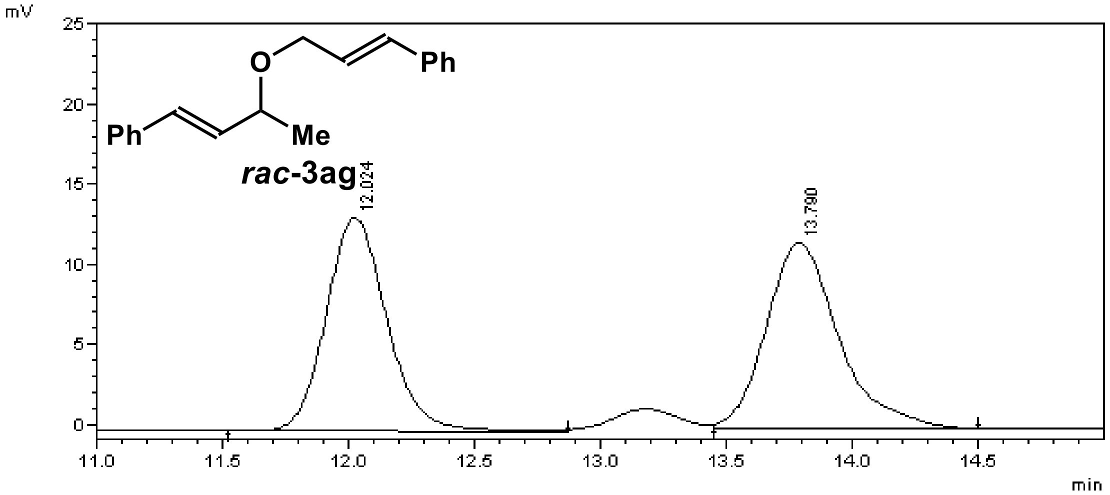

峰表

检测器 A Ch1 254nm

峰#	保留时间	面积	高度	面积 %
1	12.024	225111	13379	49.146
2	13.790	232932	11609	50.854

Translation of Chinese characters to English:

Peak	Retention time	Area	Height	Area %
1	12.024	225111	13379	49.146
2	13.790	232932	11609	50.854

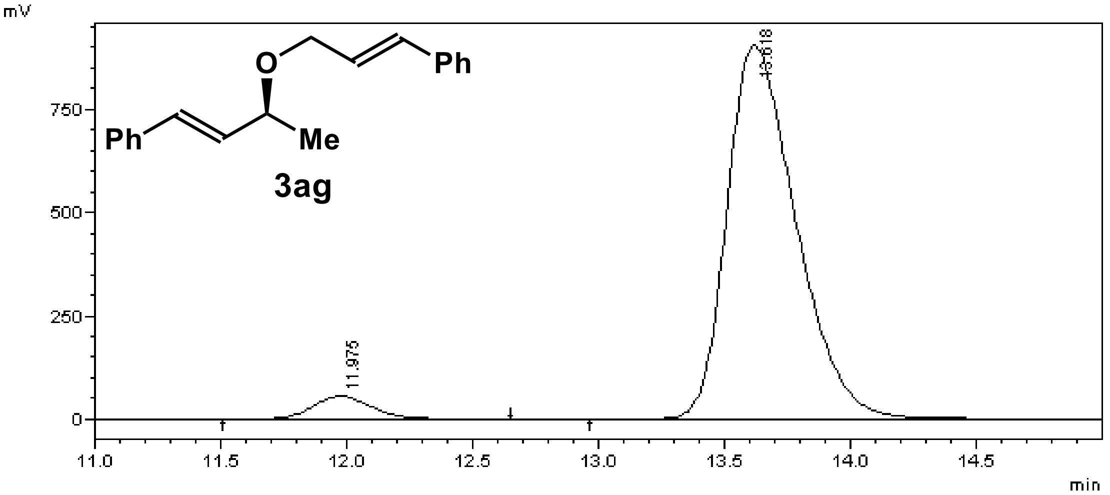

峰表

检测器 A Ch1 254nm

峰#	保留时间	面积	高度	面积 %
1	11.975	905593	55633	4.975
2	13.618	17298438	905621	95.025

Translation of Chinese characters to English:

Peak	Retention time	Area	Height	Area %
1	11.975	905593	55633	4.975
2	13.618	17298438	905621	95.025

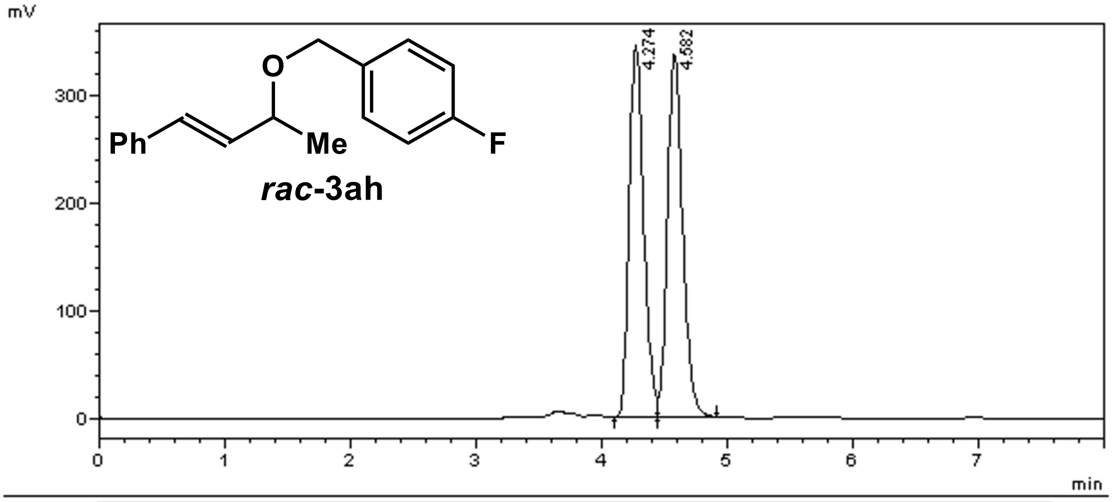

峰表

检测器 A Ch1 254nm

峰#	保留时间	面积	高度	面积 %
1	4.274	2798667	345630	49.488
2	4.582	2856609	337114	50.512

Translation of Chinese characters to English:

Peak	Retention time	Area	Height	Area %
1	4.274	2798667	345630	49.488
2	4.582	2856609	337114	50.512

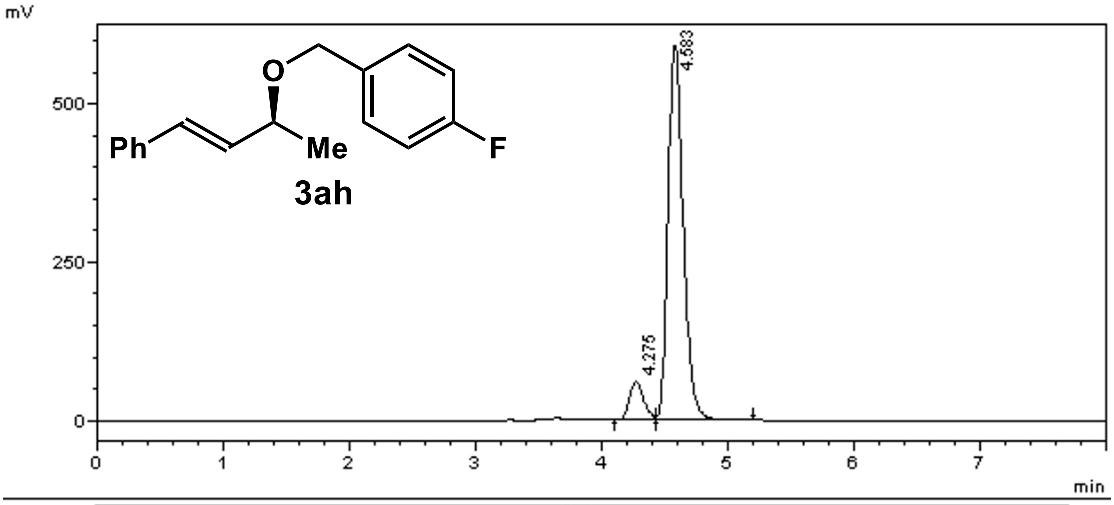

峰表

检测器 A Ch1 254nm

峰#	保留时间	面积	高度	面积 %
1	4.275	481397	60121	8.796
2	4.583	4991515	591950	91.204

Translation of Chinese characters to English:

Peak	Retention time	Area	Height	Area %
1	4.275	481397	60121	8.796
2	4.583	4991515	591950	91.204

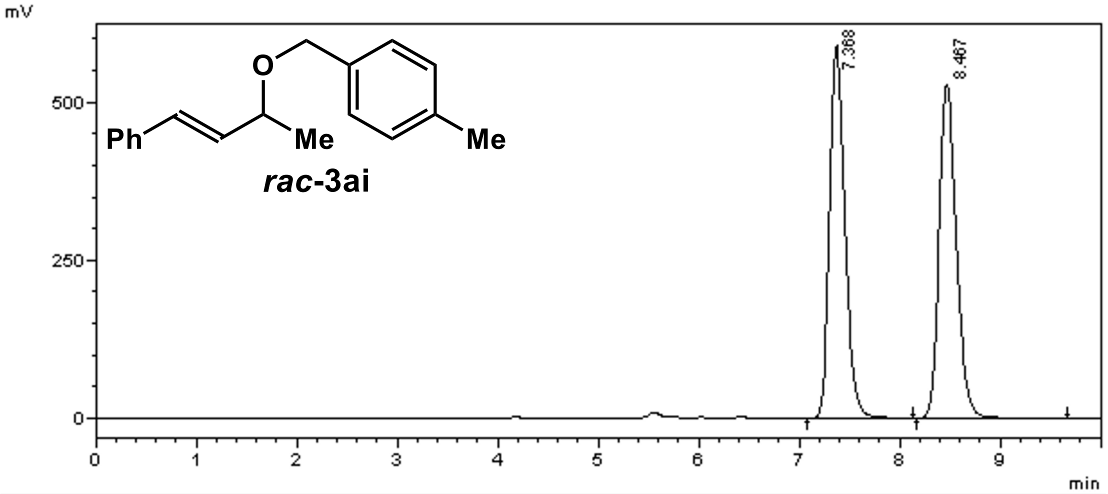

峰表

检测器 A Ch1 254nm

峰#	保留时间	面积	高度	面积 %
1	7.368	6282289	589147	50.019
2	8.467	6277598	527205	49.981

Translation of Chinese characters to English:

Peak	Retention time	Area	Height	Area %
1	7.368	6282289	589147	50.019
2	8.467	6277598	527205	49.981

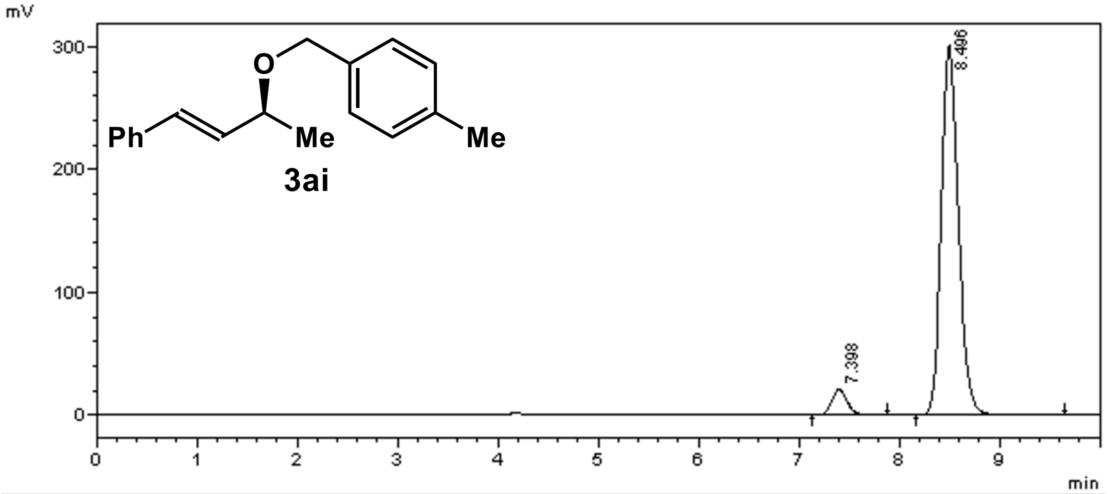

峰表

检测器 A Ch1 254nm

峰#	保留时间	面积	高度	面积 %
1	7.398	225804	21101	5.931
2	8.496	3581684	301132	94.069

Translation of Chinese characters to English:

Peak	Retention time	Area	Height	Area %
1	7.398	225804	21101	5.931
2	8.496	3581684	301132	94.069

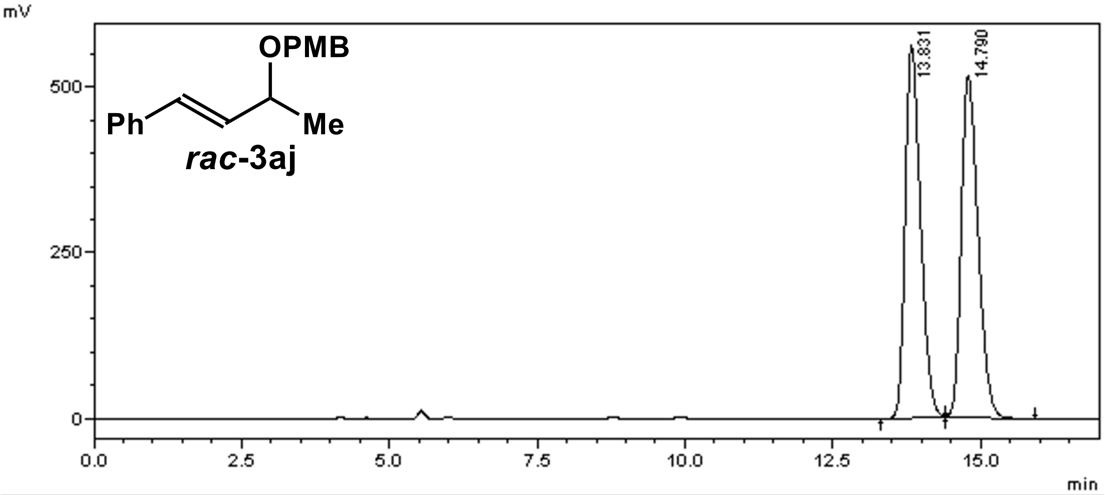

峰表

检测器 A Ch1 254nm

峰#	保留时间	面积	高度	面积 %
1	13.831	10088376	560354	49.999
2	14.790	10088632	513245	50.001

Translation of Chinese characters to English:

Peak	Retention time	Area	Height	Area %
1	13.831	10088376	560354	49.999
2	14.790	10088672	513245	50.001

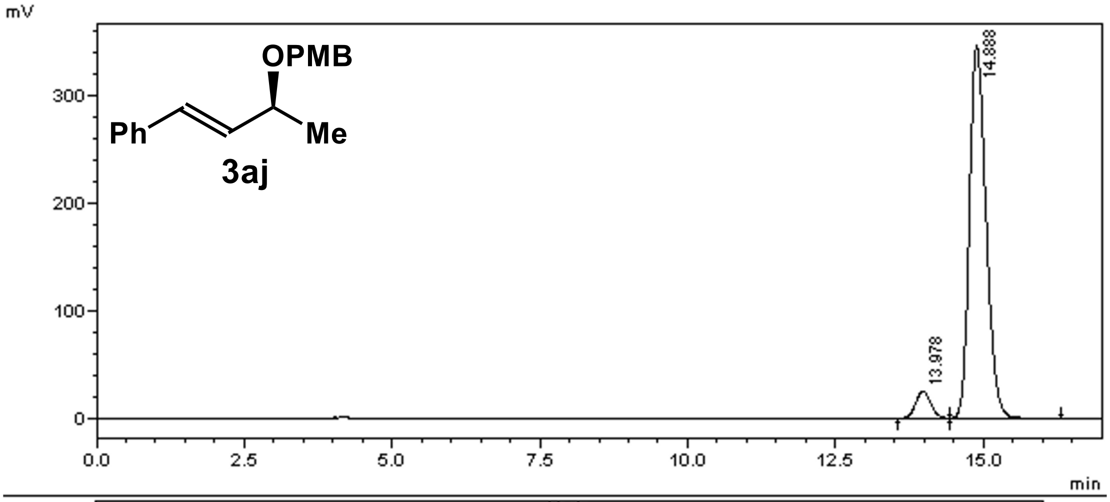

峰表

检测器 A Ch1 254nm

峰#	保留时间	面积	高度	面积 %
1	13.978	443909	25031	6.167
2	14.888	6754301	346898	93.833

Translation of Chinese characters to English:

Peak	Retention time	Area	Height	Area %
1	13.978	443909	25031	6.167
2	14.888	6754301	346898	93.833

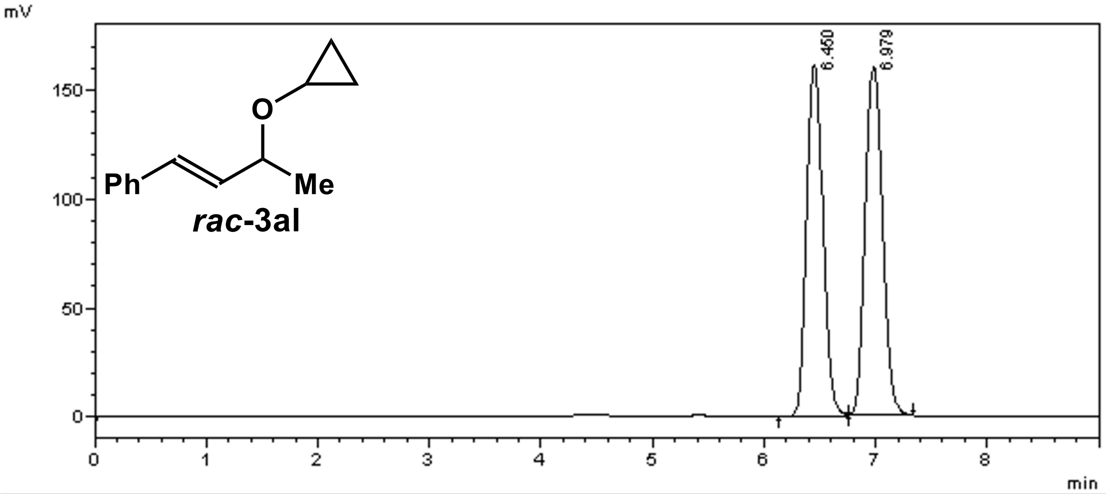

峰表

检测器 A Ch1 254nm

峰#	保留时间	面积	高度	面积 %
1	6.450	1670584	161731	49.217
2	6.979	1723708	159624	50.783

Translation of Chinese characters to English:

Peak	Retention time	Area	Height	Area %
1	6.450	1670584	161731	49.217
2	6.979	1723708	159624	50.783

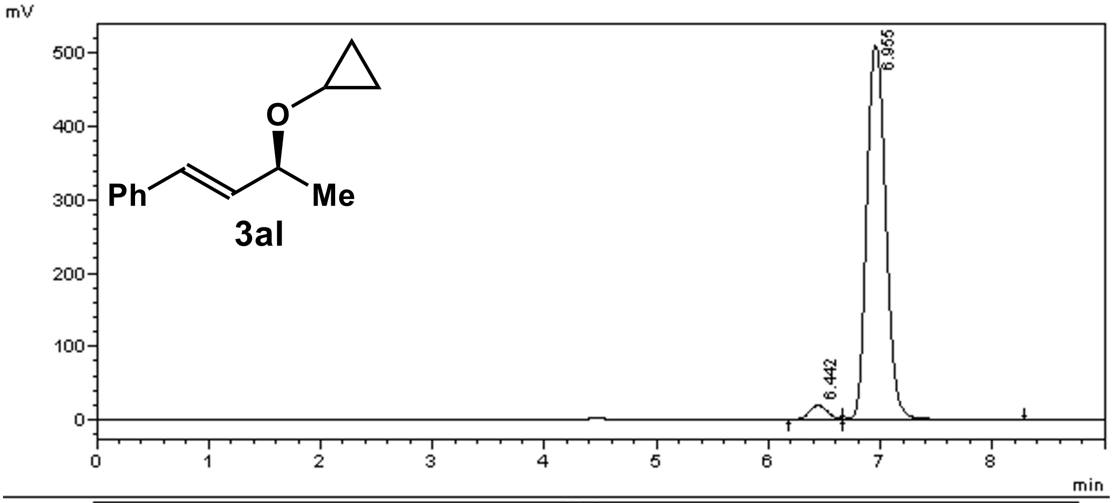

峰表

检测器 A Ch1 254nm

峰#	保留时间	面积	高度	面积 %
1	6.442	213582	18806	3.430
2	6.955	6013512	510793	96.570

Translation of Chinese characters to English:

Peak	Retention time	Area	Height	Area %
1	6.442	213582	18806	3.430
2	6.955	6013512	510793	96.570

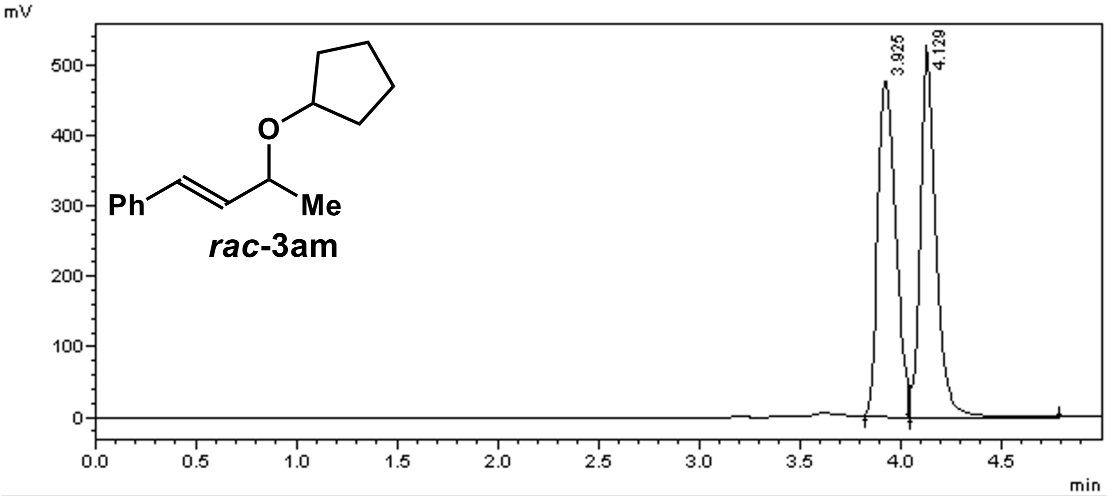

峰表

检测器 A Ch1 254nm

峰#	保留时间	面积	高度	面积 %
1	3.925	2936891	477226	50.274
2	4.129	2904933	527581	49.726

Translation of Chinese characters to English:

Peak	Retention time	Area	Height	Area %
1	3.925	2936891	477226	50.274
2	4.129	2904933	527581	49.726

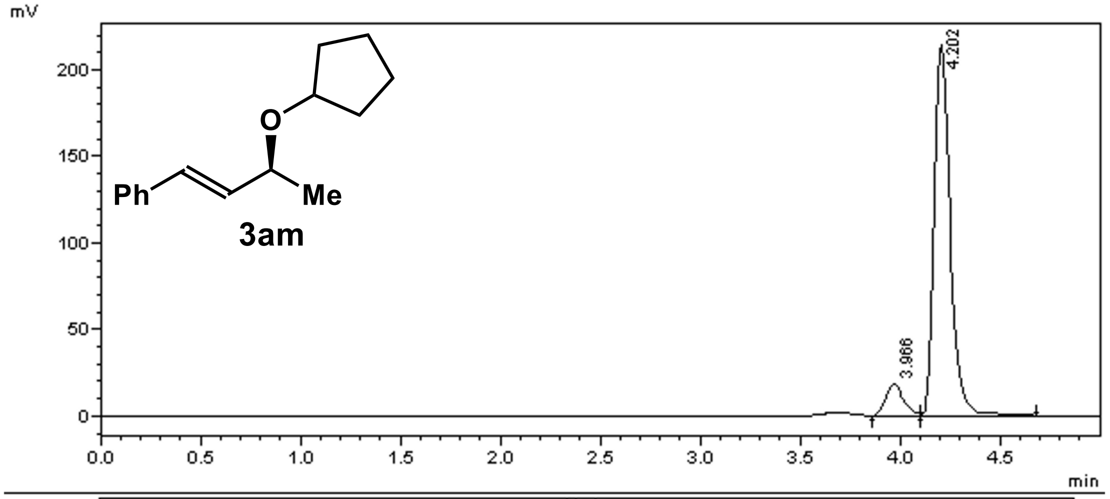

峰表

检测器 A Ch1 254nm

峰#	保留时间	面积	高度	面积 %
1	3.966	109192	17678	8.525
2	4.202	1171682	214010	91.475

Translation of Chinese characters to English:

Peak	Retention time	Area	Height	Area %
1	3.966	109192	17678	8.525
2	4.202	1171682	214010	91.475
